# Constitutively-stressed yeast strains are high-yielding for recombinant Fps1: implications for the translational regulation of an aquaporin

**DOI:** 10.1186/s12934-017-0656-2

**Published:** 2017-03-09

**Authors:** Stephanie P. Cartwright, Richard A. J. Darby, Debasmita Sarkar, Nicklas Bonander, Stephane R. Gross, Mark P. Ashe, Roslyn M. Bill

**Affiliations:** 1 School of Life and Health Sciences, Aston University, Aston Triangle, Birmingham, B4 7ET UK; 2 Thistle Scientific Ltd, Glasgow, G71 6NZ UK; 3 Department of Biology and Biological Engineering, Chalmers University of Technology, Gothenburg, 41296 Sweden; 4 Faculty of Life Sciences, University of Manchester, Manchester, M13 9PT UK

**Keywords:** Recombinant protein, *Saccharomyces cerevisiae*, Upstream open reading frame, Translation initiation, Leaky scanning, Fps1 regulation, Aquaglyceroporin

## Abstract

**Background:**

We previously selected four strains of *Saccharomyces cerevisiae* for their ability to produce the aquaporin Fps1 in sufficient yield for further study. Yields from the yeast strains *spt3Δ*, *srb5Δ*, *gcn5Δ* and yTHC*BMS1* (supplemented with 0.5 μg/mL doxycycline) that had been transformed with an expression plasmid containing 249 base pairs of 5′ untranslated region (UTR) in addition to the primary *FPS1* open reading frame (ORF) were 10–80 times higher than yields from wild-type cells expressing the same plasmid. One of the strains increased recombinant yields of the G protein-coupled receptor adenosine receptor 2a (A_2a_R) and soluble green fluorescent protein (GFP). The specific molecular mechanisms underpinning a high-yielding Fps1 phenotype remained incompletely described.

**Results:**

Polysome profiling experiments were used to analyze the translational state of *spt3Δ*, *srb5Δ*, *gcn5Δ* and yTHC*BMS1* (supplemented with 0.5 μg/mL doxycycline); all but *gcn5Δ* were found to exhibit a clear block in translation initiation. Four additional strains with known initiation blocks (*rpl31aΔ*, *rpl22aΔ*, *ssf1Δ* and *nop1Δ*) also improved the yield of recombinant Fps1 compared to wild-type. Expression of the eukaryotic transcriptional activator *GCN4* was increased in *spt3Δ*, *srb5Δ*, *gcn5Δ* and yTHC*BMS1* (supplemented with 0.5 μg/mL doxycycline); these four strains also exhibited constitutive phosphorylation of the eukaryotic initiation factor, eIF2α. Both responses are indicative of a constitutively-stressed phenotype. Investigation of the 5′UTR of *FPS1* in the expression construct revealed two untranslated ORFs (uORF1 and uORF2) upstream of the primary ORF. Deletion of either uORF1 or uORF1 and uORF2 further improved recombinant yields in our four strains; the highest yields of the uORF deletions were obtained from wild-type cells. Frame-shifting the stop codon of the native uORF (uORF2) so that it extended into the *FPS1* ORF did not substantially alter Fps1 yields in *spt3Δ* or wild-type cells, suggesting that high-yielding strains are able to bypass 5′uORFs in the *FPS1* gene via leaky scanning, which is a known stress-response mechanism. Yields of recombinant A_2a_R, GFP and horseradish peroxidase could be improved in one or more of the yeast strains suggesting that a stressed phenotype may also be important in high-yielding cell factories.

**Conclusions:**

Regulation of Fps1 levels in yeast by translational control may be functionally important; the presence of a native uORF (uORF2) may be required to maintain low levels of Fps1 under normal conditions, but higher levels as part of a stress response. Constitutively-stressed yeast strains may be useful high-yielding microbial cell factories for recombinant protein production.

**Electronic supplementary material:**

The online version of this article (doi:10.1186/s12934-017-0656-2) contains supplementary material, which is available to authorized users.

## Background

In recent years, our understanding of how to synthesize recombinant membrane proteins in microbes has benefitted from insights into the underlying molecular mechanisms [1] in a range of host cells including *Escherichia coli* [2, 3], *Lactococcus lactis* [4], *Saccharomyces cerevisiae* [5–8] and *Pichia pastoris* [9, 10]. Despite these advances, several membrane proteins still remain intractable to recombinant production [5]. The aquaporin Fps1, which has a central role in yeast mating [11] as well as the cellular response to stresses including osmotic, acetic acid and toxic metalloid stress [12, 13], is one such protein.

In order to obtain sufficient yields of Fps1 for further study, we previously used comparative transcriptome analysis to identify genes that were up- or down-regulated in *S. cerevisiae* when recombinant Fps1 was produced under a range of different culture conditions [8]. We noted that relatively high-yielding conditions were associated with the down-regulation of *SPT3*, *SRB5* or *GCN5* or the up-regulation of *BMS1* [8]. We used these findings to select yeast strains specifically for the production of recombinant Fps1: we chose three strains in which each of the down-regulated genes was singly deleted and one in which the upregulated gene was over-expressed. When cultured in stirred-tank bioreactors, Fps1 yields from strains *spt3Δ, srb5Δ, gcn5Δ* and yTHC*BMS1* (in the latter strain, the promoter is tuned by addition of 0.5 μg/mL doxycycline) were 10–80 times higher than yields from wild-type cells [6]. We also demonstrated more modest yield improvements when other target proteins were produced in yTHC*BMS1* (10 μg/mL doxycycline): yields of the G protein-coupled receptor, adenosine receptor 2a (A_2a_R) and soluble green fluorescent protein (GFP) [6] were doubled compared to controls in some cases.

We noted that all four strains had elevated levels of *BMS1* transcript compared to wild-type [6]; Bms1 is involved in ribosome biogenesis [14], suggesting that post-transcriptional mechanisms might be responsible for these high-yielding phenotypes. Notably, the expression plasmid used to produce Fps1 contained 249 base pairs (bp) of 5′ untranslated region (UTR) in addition to the primary *FPS1* open reading frame (ORF) meaning that translational control might have a role in defining the final yield of Fps1 in our four strains. The aim of this study was therefore to investigate the translational mechanisms in our four high-yielding strains (*spt3Δ, srb5Δ, gcn5Δ* and yTHC*BMS1*) in order to understand the molecular determinants underpinning the expression of *FPS1*.

## Results and discussion

### High-yielding yeast strains exhibit blocks in translation initiation

**Fig. 1:**
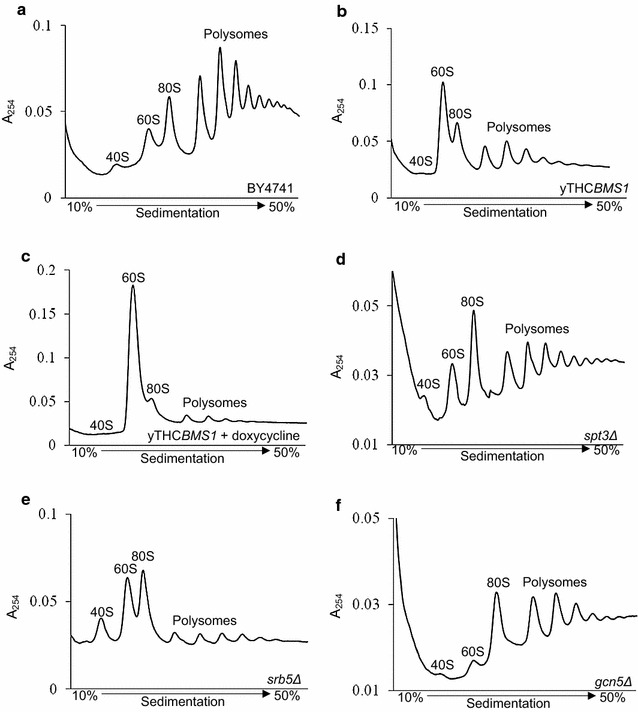
Some high-yielding Fps1 strains exhibit initiation blocks. Representative polysome profiles for the high-yielding strains and the wild-type control. Cells were cultured in YPD medium and harvested at A_600_ ~ 1. Values in *square brackets* are the monosome/polysome ratio with the standard error of the mean shown in parentheses, as follows: **a** BY4741 wild-type control [0.15 (n = 3, 0.01)], **b** yTHC*BMS1* [0.24 (n = 3, 0.03)], **c** yTHC*BMS1* supplemented with 0.5 μg/mL doxycycline [0.43 (n = 3, 0.14)], **d** *spt3Δ* [0.18(n = 3, 0.03)], **e** *srb5Δ* [0.39 (n = 3, 0.07)], **f** *gcn5Δ* [0.16 (n = 2, 0.02)]

On the basis that our four strains had elevated levels of *BMS1* transcript compared to wild-type [6] and that Bms1 is involved in ribosome biogenesis [14], polysome profiles were generated from shake-flask cultures. The initiation phase of translation is the rate-limiting step of protein synthesis [15, 16]. Using polysome profiling, it is possible to measure the numbers of ribosomes bound to a cell’s mRNA pool under defined conditions. Differences in the ratio of bound polyribosomes (“polysomes”; two or more ribosomes) to bound single ribosomes (“monosomes”) are indicative of alterations in translation. A translation initiation block is typically associated with the majority of mRNAs being monosomal; in contrast, highly-translated mRNAs are polysomal. Figure 1a shows a typical polysome profile for the parental wild-type strain BY4741, which was not altered on supplementation with 0.5 μg/mL doxycycline (profile not shown; Table 1). The polysome profile of yTHC*BMS1* (with no doxycycline supplementation; Fig. 1b; Table 1) suggests a block in translation initiation, which is defined as an increase in the ratio of the 80S monosome peak area to the area of the polysome peaks, compared to the wild-type ratio (Table 1). Cells subjected to stresses such as heat shock or nutrient starvation typically exhibit initiation blocks; this is consistent with the down-regulation of translation in order to conserve energy, protect the proteome from damage and elicit alterations in gene expression to promote protection [15, 16]. Supplementation of yTHC*BMS1* with 0.5 μg/mL doxycycline increased the severity of the initiation block and increased Fps1 yield (Fig. 1c; Table 1). The decrease in the 40S ribosomal subunit peak and relative increase in the 60S peak is probably responsible for the initiation block in this case, since fewer functional ribosomes would be able to form. Strains *spt3Δ* and *srb5Δ* also exhibited a clear initiation block, but not on account of an altered ribosomal subunit ratio as seen for yTHC*BMS1* with 0.5 μg/mL doxycycline; for these two strains the subunit ratio is not perturbed (Fig. 1d, e; Table 1). The polysome profile of *gcn5Δ* resembled that of cells with a mild initiation block because of the relatively high 80S peak compared to the wild-type control (Fig. 1f; Table 1) [18]. The deletion strains have not previously been reported to have altered translation capacities; *SPT3*, *GCN5* and *SRB5* are global regulators of transcription [19, 20].

**Table 1:** Characteristics of high-yielding yeast strains

Yeast strain	Fps1 yield (µg/L) *FPS1*-HA_3_	5′∆1-43- *FPS1*-HA_3_	5′∆1-215- *FPS1*-HA_3_	M:P ratio	*GCN4* expression	Constitutive phosphorylation of eIF2α
Wild-type (BY4741)	0.02 (0.01)	0.21 (0.06)	12.14 (1.68)	0.15 (0.01)	1.0 (0.4)	N
Wild-type + 0.5 µg/mL doxycycline	0.02 (0.01)	–	–	0.15 (0.01)	–	–
yTHC*BMS1*	0.12 (0.02)	–	–	0.24 (0.03)	2.7 (0.1)	–
yTHC*BMS1* + 0.5 µg/mL doxycycline	0.73 (0.15)	2.39 (0.54)	8.00 (2.50)	0.43 (0.14)	2.2 (0.3)	Y
*srb5Δ*	0.13 (0.03)	–	–	0.39 (0.07)	1.5 (0.1)	Y
*gcn5Δ*	0.23 (0.04)	–	–	0.16 (0.02)	6.0 (0.5)	Y
*spt3Δ*	0.91 (0.07)	1.36 (0.28)	4.70 (1.11)	0.18 (0.03)	4.1 (0.4)	Y

Yeast cells were transformed with a plasmid expressing *FPS1*-HA_3_, 5′∆1-43-*FPS1*-HA_3_ or 5′∆1-215-*FPS1*-HA_3_, as indicated. Single transformants were cultured in shake flasks in 2× CBS lacking histidine to maintain the plasmid. Cells were harvested just before the diauxic shift by monitoring residual glucose concentration (cultures had a typical biomass yield of 0.9 g/L; A_600_ ~ 4). Fps1 yields (µg/L) were determined with reference to a BSA standard curve [38]; a yield of 12.14 µg/L may also be expressed as 13.49 µg/g dry cell weight. The ratio of monosome to polysome peaks (M:P) was calculated from polysome profiles obtained by fractionation on a 10–50% sucrose gradient. To determine *GCN4* expression, yeast cells were transformed with plasmid B1805 [31] or, for yTHC*BMS1*, B1805-*HIS*. Yeast cells expressing *GCN4*-*LacZ* were cultured in shake flasks, harvested at A_600_ ~ 1 and β-galactosidase levels were determined using ONPG. β-Galactosidase levels are expressed relative to wild-type control cells. For analysis of eIF2α phosphorylation, cells were cultured in the absence (amino-acid-starved cells) or presence (control cells) of amino acids and cells lysates were analysed by immunoblot using anti-eIF2α and anti-phospho-eIF2α antibodies as previously described [39]. All data shown are the mean of biologically-independent, triplicate determinations; where relevant, the standard error of the mean (SEM) is shown in parentheses

*Y* indicates yes, *N* indicates no and – indicates that the experiment was not performed

**Fig. 2:**
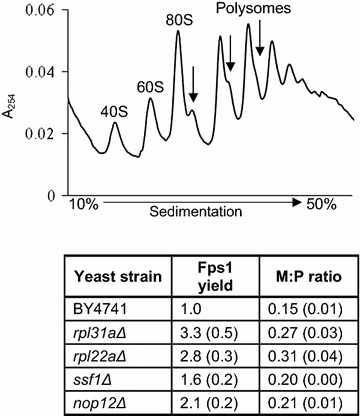
Four additional strains with translation initiation blocks are high-yielding for Fps1. The ribosomal biogenesis deletion mutants, *ssf1Δ*, *nop12Δ*, *rpl31aΔ* and *rpl22aΔ* have a decrease in the levels of 60S ribosomal subunit [21, 22]. The *upper panel* shows a representative polysome profile containing halfmers after each monosome and polysome peak for the strain *nop12Δ*. Halfmers (indicated with *arrows*) occur when the PIC binds to mRNA in the absence of the 60S subunit; the PIC then scans through the start codon [23]. The monosome/polysome ratio for each strain is tabulated in the lower panel. The four mutant strains were then transformed with the vector pYX222-*FPS1*-HA_3_ and Fps1 yields analysed at A_600_ ~ 1 by immunoblot; values are reported relative to *FPS1*-HA_3_ expressed in wild-type cells. Experiments were performed in biological triplicates; where relevant, the standard error of the mean (SEM) is shown in parentheses

To test the hypothesis that high-yielding strains may exhibit a block in translation initiation, four additional strains with altered translational profiles were selected. The ribosomal biogenesis deletion mutants, *ssf1Δ*, *nop12Δ*, *rpl31aΔ* and *rpl22aΔ* have a decrease in the levels of 60S ribosomal subunit [21, 22]. Figure 2 shows a representative polysome profile containing halfmers after each monosome and polysome peak. Halfmers occur when the pre-initiation complex (PIC) binds to mRNA in the absence of the 60S subunit; the PIC then scans through the start codon in a mechanism known as leaky scanning [23]. When these four additional mutant strains were transformed with the vector pYX222-*FPS1*-HA_3_ and Fps1 production was analysed in shake flask cultures, Fps1 yields were higher than in wild-type cells (Fig. 2). These data suggest that yeast cells that are high-yielding for Fps1 may also exhibit an initiation block, but that the extent of the initiation block and the Fps1 yield are not correlated. We therefore continued to analyze the translational capacity of *spt3Δ, srb5Δ, gcn5Δ* and yTHC*BMS1* (supplemented with 0.5 μg/mL doxycycline).

### Phosphorylation of eIF2α is constitutive in high-yielding yeast strains

**Fig. 3:**
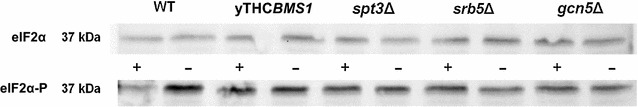
eIF2α phosphorylation is constitutive in high-yielding Fps1 strains. Phosphorylation of eIF2α, as detected by immunoblot, is shown in amino-acid-starved and control cells: (+) represents cells cultured in growth medium supplemented with amino acids (control cells) whilst (−) represents cells cultured in growth medium lacking amino acids for 10 min (starved cells). yTHC*BMS1* cultures were also supplemented with 0.5 μg/mL doxycycline. The *upper panel* (37 kDa) was detected with an anti-eIF2α antibody, whilst the* lower panel* (37 kDa) was detected with an anti-phospho-eIF2α antibody

Phosphorylation of the eukaryotic initiation factor subunit eIF2α was investigated. When eIF2α is phosphorylated, during times of stress, it prevents the exchange of eIF2•GDP for eIF2•GTP, decreasing the level of ternary complex (TC), which in turn reduces the pool of PIC and may cause an initiation block. Figure 3 shows that eIF2α is constitutively phosphorylated in all four high-yielding strains; the upper, control panel was detected with an anti-eIF2α antibody, whilst the lower panel was detected with an anti-phospho-eIF2α antibody. The data in the lower panel suggest that all four of our strains are constitutively stressed compared to wild-type cells.

### *GCN4* expression is increased in high-yielding yeast strains

**Fig. 4:**
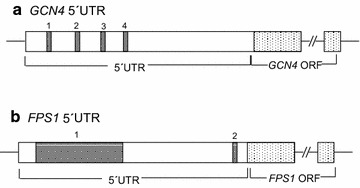
Schematic representations of the uORFs in the 5′UTRs of *GCN4* and *FPS1* transcripts. *Dark grey*, numbered blocks represent uORFs in the 5´UTRs of both transcripts; *dotted blocks* represent the primary ORFs. **a** The unique 5´UTR of *GCN4* contains four uORFs that prevent translation of the *GCN4* ORF when levels of TC are high under normal conditions. As a result, the scanning 40S does not reach the *GCN4* ORF, instead translating the uORFs [24]. **b** The 5′UTR of *FPS1* defined by the vector pYX222-*FPS1*-HA_3_ contains two uORFs; the effects of these uORFs were studied by generating pYX222-5′∆1-43-*FPS1*-HA_3_ and pYX222-5′∆1-215-*FPS1*-HA_3_, which contain only the native uORF (uORF2) or no uORFs, respectively

It is well known that in yeast, cellular stress is associated with increased expression of the eukaryotic transcriptional activator gene, *GCN4*. Translation of *GCN4* is regulated by four upstream open reading frames (uORF; Fig. 4a). At low levels of eIF2α phosphorylation, such as those found under normal conditions, the TC is abundant. It has been demonstrated experimentally that ribosomes that initiate at uORF1 (Fig. 4a) can also continue scanning; the AT-rich sequences around the stop codon of uORF1 promote ribosome retention, enabling re-initiation of translation at uORF2, uORF3 or uORF4. However, ribosomes that terminate at uORFs 2–4 do not resume scanning because GC-rich sequences promote ribosome disassociation, decreasing the probability of the *GCN4* ORF being translated [24]. During stress conditions, increased levels of eIF2α phosphorylation (e.g. Fig. 3) reduce the abundance of the TC so that re-initiation at uORFs 2-4 becomes even less frequent, allowing scanning ribosomes to reach the *GCN4* ORF and translate it.

*GCN4* expression (assayed in our four strains using a β-galactosidase reporter), was shown to be increased in all strains relative to wild-type cells (Table 1). Increased Gcn4 levels in *srb5Δ, gcn5Δ, spt3Δ* and yTHC*BMS1* (supplemented with 0.5 μg/mL doxycycline) may be the result of initiation blocks due to the eIF2α phosphorylation observed in these strains. For the ribosomal biogenesis and ribosomal protein mutant strains, a decrease in the level of the 60S subunit enables the PIC to scan through the uORFs in the *GCN4* transcript; it has already been shown that ribosomal mutants have increased levels of Gcn4 [21] on account of leaky scanning by 40S subunits [17] through uORFs 1-4.

### High-yielding yeast strains can bypass uORFs in the *FPS1* transcript

On account of a possible association between increased Fps1 yields and constitutive stress phenotypes, the sequence of pYX222-*FPS1*-HA_3_ was analysed for the presence of start codons upstream of the *FPS1* ORF and downstream of the *TPI1* promoter. To define the 5′UTR region, transcriptional start site information for *TPI1* was obtained from the literature [25]. Additional file 1: Figure S2; Fig. 4b and Table 2 illustrate the 2 uORFs in the 5′UTR of the *FPS1* expression construct. The first *FPS1* uORF (uORF1) is 34 codons in length and 154 nucleotides upstream of the *FPS1* ORF. The second uORF (uORF2) is 3 codons in length, similar to those found in *GCN4*, but 5 nucleotides from the *FPS1* ORF; uORF2 occurs natively in the yeast genome in the 5′UTR of *FPS1* [25].

**Table 2:** Sequences upstream of the *FPS1* ORF in the four expression constructs used in this study

Vector name	Sequence (sense strand; 5′ to 3′)	Reference
pYX222-*FPS1*-HA_3_	…TAACTACAAAAAACACATACAGGAATTCACCATGGATCTCATAGTGAGAAGGCGCAATTCAGTAGTTAAAAGCGGGGAACAGTGTGAATCCGGAGACGGCAAGATTGCCCGGCCCTTTTTGCGGAAAAGATAAAACAAGATATATTGCACTTTTTCCACCAAGAAAAACAGGAAGTGGATTAAAAAATCAACAAAGTATAACGCCTATTGTCCCAATAAGCGTCGGTTGTTCTTCTTTATTATTTTACCAAGTACGCTCGAGGGTACATTCTAATGCATTAAAAGAC**ATGAGTAATCCTCAAAAAGCTCTAAACGACTT …**	[8]; Additional file 1: Figure S2
pYX222-5′Δ1-43-*FPS1*-HA_3_	…TAACTACAAAAAACACATACAGGAATTCGCGGGGAACAGTGTGAATCCGGAGACGGCAAGATTGCCCGGCCCTTTTTGCGGAAAAGATAAAACAAGATATATTGCACTTTTTCCACCAAGAAAAACAGGAAGTGGATTAAAAAATCAACAAAGTATAACGCCTATTGTCCCAATAAGCGTCGGTTGTTCTTCTTTATTATTTTACCAAGTACGCTCGAGGGTACATTCTAATGCATTAAAAGAC**ATGAGTAATCCTCAAAAAGCTCTAAACGACTT …**	This study; Additional file 1: Figure S3
pYX222-5′∆1-43-uORF2-stop-removed-*FPS1*-HA_3_		This study; Additional file 1: FigureS4
pYX222-5′Δ1-215-*FPS1*-HA_3_	…TAACTACAAAAAACACATACAGGAATTCC**ATGAGTAATCCTCAAAAAGCTCTAAACGACTT …**	This study; Additional file 1: Figure S5

Four vectors were used in this study; their construction is described in the “Methods” section and their sequences are given as Additional file 1, as indicated. The sequences directly upstream of the *FPS1* ORF (in bold), are shown. The uORFs are underlined; uORF1 is 34 codons long; uORF2, which is found natively in the genomic *FPS1* sequence, is 3 codons long. The two additional cytosines in pYX222-5′∆1-43-uORF2-stop-removed-*FPS1*-HA_3_ are italicised and the consequent extension of uORF2 is highlighted in grey

Since translation can be controlled at ORFs by uORFs [24, 26], we reasoned that the presence of *FPS1* uORFs might limit recombinant Fps1 yields. Two new vectors were therefore created (Table 2), one to delete uORF1 (pYX222-5′∆1-43-*FPS1*-HA_3_) and the second to delete both uORFs (pYX222-5′∆1-215-*FPS1*-HA_3_). To investigate the impact of deleting these uORFs, Fps1 yields were analysed in wild-type cells, the yTHC*BMS1* strain (supplemented with 0.5 μg/mL doxycycline) and the *spt3Δ* strain, each transformed with pYX222-*FPS1*-HA_3_, pYX222-5′∆1-43-*FPS1*-HA_3_ or pYX222-5′∆1-215-*FPS1*-HA_3_. Table 1 shows Fps1 yields compared with wild-type cells transformed with pYX222-*FPS1*-HA_3_ (the control condition). When uORF1 was deleted from the *FPS1* transcript, the yield of Fps1 increased 11 times in wild-type yeast relative to the control; when both uORFs were removed, the yield increased 607 times relative to the control. When two uORFs were present in the *FPS1* transcript, yTHC*BMS1* supplemented with 0.5 μg/mL doxycycline increased Fps1 yield 37 times relative to the control. Deleting uORF1 increased Fps1 yields 120 times; deleting both increased yields 400 times. When both uORFs are present, yield improvement was 46 times higher than control for *spt3Δ* cells, 68 times higher when the first uORF was deleted and 235 times higher when both uORFs were deleted. Notably, when both uORFs were deleted, wild-type cells produced the highest yields of Fps1 compared to any other combination of strain and vector. Overall, these data suggest that (a) in wild-type cells, the expression of *FPS1* is less than 2% of its maximum value in the presence of native uORF2 (12.14 µg/L or 13.49 µg/g dry cell weight, Table 1) and (b) that our four high-yielding, bespoke strains can circumvent both uORFs (Table 1; Figs. 1, 3).

### High-yielding yeast strains bypass uORFs by leaky scanning to produce Fps1

Translational control by uORFs is known to occur through two main mechanisms: re-initiation or leaky scanning [27]. As exemplified by the case of *GCN4*, re-initiation occurs when, after completing translation of an uORF, the 40S ribosomal subunit does not dissociate from the mRNA and can therefore initiate translation at the start codon of a downstream ORF [27]. Leaky scanning occurs when a proportion of the scanning PIC initiates translation of the uORF, while the rest scans along the transcript and initiates translation at the start codon of the downstream ORF [27]. Both re-initiation [27] and leaky scanning [28, 29] of uORFs have been shown to occur during times of stress.

To identify the mechanism responsible for translational control by uORF2, the uORF2 stop codon was shifted out of frame with its start codon in pYX222-5′∆1-43-*FPS1*-HA_3_ to generate pYX222-5′∆1-43-uORF-stop-removed-*FPS1*-HA_3_ (Table 2). In this new vector, uORF2 now extends 5 nucleotides into (and is out of frame with) the *FPS1* ORF (Table 2). If Fps1 were produced from this new vector via re-initiation, Fps1 yields should decrease because translation of uORF2 would not be completed before the start codon of the downstream *FPS1* ORF. However, if the mechanism were leaky scanning, Fps1 levels should be similar to, or even higher than, those produced from pYX222-5′∆1-43-*FPS1*-HA_3_, as recently found when investigating the translational control of the transcriptional regulator, CHOP [28].

Wild-type and *spt3Δ* cells were transformed with pYX222-5′∆1-43-*FPS1*-HA_3_ (control vector) and pYX222-5′∆1-43-uORF-stop-removed-*FPS1*-HA_3_ and cultured in 2× CBS lacking histidine. When Fps1 yields were determined by densitometry of immunoblot signals from wild-type and *spt3Δ* cultures expressing pYX222-5′∆1-43-uORF-stop-removed-*FPS1*-HA_3_, they were approximately double (1.99-fold; SEM 0.34 and 1.89-fold; SEM 0.45, respectively) that of control cultures expressing pYX222-5′∆1-43-*FPS1*-HA_3_. This increase in yield is more consistent with a leaky scanning mechanism than re-initiation. Furthermore, only 5 nucleotides separate uORF2 from the *FPS1* ORF; Kozack previously demonstrated that re-initiation is inefficient if fewer than 79 nucleotides separate uORF and ORF (in that study the ORF encoded preproinsulin) [30]. In our strains it is possible that leaky scanning may be a consequence of defects such as subunit joining [23] or start codon recognition [31]. Others have also demonstrated that leaky scanning of uORFs occurs during times of stress, mediated by eIF1 phosphorylation and resulting in the translation of a subset of mRNAs important for survival or apoptosis [28, 29]. Notably yields of recombinant GFP, horseradish peroxidase (HRP) and A_2a_R can be improved in the high-yielding yeast strains suggesting more generic benefits (Table 3).

**Table 3:** Yields of recombinant GFP, HRP and A_2a_R can be improved in the high-yielding yeast strains

Yeast strain	GFP yield (AU)	HRP yield (U/mL)	A_2a_R yield (pmol/mg)
Wild-type (BY4741)	1.0 (0.2)	5.9 (0.7)	7.4 (0.7)
yTHCBMS1	0.9 (0.2)	28.1 (4.0)	–
yTHCBMS1 + 0.5 µg/mL doxycycline	1.3 (0.1)	–	9.6 (1.3)
yTHCBMS1 + 10 µg/mL doxycycline	4.7 (0.8)	–	16.1 (1.7)
*srb5Δ*	1.6 (0.3)	–	–
*gcn5Δ*	1.5 (0.4)	–	–
*spt3Δ*	0.8 (0.1)	16.0 (2.0)	–

Yeast cells were transformed with a plasmid expressing green fluorescent protein (GFP; Additional file 1: Figure S7), horseradish peroxidase (HRP; Additional file 1: Figure S8) or the adenosine A_2a_ receptor (A_2a_R; Additional file 1: Figure S9). Single transformants were cultured in shake flasks in 2× CBS lacking histidine (to maintain the plasmid). GFP yields were determined by fluorimetry of the culture supernatant as previously described [6], but with cells harvested from 50 mL cultures at A_600_ = 4. They are reported as arbitrary units (AU) normalised to the value for wild-type cultures. HRP yields are reported in U/mL 24 h post-induction, where one unit of HRP decomposes 1 µmol of peroxide/min at 25 °C. Data for A_2a_R were published previously [6] and are reported as B_max_ values (pmol/mg membrane). All data shown are the mean of biologically-independent, triplicate determinations; the standard error of the mean (SEM) is shown in parentheses

### Implications for the regulation of Fps1

**Fig. 5:**
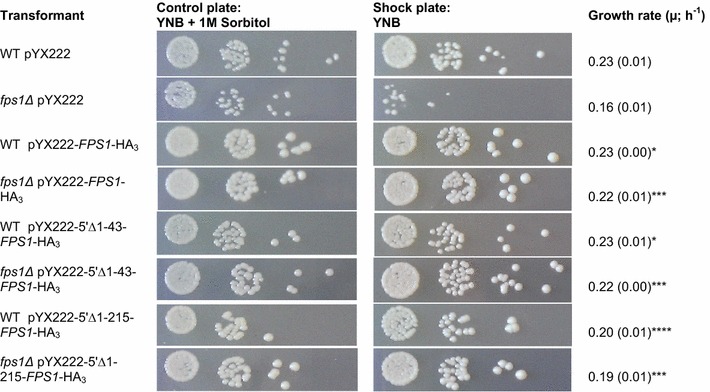
Fps1 produced from plasmids containing 0, 1 or 2 uORFs rescues the osmosensitive phenotype of *fps1Δ* cells. Yeast cells (BY4741 (WT) or *fps1Δ*) were transformed with pYX222 (empty vector control), pYX222-*FPS1*-HA_3_ (containing 2 uORFs), pYX222-5′Δ1-43-*FPS1*-HA_3_ (containing 1 uORF) or pYX222-5′Δ1-215-*FPS1*-HA_3_ (containing 0 uORFs) and grown in 2× CBS (without histidine) supplemented with 1 M sorbitol. Cells were spotted (10 μL spots) in 10-fold serial dilutions onto YNB agar plates with or without 1 M sorbitol. A representative image is shown from three biologically independent determinations. Growth rates were calculated in 200 μL 2× CBS in a 96 well plate format (n = 6). Standard error of the mean (SEM) is given in parentheses. A one-way ANOVA followed by a Dunnett’s multiple comparison test was used to analyze the growth rate data where *P ≤ 0.05, **P ≤ 0.01, ***P ≤ 0.001 and ****P ≤ 0.0001

Fps1 facilitates glycerol efflux from yeast cells under conditions of hypo-osmotic stress; the *fps1Δ* strain is sensitive to osmotic down-shifts [32, 33]. The regulation of Fps1 by gating is known to be mediated by its amino- and carboxy-terminal regions [34, 35], but it is not known whether other mechanisms are also involved. In order to investigate a physiological role for uORF2, we aligned the 5′UTR of *FPS1*, using the alignment facility of the *Saccharomyces* Genome Database (SGD; www.yeastgenome.org/cgi-bin/FUNGI/ShowAlign), which revealed that the yeasts *S. mikatae* and *S. paradoxus* also have a similar uORF in their *FPS1* gene. We next confirmed that the Fps1 produced from our expression plasmids (Table 2) was functional. Figure 5 shows that Fps1 produced from all three vectors (with 0, 1 or 2 uORFs; Table 2) rescues the well-established osmosensitive phenotype of *fps1Δ* cells [32, 33], and that sufficient Fps1 is produced in the presence of the uORF(s) for this purpose. The specific growth rates of the transformants were calculated from corresponding liquid cultures and were found to be consistent with the qualitative serial spot assays (Fig. 5). Notably, the transformants (WT and *fps1∆*) with no uORF grew marginally more slowly than those with one or two uORFs suggesting that unregulated translation of *FPS1* may be detrimental to yeast cells (Fig. 5). This observation was also seen in cultures of yTHC*BMS1* and *spt3∆*. The growth rate decreased by 12.5 and 6.9% in yTHC*BMS1* and *spt3∆* respectively when there were no uORFs in the *FPS1* transcript compared to the presence of two uORFs (Fig. 5).

The translational state of yeast cells during hypo-osmotic shock was analysed because Fps1 is involved in glycerol efflux under these conditions. The yields of Fps1 produced in wild-type cells from the vector pYX222-5′∆1-43-*FPS1*-HA_3_ were analysed after 15, 30, 60 and 90 min of hypo-osmotic shock. Notably, the Fps1 yield and the translational state of the cells (as determined by polysome analysis) were indistinguishable from control (unshocked) cells (data not shown), suggesting that the primary functional role of Fps1 as a glycerol efflux channel following osmotic down-shift is not translationally controlled by uORF2.

Translation initiation blocks are observed in yeast following hyper-osmotic shock [36], which we confirmed for wild-type strain BY4741 (Table 4); the block was immediate and alleviated after 90 min, as previously described [36]. In the presence of native uORF2, Fps1 yields increased ~threefold following hyperosmotic shock compared to control, unshocked cells (Table 4). The yield decreased as the initiation block was alleviated (Table 4). When both uORFs were removed (using pYX222-5′∆1-215-*FPS1*-HA_3_) there was no increase in Fps1 production following hyperosmotic shock (data not shown). A physiological role for uORF2 may therefore be to control the levels of Fps1 (to 2% of its maximum possible value) by keeping it low during normal conditions (Table 1), but increasing it as part of a stress response (Table 4). Notably, high levels of Fps1 may be detrimental to the cell under normal conditions because Fps1 has been shown to be involved in the uptake of cytotoxic compounds such as arsenite and acetic acid that lead to enzyme inhibition or apoptosis, respectively [12, 37].

**Table 4:** Fps1 yields following hyperosmotic shock

Osmotic shock	Time post-shock (min)	Fps1 yield relative to control	M:P ratio
No shock (2× CBS)	15	1.00 (0.61)	0.24 (0.00)
	30	0.59 (0.17)	0.22 (0.03)
	60	0.90 (0.48)	0.14 (0.01)
	90	1.21 (0.63)	0.10 (0.00)
Hyperosmotic shock (2× CBS, 0.8 M NaCl)	15	3.03 (0.21)*	1.08 (0.14)**
	30	2.62 (0.83)	1.24 (0.16)***
	60	1.73 (0.29)	0.29 (0.03)
	90	2.08 (0.58)	0.21 (0.02)*

Wild-type yeast cells were transformed with a plasmid expressing 5′∆1-43-*FPS1*-HA_3_; uORF2 was therefore present (Table 1). Single transformants were cultured in shake flasks in 2 × CBS lacking histidine (to maintain the plasmid). At A_600_ ~ 4, cultures were divided into two, harvested by centrifugation and the pellets suspended in either 2× CBS (no shock) or 2× CBS supplemented with 0.8 M NaCl (hyperosmotic shock). Fps1 yields were determined by immunoblot and analyzed using ImageJ [6] after 15, 30, 60 and 90 min; values are reported relative to control conditions (wild-type cells after 15 min in 2× CBS). All data shown are the mean of biologically-independent, triplicate determinations; where relevant, the standard error of the mean (SEM) is shown in parentheses. A two-tailed paired *t* test was used to compare Fps1 yields or polysome ratios of shocked cells with the corresponding values for control cells at the equivalent time point

* P ≤ 0.05; ** P ≤ 0.01; *** P ≤ 0.001

## Conclusions

In common with many membrane proteins, the recombinant production of Fps1 (a facilitator for glycerol efflux from yeast cells [32, 33]) has proved challenging. We previously identified four strains of *S. cerevisiae* (*spt3Δ, srb5Δ, gcn5Δ* and yTHC*BMS1* supplemented with 0.5 μg/mL doxycycline) that specifically produced Fps1 in sufficient yield for further study [6]. However, the mechanism underpinning their high-yielding phenotype was unknown. A significant finding emerging from this work is that strains that are high-yielding for Fps1 may have translation initiation blocks (Fig. 1), exhibit constitutive phosphorylation of eIF2α (Fig. 3) and have increased expression of *GCN4* (Table 1). Our data suggest that the strains can bypass, via a leaky scanning mechanism, a native uORF that we identified in the *FPS1* gene (see http://doi.org/10.17036/researchdata.aston.ac.uk.00000176; Additional file 1: Figures S2, S4). While the strains were selected for improved expression of Fps1 and so give the best yields for that target, a second significant finding is that these strains can also be used to increase the yields of other heterologous proteins (Table 3).

A third significant finding of this work is the identification of a previously-unknown regulatory element in the *FPS1* gene. The discovery of a native uORF suggests that Fps1 may be regulated translationally. It is known that regulation of Fps1 function occurs through gating by its amino- and carboxy-terminal regions and that unregulated Fps1 renders yeast cells sensitive to osmotic [34, 35], arsenite [37] and acetic acid [12] stresses. A physiological role for uORF2 may therefore be to control the levels of Fps1 (to a little as 2% of its maximum possible value) during normal conditions (Table 1), but increase it as part of a stress response (Table 3).

## Methods

### Strains and vectors

The haploid *S. cerevisiae* BY4741 strain (MATα, *ura3Δ0, leu2Δ0, met15Δ0, his3Δ1*) is the parental strain of the deletion mutants*, gcn5∆, spt3∆*, *srb5∆*, *rpl22a∆, rpl31a∆, ssf1∆* and *nop12∆* (from the EUROSCARF collection) and the yTHC*BMS1* strain (Open Biosystems) used in this study, and as such provided the wild-type control. Expression of *BMS1* in the yTHC*BMS1* strain is under the control of a doxycycline-repressible promoter; we have previously shown that supplementing the strain with 0.5 μg/mL doxycycline gives maximum Fps1 yields [6]. pYX222-*FPS1*-HA_3_ (found to contain two uORFs; uORF1 and uORF2) was described previously [8]. pYX222-5′Δ1-43-*FPS1*-HA_3_ (containing only the native uORF, uORF2; Table 1) was made by digesting pYX222 [8] with *Eco*RI and *Hind*III to remove the Kozak sequence and inserting a *FPS1* fragment with a truncated 5′UTR starting 216 bp upstream of the *FPS1* ORF. pYX222-5′Δ1-215-*FPS1*-HA_3_ (containing no uORFs; Table 1) contained a *FPS1* fragment with 1 bp upstream of the *FPS1* ORF. pYX222-5′∆1-43-uORF-stop-removed-*FPS1*-HA_3_ (Table 1) was made by inserting two cytosines before the stop codon of uORF2 to cause a frame shift (forward primer: CCCCCCGAATTCATGCACCTTAAAAGACATG). The vector B1805 (the kind gift to Mark P. Ashe of Alan Hinnebusch) contains the *GCN4* promoter and complete 5´UTR; *LacZ* is inserted into the *Bam*HI site of the *GCN4* ORF [31]. For experiments requiring nutrient selection on histidine-deficient medium, B1805-*HIS* was used in which the *URA3* gene was replaced with the *HIS3* gene by restriction digest with *Xma*I and *Kas*I. Yeast cells were transformed using the lithium acetate method [8].

### Culture conditions

Yeast strains were cultured in shake flasks in either YPD or 2× CBS. YPD contains 1% yeast extract, 2% bacto peptone and 2% glucose. 1 L of 2× CBS is composed of 10 g/L ammonium sulfate, 6 g/L potassium dihydrogen phosphate, 1 g/L magnesium sulfate heptahydrate supplemented with 2% glucose, 2× DO solution, 100 mM MES, 2 mL/L each of trace element solution and vitamin stock solution. 10 × DO solution (/L) was composed of 200 mg adenine hemisulfate, 200 mg l-arginine hydrochloride, 300 mg l-isoleucine, 1000 mg l-leucine, 300 mg l-lysine hydrochloride, 200 mg l-methionine, 500 mg l-phenylalanine, 2000 mg l-threonine, 200 mg l-tryptophan, 300 mg l-tyrosine, 200 mg uracil, 1500 mg l-valine. 250 mL trace element solution was composed of 3.75 g EDTA, 1.125 g zinc sulfate heptahydrate, 0.25 g magnesium chloride tetrahydrate, 0.075 g cobalt (II) chloride hexahydrate, 0.075 g copper (II) sulfate pentahydrate, 0.1 g sodium molybdenum dehydrate, 1.125 g calcium chloride dehydrate, 0.75 g iron (II) sulfate heptahydrate, 0.25 g boric acid and 0.025 g potassium iodide. 250 mL vitamin stock solution was composed of 0.0125 g biotin, 0.25 g calcium-D-pantothenate, 0.25 g nicotinic acid, 6.25 g myo-inositol, 0.25 g thiamine hydrochloride, 0.25 g pyridoxine hydrochloride and 0.05 g d-amino benzoic acid; pH was maintained at 6.5.

### Cell membrane preparation and Fps1 yield analysis

Yeast strains were cultured in shake flasks in 2× CBS. Cells were harvested prior to the diauxic shift as determined by residual glucose concentration readings using a Roche Accu-Chek Active diabetes monitor. Cells were pelleted at 5000×*g*, 4 °C for 3 min, the supernatant discarded and the pellet frozen at −20 °C until required. The pellet was washed once, suspended in 1 mL breaking buffer (50 mM Na_2_HPO_4_, 50 mM NaH_2_PO_4_, 2 mM EDTA pH 7.4, 100 mM NaCl, 5% glycerol) and transferred to a breaking tube containing 1 mL glass beads. Protease inhibitor cocktail IV (Calbiochem) was added at a dilution factor of 1:500 (typically 2 μL). Cells were broken in a cold TissueLyser (Qiagen) at 50 Hz for 10 min. The cell lysate was cleared by centrifugation at 25,000×*g* for 15 min. Membranes were harvested by centrifugation at 190,000×*g* for 1 h. The membrane pellet was suspended in 100 μL Buffer A (20 mM HEPES, 50 mM NaCl, 10% glycerol, pH 7) and stored at −20 °C until required. Total protein concentration was determined by BCA assay. Fps1 yield was determined by a densitometric analysis of silver-stained SDS-PAGE gels as previously described [38]. The Fps1 band in the membrane fraction was compared with a series of known amounts of bovine serum albumin (0.75, 0.5, 0.25, 0.125, 0.05, 0.025 and 0.01 µg) on the same gel. The presence and location of the Fps1 protein band was confirmed by immunoblot using a mouse anti-HA antibody (Sigma); samples were diluted serially to allow accurate comparisons within the linear range of the standard curve. All determinations were done in at least triplicate (n = 3). The intensity of protein bands was determined by quantitative densitometry using ImageJ (http://rsb.info.nih.gov/ij/).

### GFP and HRP yield measurements

GFP yields from recombinant yeast cultures were assayed as previously described [6], except 25 mL cultures were harvested at A_600_ = 4 (just before the diauxic shift) rather than 50 mL cultures at A_600_ = 1. Briefly, samples were withdrawn and the cells pelletted at 5000×*g*, 4 °C for 5 min and the supernatant collected. 200 μL supernatant were loaded in triplicate in a black Nunc MaxiSorp 96-well plate and the fluorescence recorded on a SpectraMax Gemini XS plate reader (Molecular Devices, Wokingham, UK) with excitation and emission wavelengths of 390 and 510 nm respectively, and a cut-off of 495 nm. Doxycycline fluorescence accounted for less than 5% of the signal up to concentrations of 10 μg/mL. HRP yields were measured from recombinant cultures, grown as described for GFP [6], using a commercial kit according to the manufacturer’s instructions (EY Laboratories, Inc).One unit of HRP activity decomposes 1 µmol of peroxide/min at 25 °C.

### Polysome analysis

Polysome analysis was done using an improved methodology compared to that used in our previous study [6]; this resulted in higher-quality profiles. Yeast strains were cultured in shake flasks in YPD to A_600_ 0.5–1, 10 mg cycloheximide were added per 100 mL culture and the cells were incubated for a further 15 min at 30 °C. Cultures were instantly cooled by pouring over ice and the cells were harvested at 5300×*g* for 5 min. All subsequent stages were done at 4 °C in RNAase-free medium. Cells were washed in freshly-prepared lysis buffer (10 mM Tris–HCl pH7.5, 0.1 M NaCl, 30 mM MgCl_2_, 50 μg/mL cycloheximide, 200 μg/mL heparin, 0.2% diethylpyrocarbonate) and harvested at 5300×*g* for 5 min. The pellet was suspended in 250 μL lysis buffer and transferred to a 2 mL breaking tube containing 1 mL glass beads. Cells were broken in a cold TissueLyser (Qiagen) at 50 Hz for 3 min, the cell debris removed by centrifugation (17,000×*g* for 15 min) and the supernatant stored at −80 °C until required. Supernatant containing 150 μg total nucleic acid was loaded onto a 10–50% sucrose gradient made in gradient buffer (50 mM NH_4_Cl, 50 mM Tris-OAc pH7, 12 mM MgCl_2_) and centrifuged at 250,000×*g* for 2 h in a SW41 rotor (Beckman Instruments). Gradients were collected continuously from the top using a Biocomp Gradient profiler at A_254_. Areas under the curves were analysed using ImageJ.

### *GCN4* expression analysis

Yeast cells were transformed with B1805 (or B1805-*HIS* for yTHC*BMS1*) and grown in 2× CBS (with corresponding nutrient selection) to A_600_ ~ 1. Cells were harvested and washed in sterile water at 5300×*g* for 3 min. The pellet was suspended in 500 μL breaking buffer (100 mM Tris–HCl pH 8, 1 mM dithiothreitol, 20% glycerol), transferred to a 2 mL breaking tube containing 1 mL glass beads and 1:500 dilution of protease inhibitor cocktail IV (Calbiochem). Cells were lyzed in a cold TissueLyser (Qiagen) at 50 Hz for 3 min. Cell debris was removed by centrifugation at 17,000×*g* for 15 min; 100 μL cell lysate was added to 900 μL Z buffer (60 mM Na_2_HPO_4_·7H_2_O, 40 mM NaH_2_PO_4_·H_2_O, 10 mM KCl, 1 mM MgSO_4_·7H_2_O pH 7), incubated at 28 °C for 5 min and the reaction started by adding 200μL 4 mg/mL ο-nitrophenyl-β-d-galactopyranoside (ONPG) in Z buffer. The reaction was incubated at 28 °C until a yellow colouration appeared (typically after 15–30 min) and was stopped by adding 500 μL Na_2_CO_3_. β-Galactosidase levels are expressed relative to wild-type.

### eIF2α phosphorylation analysis

For analysis of eIF2α phosphorylation, cells were cultured in the absence (amino-acid-starved cells) or presence (control cells) of amino acids and cells lysates were analysed by immunoblot using anti-eIF2α and anti-phospho-eIF2α antibodies as described previously [39].

### Phenotypic analysis

Fps1 facilitates glycerol efflux from yeast cells under conditions of hypo-osmotic stress; the *fps1Δ* strain is sensitive to osmotic down-shifts [32, 33]. To assess the phenotypic consequences of expressing the different *FPS1*-containing constructs shown in Table 1, yeast cells were exposed to osmotic shock. The consequences of hyperosmotic shock were analyzed by culturing transformed cells in 400 mL 2× CBS without histidine to A_600_ ~ 4. Cultures were then divided into two, harvested at 5300×*g* for 5 min at 4 °C and suspended in either 2× CBS (no shock) or 2× CBS supplemented with 0.8 M NaCl (hyperosmotic shock) that has been pre-warmed to 30 °C. The A_600_ was adjusted to ~4 and 50 mL aliquots were sampled after 15, 30, 60 and 90 min. Cells were spotted (10 µL spots) in 10-fold serial dilutions (made using the same medium in which the cells were originally suspended) onto YNB agar plates with or without 0.8 M NaCl. For polysome analysis of hyperosmotically-shocked cells, cultures were grown to A_600_ ~ 1, the culture was divided into two and the harvested cells were suspended in either 2× CBS or 2× CBS supplemented with 0.8 M NaCl. 50 mL samples were harvested after 15, 30, 60 and 90 min and processed as described under “Polysome analysis” section, above. The consequences of hypo-osmotic shock were analyzed by culturing transformed cells to A_600_ ~ 2 in 2× CBS without histidine and supplemented with 1 M sorbitol. Cells were spotted (10 µL spots) in 10-fold serial dilutions (made using the growth medium) onto YNB plates either with or without 1 M sorbitol. Plates containing 1 M sorbitol were control plates whilst those without sorbitol caused hypo-osmotic shock.

## Additional file

**Additional file 1.** Additional figures, also see http://doi.org/10.17036/researchdata.aston.ac.uk.00000176.

## Abbreviations

ONPG: ο-nitrophenyl-β-d-galactopyranoside
ORF: open reading frame
PIC: pre-initiation complex
uORF: upstream open reading frame
TC: ternary complex
UTR: untranslated region

## References

[1] Bill RM, von der Haar T (2015). Hijacked then lost in translation: the plight of the recombinant host cell in membrane protein structural biology projects. Curr Opin Struct Biol.

[2] Mahalik S, Sharma AK, Mukherjee KJ (2014). Genome engineering for improved recombinant protein expression in *Escherichia coli*. Microb Cell Fact.

[3] Wagner S, Baars L, Ytterberg AJ, Klussmeier A, Wagner CS, Nord O, Nygren PA, van Wijk KJ, de Gier JW (2007). Consequences of membrane protein overexpression in *Escherichia coli*. Mol Cell Proteom.

[4] Marreddy RK, Pinto JP, Wolters JC, Geertsma ER, Fusetti F, Permentier HP, Kuipers OP, Kok J, Poolman B (2011). The response of *Lactococcus lactis* to membrane protein production. PLoS ONE.

[5] Bill RM, Henderson PJ, Iwata S, Kunji ER, Michel H, Neutze R, Newstead S, Poolman B, Tate CG, Vogel H (2011). Overcoming barriers to membrane protein structure determination. Nat Biotechnol.

[6] Bonander N, Darby RA, Grgic L, Bora N, Wen J, Brogna S, Poyner DR, O’Neill MA, Bill RM (2009). Altering the ribosomal subunit ratio in yeast maximizes recombinant protein yield. Microb Cell Fact.

[7] Bonander N, Ferndahl C, Mostad P, Wilks MD, Chang C, Showe L, Gustafsson L, Larsson C, Bill RM (2008). Transcriptome analysis of a respiratory *Saccharomyces cerevisiae* strain suggests the expression of its phenotype is glucose insensitive and predominantly controlled by Hap4, Cat8 and Mig1. BMC Genom.

[8] Bonander N, Hedfalk K, Larsson C, Mostad P, Chang C, Gustafsson L, Bill RM (2005). Design of improved membrane protein production experiments: quantitation of the host response. Protein Sci.

[9] Heiss S, Maurer M, Hahn R, Mattanovich D, Gasser B (2013). Identification and deletion of the major secreted protein of *Pichia pastoris*. Appl Microbiol Biotechnol.

[10] Gasser B, Sauer M, Maurer M, Stadlmayr G, Mattanovich D (2007). Transcriptomics-based identification of novel factors enhancing heterologous protein secretion in yeasts. Appl Environ Microbiol.

[11] Philips J, Herskowitz I (1997). Osmotic balance regulates cell fusion during mating in *Saccharomyces cerevisiae*. J Cell Biol.

[12] Mollapour M, Piper PW (2007). Hog1 mitogen-activated protein kinase phosphorylation targets the yeast Fps1 aquaglyceroporin for endocytosis, thereby rendering cells resistant to acetic acid. Mol Cell Biol.

[13] Wysocki R, Chery CC, Wawrzycka D, Van Hulle M, Cornelis R, Thevelein JM, Tamas MJ (2001). The glycerol channel Fps1p mediates the uptake of arsenite and antimonite in *Saccharomyces cerevisiae*. Mol Microbiol.

[14] Wegierski T, Billy E, Nasr F, Filipowicz W (2001). Bms1p, a G-domain-containing protein, associates with Rcl1p and is required for 18S rRNA biogenesis in yeast. RNA.

[15] Merrick WC (1992). Mechanism and regulation of eukaryotic protein synthesis. Microbiol Rev.

[16] Shenton D, Smirnova JB, Selley JN, Carroll K, Hubbard SJ, Pavitt GD, Ashe MP, Grant CM (2006). Global translational responses to oxidative stress impact upon multiple levels of protein synthesis. J Biol Chem.

[17] Foiani M, Cigan AM, Paddon CJ, Harashima S, Hinnebusch AG (1991). GCD2, a translational repressor of the GCN4 gene, has a general function in the initiation of protein synthesis in *Saccharomyces cerevisiae*. Mol Cell Biol.

[18] Brown AJP, Tuite MF (1998). Yeast gene analysis.

[19] Huisinga KL, Pugh BF (2004). A genome-wide housekeeping role for TFIID and a highly regulated stress-related role for SAGA in *Saccharomyces cerevisiae*. Mol Cell.

[20] Kornberg RD (2005). Mediator and the mechanism of transcriptional activation. Trends Biochem Sci.

[21] Steffen KK, MacKay VL, Kerr EO, Tsuchiya M, Hu D, Fox LA, Dang N, Johnston ED, Oakes JA, Tchao BN (2008). Yeast life span extension by depletion of 60 s ribosomal subunits is mediated by Gcn4. Cell.

[22] Martin-Marcos P, Hinnebusch AG, Tamame M (2007). Ribosomal protein L33 is required for ribosome biogenesis, subunit joining, and repression of GCN4 translation. Mol Cell Biol.

[23] Eisinger DP, Dick FA, Trumpower BL (1997). Qsr1p, a 60S ribosomal subunit protein, is required for joining of 40S and 60S subunits. Mol Cell Biol.

[24] Hinnebusch AG (2005). Translational regulation of GCN4 and the general amino acid control of yeast. Annu Rev Microbiol.

[25] Tuller T, Ruppin E, Kupiec M (2009). Properties of untranslated regions of the *S. cerevisiae* genome. BMC Genom.

[26] Lawless C, Pearson RD, Selley JN, Smirnova JB, Grant CM, Ashe MP, Pavitt GD, Hubbard SJ (2009). Upstream sequence elements direct post-transcriptional regulation of gene expression under stress conditions in yeast. BMC Genom.

[27] Andrews SJ, Rothnagel JA (2014). Emerging evidence for functional peptides encoded by short open reading frames. Nat Rev Genet.

[28] Palam LR, Baird TD, Wek RC (2011). Phosphorylation of eIF2 facilitates ribosomal bypass of an inhibitory upstream ORF to enhance CHOP translation. J Biol Chem.

[29] Zach L, Braunstein I, Stanhill A (2014). Stress-induced start codon fidelity regulates arsenite-inducible regulatory particle-associated protein (AIRAP) translation. J Biol Chem.

[30] Kozak M (1987). Effects of intercistronic length on the efficiency of reinitiation by eucaryotic ribosomes. Mol Cell Biol.

[31] Hinnebusch AG, Lorsch JR (2012). The mechanism of eukaryotic translation initiation: new insights and challenges. Cold Spring Harb Perspect Biol..

[32] Luyten K, Albertyn J, Skibbe WF, Prior BA, Ramos J, Thevelein JM, Hohmann S (1995). Fps1, a yeast member of the MIP family of channel proteins, is a facilitator for glycerol uptake and efflux and is inactive under osmotic stress. EMBO J.

[33] Tamas MJ, Luyten K, Sutherland FC, Hernandez A, Albertyn J, Valadi H, Li H, Prior BA, Kilian SG, Ramos J (1999). Fps1p controls the accumulation and release of the compatible solute glycerol in yeast osmoregulation. Mol Microbiol.

[34] Hedfalk K, Bill RM, Mullins JG, Karlgren S, Filipsson C, Bergstrom J, Tamas MJ, Rydstrom J, Hohmann S (2004). A regulatory domain in the C-terminal extension of the yeast glycerol channel Fps1p. J Biol Chem.

[35] Tamas MJ, Karlgren S, Bill RM, Hedfalk K, Allegri L, Ferreira M, Thevelein JM, Rydstrom J, Mullins JG, Hohmann S (2003). A short regulatory domain restricts glycerol transport through yeast Fps1p. J Biol Chem.

[36] Garre E, Romero-Santacreu L, De Clercq N, Blasco-Angulo N, Sunnerhagen P, Alepuz P (2012). Yeast mRNA cap-binding protein Cbc1/Sto1 is necessary for the rapid reprogramming of translation after hyperosmotic shock. Mol Biol Cell.

[37] Thorsen M, Di Y, Tangemo C, Morillas M, Ahmadpour D, Van der Does C, Wagner A, Johansson E, Boman J, Posas F (2006). The MAPK Hog1p modulates Fps1p-dependent arsenite uptake and tolerance in yeast. Mol Biol Cell.

[38] Rothnie A, Storm J, Campbell J, Linton KJ, Kerr ID, Callaghan R (2004). The topography of transmembrane segment six is altered during the catalytic cycle of P-glycoprotein. J Biol Chem.

[39] Holmes LE, Campbell SG, De Long SK, Sachs AB, Ashe MP (2004). Loss of translational control in yeast compromised for the major mRNA decay pathway. Mol Cell Biol.

