# Supplementary material for: Constitutively-stressed yeast strains are high-yielding for recombinant Fps1: implications for the translational regulation of an aquaporin
Source: Microb Cell Fact. 2017 Mar 9;16:41. doi: 10.1186/s12934-017-0656-2 (PMC5345182; doi:10.1186/s12934-017-0656-2)
Supplement: Supplementary file 1 — Additional file 1. Additional figures, also see http://doi.org/10.17036/researchdata.aston.ac.uk.00000176. [file 12934_2017_656_MOESM1_ESM.pdf]

Supplementary data

Figure S1: Vector map and nucleotide sequence of expression plasmid pYX222

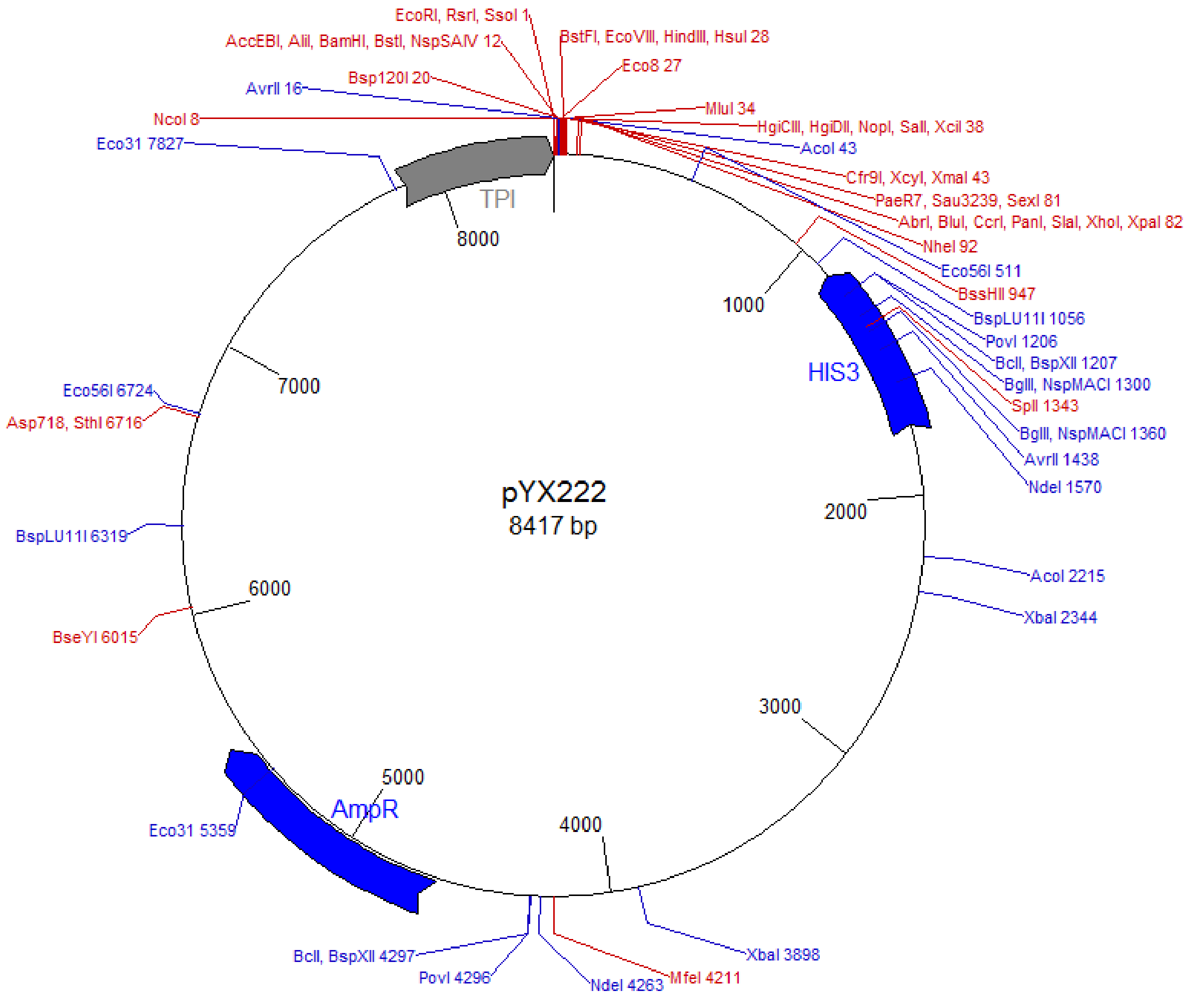

GAATTCACCATGGATCCTAGGGCCCACAAGCTTACGCGTCGACCCGGGTATCCGTATGATGTGCCTGA
CTACGCATGATATCTCGAGCTCAGCTAGCTAACTGAATAAGGAACAATGAACGTTTTTCCTTTCTCTT
GTTCCTAGTATTAATGACTGACCGATACATCCCTTTTTTTTTTTGTCTTTGTCTAGCTCCAATTCGCC
CTATAGTGAGTCGTATTACAATTCACTGGCCGTCGTTTTACAACGTCGTGACTGGGAAAACCCTGGCG
TTACCCAACTTAATCGCCTTGCAGCACATCCCCCTTTCGCCAGCTGGCGTAATAGCGAAGAGGCCCGC
ACCGATCGCCCTTCCCAACAGTTGCGCAGCCTGAATGGCGAATGGACGCGCCCTGTAGCGGCGCATTA
AGCGCGGCGGGTGTGGTGGTTACGCGCAGCGTGACCGCTACACTTGCCAGCGCCCTAGCGCCCGCTCC
TTTCGCTTTCTTCCCTTCCTTTCTCGCCACGTTCGCCGGCTTTCCCCGTCAAGCTCTAAATCGGGGGC
TCCCTTTAGGGTTCCGATTTAGTGCTTTACGGCACCTCGACCCCAAAAAACTTGATTAGGGTGATGGT
TCACGTAGTGGGCCATCGCCCTGATAGACGGTTTTTCGCCCTTTGACGTTGGAGTCCACGTTCTTTAA
TAGTGGACTCTTGTTCCAAACTGGAACAACACTCAACCCTATCTCGGTCTATTCTTTTGATTTATAAG
GGATTTTGCCGATTTCGGCCTATTGGTTAAAAAATGAGCTGATTTAACAAAAATTTAACGCGAATTTT
AACAAAATATTAACGCTTACAATTTCCTGATGCGGTATTTTCTCCTTACGCATCTGTGCGGTATTTCA
CACCGCATAGATCCGTCGAGTTCAAGAGAAAAAAAAAGAAAAAGCAAAAAGAAAAAAGGAAAGCGCGC
CTCGTTCAGAATGACACGTATAGAATGATGCATTACCTTGTCATCTTCAGTATCATACTGTTCGTATA
CATACTTACTGACATTCATAGGTATACATATATACACATGTATATATATCGTATGCTGCAGCTTTAAA

TAATCGGTGTCACTACATAAGAACACCTTTGGTGGAGGGAACATCGTTGGTTCCATTGGGCGAGGTGG
CTTCTCTTATGGCAACCGCAAGAGCCTTGAACGCACTCTCACTACGGTGATGATCATTCTTGCCTCGC
AGACAATCAACGTGGAGGGTAATTCTGCTTGCCTCTGCAAAACTTTCAAGAAAATGCGGGATCATCTC
GCAAGAGAGATCTCCTACTTTCTCCCTCTGCAAACCAAGTTCGACAACTGCGTACGGCCTGTTCGAAA
GATCTACCACCGCTCTGGAAAGTGCCTCATCCAAAGGCGCAAATCCTGATCCAAACCTTTTTACTCCA
CGCACGGCCCCTAGGGCCTCTTTAAATGCTTGACCGAGAGCAATCCCGCAGTCTTCAGTGGTGTGATG
GTCGTCTATGTGTAAGTCACCAATGCACTCAACGATTAGCGACCAGCCGGAATGCTTGGCCAGAGCAT
GTATCATATGGTCCAGAAACCCTATACCTGTGTGGACGTTAATCACTTGCGATTGTGTGGCCTGTTCT
GCTACTGCTTCTGCCTCTTTTTCTGGGAAGATCGAGTGCTCTATCGCTAGGGGACCACCCTTTAAAGA
GATCGCAATCTGAATCTTGGTTTCATTTGTAATACGCTTTACTAGGGCTTTCTGCTCTGTCATCTTTG
CCTTCGTTTATCTTGCCTGCTCATTTTTTAGTATATTCTTCGAAGAAATCACATTACTTTATATAATG
TATAATTCATTATGTGATAATGCCAATCGCTAAGAAAAAAAAAAGAGTCATCCGCTAGGGGAAAAAAAA
AAATGAAAATCATTACCGAGGCATAAAAAAAATATAGAGTGTACTAGAGGAGGCCAAGAGTAATAGAAA
AAGAAAATTGCGGGAAAGGACTGTGTTATGACTTCCCTGACTAATGCCGTGTTCAAACGATACCTGGC
AGTGACTCCTAGCGCTCACCAAGCTCTTAAAACGGGAATTTATGGTGCACTCTCAGTACAATCTGCTC
TGATGCCGCATAGTTAAGCCAGCCCCGACACCCGCCAACACCCGCTGACGCGCCCTGACGGGCTTGTC
TGCTCCCGGCATCCGCTTACAGACAAGCTGTGACCGTCTCCGGGAGCTGCATGTGTCAGAGGTTTTCA
CCGTCATCACCGAAACGCGCGAGACGAAAGGGCCTCGTGATACGCCTATTTTTATAGGTTAATGTCAT
GATAATAATGGTTTCTTAGACGTGCGGCCGCTCTAGAACTAGTGGATCAATTCCACGGACTATAGACT
ATACTAGTATACTCCGTCTACTGTACGATACACTTCCGCTCAGGTCCTTGTCCTTTAACGAGGCCTTA
CCACTCTTTTGTTACTCTATTGATCCAGCTCAGCAAAGGCAGTGTGATCTAAGATTCTATCTTCGCGA
TGTAGTAAAACTAGCTAGACCGAGAAAGAGACTAGAAATGCAAAAGGCACTTCTACAATGGCTGCCAT
CATTATTATCCGATGTGACGCTGCAGCTTCTCAATGATATTCGAATACGCTTTGAGGAGATACAGCCT
AATATCCGACAAACTGTTTTACAGATTTACGATCGTACTTGTTACCCATCATTGAATTTTGAACATCC
GAACCTGGGAGTTTTCCCTGAAACAGATAGTATATTTGAACCTGTATAATAATATATAGTCTAGCGCT
TTACGGAAGACAATGTATGTATTTCGGTTCCTGGAGAAACTATTGCATCTATTGCATAGGTAATCTTG
CACGTCGCATCCCCGGTTCATTTTCTGCGTTTCCATCTTGCACTTCAATAGCATATCTTTGTTAACGA
AGCATCTGTGCTTCATTTTGTAGAACAAAAATGCAACGCGAGAGCGCTAATTTTTCAAACAAAGAATC
TGAGCTGCATTTTTACAGAACAGAAATGCAACGCGAAAGCGCTATTTTACCAACGAAGAATCTGTGCT
TCATTTTTGTAAAACAAAAATGCAACGCGAGAGCGCTAATTTTTCAAACAAAGAATCTGAGCTGCATT
TTTACAGAACAGAAATGCAACGCGAGAGCGCTATTTTACCAACAAAGAATCTATACTTCTTTTTTGTT
CTACAAAAATGCATCCCGAGAGCGCTATTTTTCTAACAAAGCATCTTAGATTACTTTTTTTCTCCTTT
GTGCGCTCTATAATGCAGTCTCTTGATAACTTTTTGCACTGTAGGTCCGTTAAGGTTAGAAGAAGGCT
ACTTTGGTGTCTATTTTCTCTTCCATAAAAAAAGCCTGACTCCACTTCCCGCGTTTACTGATTACTAG
CGAAGCTGCGGGTGCATTTTTTCAAGATAAAGGCATCCCCGATTATATTCTATACCGATGTGGATTGC
GCATACTTTGTGAACAGAAAGTGATAGCGTTGATGATTCTTCATTGGTCAGAAAATTATGAACGGTTT
CTTCTATTTTGTCTCTATATACTACGTATAGGAAATGTTTACATTTTCGTATTGTTTTCGATTCACTC
TATGAATAGTTCTTACTACAATTTTTTTGTCTAAAGAGTAATACTAGAGATAAACATAAAAAAATGTAG
AGGTCGAGTTTAGATGCAAGTTCAAGGAGCGAAAGGTGGATGGGTAGGTTATATAGGGATATAGCACA
GAGATATATAGCAAAGAGATACTTTTGAGCAATGTTTGTGGAAGCGGTATTCGCAATATTTTAGTAGC
TCGTTACAGTCCGGTGCGTTTTTGGTTTTTTGAAAGTGCGTCTTCAGAGCGCTTTTGGTTTTCAAAAG
CGCTCTGAAGTTCCTATACTTTCTAGAGAATAGGAACTTCGGAATAGGAACTTCAAAGCGTTTCCGAA
AACGAGCGCTTCCGAAAATGCAACGCGAGCTGCGCACATACAGCTCACTGTTCACGTCGCACCTATAT
CTGCGTGTTGCCTGTATATATATATACATGAGAAGAACGGCATAGTGCGTGTTTATGCTTAAATGCGT
ACTTATATGCGTCTATTTATGTAGGATGAAAGGTAGTCTAGTACCTCCTGTGATATTATCCCATTCCA
TGCGGGGTATCGTATGCTTCCTTCAGCACTACCCTTTAGCTGTTCTATATGCTGCCACTCCTCAATTG
GATTAGTCTCATCCTTCAATGCTATCATTTCCTTTGATATTGGATCATATGCATAGTACCGAGAAACT
AGTGCGAAGTAGTGATCAGGTATTGCTGTTATCTGATGAGTATACGTTGTCCTGGCCACGGCAGAAGC

ACGCTTATCGCTCCAATTTCCCACAACATTAGTCAACTCCGTTAGGCCCTTCATTGAAAGAAATGAGG
TCATCAAATGTCTTCCAATGTGAGATTTTGGGCCATTTTTTATAGCAAAGATTGAATAAGGCGCATTT
TTCTTCAAAGCTGCGGCCGCACGTCAGGTGGCACTTTTCGGGGAAATGTGCGCGGAACCCCTATTTGT
TTATTTTTCTAAATACATTCAAATATGTATCCGCTCATGAGACAATAACCGTGATAAATGCTTCAATA
ATATTGAAAAAGGAAGAGTATGAGTATTCAACATTTCCGTGTCGCCCTTATTCCCTTTTTTGCGGCAT
TTTGCCTTCCTGTTTTTGCTCACCCAGAAACGCTGGTGAAAGTAAAAGATGCTGAAGATCAGTTGGGT
GCACGAGTGGGTTACATCGAACTGGATCTCAACAGCGGTAAGATCCTTGAGAGTTTTCGCCCCGAAGA
ACGTTTTCCAATGATGAGCACTTTTAAAGTTCTGCTATGTGGCGCGGTATTATCCCGTATTGACGCCG
GGCAAGAGCAACTCGGTCGCCGCATACACTATTCTCAGAATGACTTGGTTGAGTACTCACCAGTCACA
GAAAAGCATCTTACGGATGGCATGACAGTAAGAGAATTATGCAGTGCTGCCATAACCATGAGTGATAA
CACTGCGGCCAACTTACTTCTGACAACGATCGGAGGACCGAAGGAGCTAACCGCTTTTTTGCACAACA
TGGGGGATCATGTAACTCGCCTTGATCGTTGGGAACCGGAGCTGAATGAAGCCATACCAAACGACGAG
CGTGACACCACGATGCCTGTAGCAATGGCAACAACGTTGCGCAAACTATTAACTGGCGAACTACTTAC
TCTAGCTTCCCGGCAACAATTAATAGACTGGATGGAGGCGGATAAAGTTGCAGGACCACTTCTGCGCT
CGGCCCTTCCGGCTGGCTGGTTTATTGCTGATAAATCTGGAGCCGGTGAGCGTGGGTCTCGCGGTATC
ATTGCAGCACTGGGGCCAGATGGTAAGCCCTCCCGTATCGTAGTTATCTACACGACGGGGAGTCAGGC
AACTATGGATGAACGAAATAGACAGATCGCTGAGATAGGTGCCTCACTGATTAAGCATTGGTAACTGT
CAGACCAAGTTTACTCATATATACTTTAGATTGATTTAAAACTTCATTTTTAATTTAAAAGGATCTAG
GTGAAGATCCTTTTTGATAATCTCATGACCAAAATCCCTTAACGTGAGTTTTCGTTCCACTGAGCGTC
AGACCCCGTAGAAAAGATCAAAGGATCTTCTTGAGATCCTTTTTTTCTGCGCGTAATCTGCTGCTTGC
AAACAAAAAAACCACCGCTACCAGCGGTGGTTTGTTTGCCGGATCAAGAGCTACCAACTCTTTTTCCG
AAGGTAACTGGCTTCAGCAGAGCGCAGATACCAAATACTGTTCTTCTAGTGTAGCCGTAGTTAGGCCA
CCACTTCAAGAACTCTGTAGCACCGCCTACATACCTCGCTCTGCTAATCCTGTTACCAGTGGCTGCTG
CCAGTGGCGATAAGTCGTGTCTTACCGGGTTGGACTCAAGACGATAGTTACCGGATAAGGCGCAGCGG
TCGGGCTGAACGGGGGGTTCGTGCACACAGCCCAGCTTGGAGCGAACGACCTACACCGAACTGAGATA
CCTACAGCGTGAGCTATGAGAAAGCGCCACGCTTCCCGAAGGGAGAAAGGCGGACAGGTATCCGGTAA
GCGGCAGGGTCGGAACAGGAGAGCGCACGAGGGAGCTTCCAGGGGGAAACGCCTGGTATCTTTATAGT
CCTGTCGGGTTTCGCCACCTCTGACTTGAGCGTCGATTTTTGTGATGCTCGTCAGGGGGGCGGAGCCT
ATGGAAAAACGCCAGCAACGCGGCCTTTTTACGGTTCCTGGCCTTTTGCTGGCCTTTTGCTCACATGT
TCTTTCCTGCGTTATCCCCTGATTCTGTGGATAACCGTATTACCGCCTTTGAGTGAGCTGATACCGCT
CGCCGCAGCCGAACGACCGAGCGCAGCGAGTCAGTGAGCGAGGAAGCGGAAGAGCGCCCAATACGCAA
ACCGCCTCTCCCCGCGCGTTGGCCGATTCATTAATGCAGCTGGCACGACAGGTTTCCCGACTGGAAAG
CGGGCAGTGAGCGCAACGCAATTAATGTGAGTTAGCTCACTCATTAGGCACCCCAGGCTTTACACTTT
ATGCTTCCGGCTCGTATGTTGTGTGGAATTGTGAGCGGATAACAATTTCACACAGGAAACAGCTATGA
CCATGATTACGCCAAGCTCGAAATTAACCCTCACTAAAGGGAACAAAAGCTGGTACCGGGCCGGCCGT
CGGGCCGTCGAGCTTGATGGCATCGTGGTGTCACGCTCGTCGTTTGGTATGGCTTCATTCAGCTCCGG
TTCCCAACGATCAAGGCGAGTTACATGATCCCCCATGTTGTGCAAAAAAGCGGTTAGCTCCTTCGGTC
CTCCGATCGTTGTCAGAAGTAAGTTGGCCGCAGTGTTATCACTCATGGTTATGGCAGCACTGCATAAT
TCTCTTACTGTCATGCCATCCGTAAGATGCTTTTCTGTGACTGGTGAGTACTCAACCAAGTCATTCTG
AGAATAGTGTATGCGGCGACCGAGTTGCTCTTGCCCGGCGTCAACACGGGATAATACCGCGCCACATA
GCAGAACTTTAAAAGTGCTCATCATTGGAAAACGTTCTTCGGGGCGAAAACTCTCAAGGATCTTACCG
CTGTTGAGATCCAGTTCGATGTAACCCACTCGTGCACCCAACTGATCTTCAGCATCTTTTACTTTCAC
CAGCGTTTCTGGGTGAGCAAAAACAGGAAGGCAAAATGCCGCAAAAAAGGGAATAAGGGCGACACGGA
AATGTTGAATACTCATACTCTTCCTTTTTCAATATTATTGAAGCATTTATCAGGGTTATTGTCTCATG
AGCGGATACATATTTGAATGTATTTAGAAAAATAAACAAATAGGGGTTCCGCGCACATTTCCCCGAAA
AGTGCCACCTGACGTCTAAGAAACCATTATTATCATGACATTAACCTATAAAAATAGGCGTATCACGA
GGCCCTTTCGTCTTCAAGAATTGGGGATCTACGTATGGTCATTTCTTCTTCAGATTCCCTCATGGAGA
AAGTGCGGCAGATGTATATGACAGAGTCGCCAGTTTCCAAGAGACTTTATTCAGGCACTTCCATGATA

GGCAAGAGAGAAGACCCAGAGATGTTGTTGTCCTAGTTACACATGGTATTTATTCCAGAGTATTCCTG
ATGAAATGGTTTAGATGGACATACGAAGAGTTTGAATCGTTTACCAATGTTCCTAACGGGAGCGTAAT
GGTGATGGAACTGGACGAATCCATCAATAGATACGTCCTGAGGACCGTGCTACCCAAATGGACTGATT
GTGAGGGAGACCTAACTACATAGTGTTTAAAGATTACGGATATTTAACTTACTTAGAATAATGCCATT
TTTTTGAGTTATAATAATCCTACGTTAGTGTGAGCGGGATTTAAACTGTGAGGACCTTAATACATTCA
GACACTTCTGCGGTATCACCCTACTTATTCCCTTCGAGATTATATCTAGGAACCCATCAGGTTGGTGG
AAGATTACCCGTTCTAAGACTTTTCAGCTTCCTCTATTGATGTTACACCTGGACACCCCTTTTCTGGC
ATCCAGTTTTTAATCTTCAGTGGCATGTGAGATTCTCCGAAATTAATTAAAGCAATCACACAATTCTC
TCGGATACCACCTCGGTTGAAACTGACAGGTGGTTTGTTACGCATGCTAATGCAAAGGAGCCTATATA
CCTTTGGCTCGGCTGCTGTAACAGGGAATATAAAGGGCAGCATAATTTAGGAGTTTAGTGAACTTGCA
ACATTTACTATTTTCCCTTCTTACGTAAATATTTTTCTTTTTAATTCTAAATCAATCTTTTTCAATTT
TTTGTTTGTATTCTTTTCTTGCTTAAATCTATAACTACAAAAAACACATACAG

Figure S2: Vector map and nucleotide sequence of expression plasmid pYX222-*FPS1*-HA$_3$

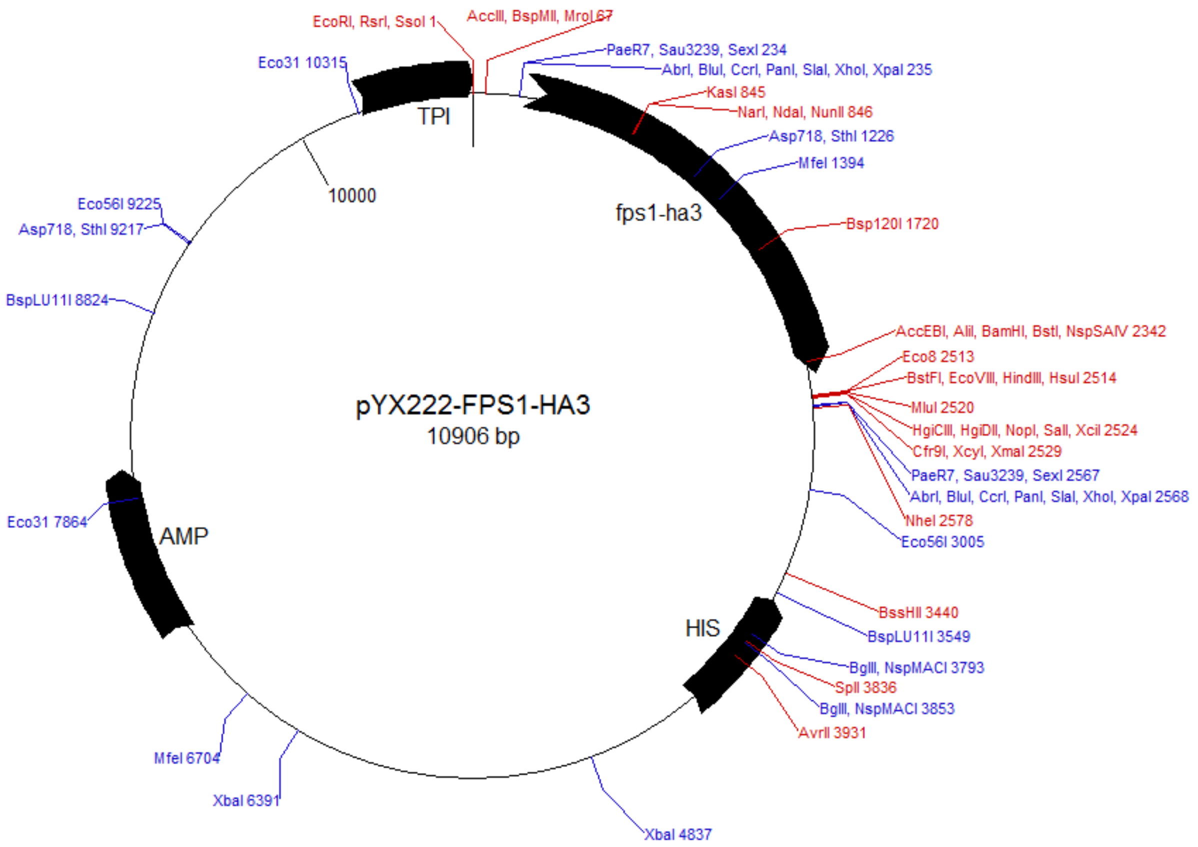

Note: The *FPS1* 5´UTR is denoted in red (with the original *BamHI*/*Bgl*II cloning location in *italic*; the transcriptional start site is 79 nucleotides upstream of the *FPS1* ORF [25] and is denoted in **bold red font**. The *FPS1* ORF is in grey highlight and the sequence encoding HA$_3$ is in green. The nucleotide at position 1 is denoted in **bold** and the uORFs are underlined.

GAATTC**A**CCAT*GGATCT*CATAGTGAGAAGGCGCAATTCAGTAGTTAAAAGCGGGGAACAGTGTGAATC
CGGAGACGGCAAGATTGCCCGGCCCTTTTTGCGGAAAAGATAAAACAAGATATATTGCACTTTTTCCA
CCAAGAAAAACAGGAAGTGGATTAAAAAATCAACAAAGTATAACGCCTAT**T**GTCCCAATAAGCGTCGG
TTGTTCTTCTTTATTATTTTACCAAGTACGCTCGAGGGTACATTCTAATGCATTAAAAGACATGAGTA
ATCCTCAAAAAGCTCTAAACGACTTTCTGTCCAGTGAATCTGTTCATACACATGATAGTTCTAGGAAA
CAATCTAATAAGCAGTCATCCGACGAAGGACGCTCTTCATCACAACCTTCACATCATCACTCTGGTGG
TACTAACAACAATAATAACAATAATAATAATAATAATAACAGTAACAACAACAACAACGGCAACGATG
GGGGAAATGATGACGACTATGATTATGAAATGCAAGATTATAGACCTTCTCCGCAAAGTGCGCGGCCT
ACTCCCACGTATGTTCCACAATATTCTGTAGAAAGTGGGACTGCTTTCCCGATTCAAGAGGTTATTCC
TAGCGCATACATTAACACACAAGATATAAACCATAAAGATAACGGTCCGCCGAGTGCAAGCAGTAATA
GAGCATTCAGGCCTAGAGGGCAGACCACAGTGTCGGCCAACGTGCTTAACATTGAAGATTTTTACAAA
AATGCAGACGATGCGCATACCATCCCGGAGTCACATTTATCGAGAAGGAGAAGTAGGTCGAGGGCTAC
GAGTAATGCTGGGCACAGTGCCAATACAGGCGCCACGAATGGCAGGACTACTGGTGCCCAAACTAATA
TGGAAAGCAATGAATCACCACGTAACGTCCCCATTATGGTGAAGCCAAAGACATTATACCAGAACCCT
CAAACACCTACAGTCTTGCCCTCCACATACCATCCAATTAATAAATGGTCTTCCGTCAAAAACACTTA
TTTGAAGGAATTTTTAGCCGAGTTTATGGGAACAATGGTTATGATTATTTTCGGTAGTGCTGTTGTTT
GTCAGGTCAATGTTGCTGGGAAAATACAGCAGGACAATTTCAACGTGGCTTTGGATAACCTTAACGTT

ACCGGGTCTTCTGCAGAAACGATAGACGCTATGAAGAGTTTAACATCCTTGGTTTCATCCGTTGCGGG
CGGTACCTTTGATGATGTGGCATTGGGCTGGGCTGCTGCCGTGGTGATGGGCTATTTCTGCGCTGGTG
GTAGTGCCATCTCAGGTGCTCATTTGAATCCGTCTATTACATTAGCCAATTTGGTGTATAGAGGTTTT
CCCCTGAAGAAAGTTCCTTATTACTTTGCTGGACAATTGATCGGTGCCTTCACAGGCGCTTTGATCTT
GTTTATTTGGTACAAAAGGGTGTTACAAGAGGCATATAGCGATTGGTGGATGAATGAAAGTGTTGCGG
GAATGTTTTGCGTTTTTCCAAAGCCTTATCTAAGTTCAGGACGGCAATTTTTTTCCGAATTTTTATGT
GGAGCTATGTTACAAGCAGGAACATTTGCGCTGACCGATCCTTATACGTGTTTGTCCTCTGATGTTTT
CCCATTGATGATGTTTATTTTGATTTTCATTATCAATGCTTCCATGGCTTATCAGACAGGTACAGCAA
TGAATTTGGCTCGTGATCTGGGCCCACGTCTTGCACTATATGCAGTTGGATTTGATCATAAAATGCTT
TGGGTGCATCATCATCATTTCTTTTGGGTTCCCATGGTAGGCCCATTTATTGGTGCGTTAATGGGGGG
GTTGGTTTACGATGTCTGTATTTATCAGGGTCATGAATCTCCAGTCAACTGGTCTTTACCAGTTTATA
AGGAAATGATTATGAGAGCCTGGTTTAGAAGGCCTGGTTGGAAGAAGAGAAATAGAGCAAGAAGAACA
TCGGACCTGAGTGACTTCTCATACAATAACGATGATGATGAGGAATTTGGAGAAAGAATGGCTCTTCA
AAAGACAAAGACCAAGTCATCTATTTCAGACAACGAAAATGAAGCAGGAGAAAAGAAAGTGCAATTTA
AATCTGTTCAGCGCGGCAAAAGAACGTTTGGTGGTATACCAACAATTCTTGAAGAAGAAGATTCCATT
GAAACTGCTTCGCTAGGTGCGACGACGACTGATTCTATTGGGTTATCCGACACATCATCAGAAGATTC
GCATTATGGTAATGCTAAGAAGGTAAGCGGCCGCATCTTTTACCCATACGATGTTCCTGACTATGCGG
GCTATCCCTATGACGTCCCGGACTATGCAGGATCCTATCCATATGACGTTCCAGATTACGCTGCTCAG
TGCGGCCGCTGAGAAAACAGACAAGAAAAAGAAACAAATAATATAGACTGATAGAAAAAAATACTGCT
TACTACCGCCGGTATAATATATATATATATATATATTTACATAGATGATTGCATAGTGTTTTAAAAAG
CTTACGCGTCGACCCGGGTATCCGTATGATGTGCCTGACTACGCATGATATCTCGAGCTCAGCTAGCT
AACTGAATAAGGAACAATGAACGTTTTTCCTTTCTCTTGTTCCTAGTATTAATGACTGACCGATACAT
CCCTTTTTTTTTTTGTCTTTGTCTAGCTCCAGCTTTTGTTCCCTTTAGTGAGGGTTAATTCAATTCAC
TGGCCGTCGTTTTACAACGTCGTGACTGGGAAAACCCTGGCGTTACCCAACTTAATCGCCTTGCAGCA
CATCCCCCTTTCGCCAGCTGGCGTAATAGCGAAGAGGCCCGCACCGATCGCCCTTCCCAACAGTTGCG
CAGCCTGAATGGCGAATGGCGCGACGCGCCCTGTAGCGGCGCATTAAGCGCGGCGGGTGTGGTGGTTA
CGCGCAGCGTGACCGCTACACTTGCCAGCGCCCTAGCGCCCGCTCCTTTCGCTTTCTTCCCTTCCTTT
CTCGCCACGTTCGCCGGCTTTCCCCGTCAAGCTCTAAATCGGGGGCTCCCTTTAGGGTTCCGATTTAG
TGCTTTACGGCACCTCGACCCCAAAAAACTTGATTAGGGTGATGGTTCACGTAGTGGGCCATCGCCCT
GATAGACGGTTTTTCGCCCTTTGACGTTGGAGTCCACGTTCTTTAATAGTGGACTCTTGTTCCAAACT
GGAACAACACTCAACCCTATCTCGGTCTATTCTTTTGATTTATAAGGGATTTTGCCGATTTCGGCCTA
TTGGTTAAAAAATGAGCTGATTTAACAAAAATTTAACGCGAATTTTAACAAAATATTAACGCTTACAA
TTTCCTGATGCGGTATTTTCTCCTTACGCATCTGTGCGGTATTTCACACCGCATAATCCGTCGAGTTC
AAGAGAAAAAAAAAGAAAAAGCAAAAAGAAAAAAGGAAAGCGCGCCTCGTTCAGAATGACACGTATAG
AATGATGCATTACCTTGTCATCTTCAGTATCATACTGTTCGTATACATACTTACTGACATTCATAGGT
ATACATATATACACATGTATATATATCGTATGCTGCAGCTTTAAATAATCGGTGTCACTACATAAGAA
CACCTTTGGTGGAGGGAACATCGTTGGTTCCATTGGGCGAGGTGGCTTCTCTTATGGCAACCGCAAGA
GCCTTGAACGCACTCTCACTACGGTGATGATCATTCTTGCCTCGCAGACAATCAACGTGGAGGGTAAT
TCTGCTTGCCTCTGCAAAACTTTCAAGAAAATGCGGGATCATCTCGCAAGAGAGATCTCCTACTTTCT
CCCTCTGCAAACCAAGTTCGACAACTGCGTACGGCCTGTTCGAAAGATCTACCACCGCTCTGGAAAGT
GCCTCATCCAAAGGCGCAAATCCTGATCCAAACCTTTTTACTCCACGCACGGCCCCTAGGGCCTCTTT
AAATGCTTGACCGAGAGCAATCCCGCAGTCTTCAGTGGTGTGATGGTCGTCTATGTGTAAGTCACCAA
TGCACTCAACGATTAGCGACCAGCCGGAATGCTTGGCCAGAGCATGTATCATATGGTCCAGAAACCCT
ATACCTGTGTGGACGTTAATCACTTGCGATTGTGTGGCCTGTTCTGCTACTGCTTCTGCCTCTTTTTC
TGGGAAGATCGAGTGCTCTATCGCTAGGGGACCACCCTTTAAAGAGATCGCAATCTGAATCTTGGTTT
CATTTGTAATACGCTTTACTAGGGCTTTCTGCTCTGTCATCTTTGCCTTCGTTTATCTTGCCTGCTCA
TTTTTTAGTATATTCTTCGAAGAAATCACATTACTTTATATAATGTATAATTCATTATGTGATAATGC
CAATCGCTAAGAAAAAAAAAGAGTCATCCGCTAGGGGAAAAAAAAAAAATGAAAATCATTACCGAGGCA

TAAAAAAATATAGAGTGTACTAGAGGAGGCCAAGAGTAATAGAAAAAGAAAATTGCGGGAAAGGACTG
TGTTATGACTTCCCTGACTAATGCCGTGTTCAAACGATACCTGGCAGTGACTCCTAGCGCTCACCAAG
CTCTTAAAACGGGAATTTATGGTGCACTCTCAGTACAATCTGCTCTGATGCCGCATAGTTAAGCCAGC
CCCGACACCCGCCAACACCCGCTGACGCGCCCTGACGGGCTTGTCTGCTCCCGGCATCCGCTTACAGA
CAAGCTGTGACCGTCTCCGGGAGCTGCATGTGTCAGAGGTTTTCACCGTCATCACCGAAACGCGCGAG
ACGAAAGGGCCTCGTGATACGCCTATTTTTATAGGTTAATGTCATGATAATAATGGTTTCTTAGACGT
GCGGCCGCTCTAGAACTAGTGGATCAATTCCACGGACTATAGACTATACTAGTATACTCCGTCTACTG
TACGATACACTTCCGCTCAGGTCCTTGTCCTTTAACGAGGCCTTACCACTCTTTTGTTACTCTATTGA
TCCAGCTCAGCAAAGGCAGTGTGATCTAAGATTCTATCTTCGCGATGTAGTAAAACTAGCTAGACCGA
GAAAGAGACTAGAAATGCAAAAGGCACTTCTACAATGGCTGCCATCATTATTATCCGATGTGACGCTG
CAGCTTCTCAATGATATTCGAATACGCTTTGAGGAGATACAGCCTAATATCCGACAAACTGTTTTACA
GATTTACGATCGTACTTGTTACCCATCATTGAATTTTGAACATCCGAACCTGGGAGTTTTCCCTGAAA
CAGATAGTATATTTGAACCTGTATAATAATATATAGTCTAGCGCTTTACGGAAGACAATGTATGTATT
TCGGTTCCTGGAGAAACTATTGCATCTATTGCATAGGTAATCTTGCACGTCGCATCCCCGGTTCATTT
TCTGCGTTTCCATCTTGCACTTCAATAGCATATCTTTGTTAACGAAGCATCTGTGCTTCATTTTGTAG
AACAAAAATGCAACGCGAGAGCGCTAATTTTTCAAACAAAGAATCTGAGCTGCATTTTTACAGAACAG
AAATGCAACGCGAAAGCGCTATTTTACCAACGAAGAATCTGTGCTTCATTTTTGTAAAACAAAAATGC
AACGCGAGAGCGCTAATTTTTCAAACAAAGAATCTGAGCTGCATTTTTACAGAACAGAAATGCAACGC
GAGAGCGCTATTTTACCAACAAAGAATCTATACTTCTTTTTTGTTCTACAAAAATGCATCCCGAGAGC
GCTATTTTTCTAACAAAGCATCTTAGATTACTTTTTTTCTCCTTTGTGCGCTCTATAATGCAGTCTCT
TGATAACTTTTTGCACTGTAGGTCCGTTAAGGTTAGAAGAAGGCTACTTTGGTGTCTATTTTCTCTTC
CATAAAAAAAGCCTGACTCCACTTCCCGCGTTTACTGATTACTAGCGAAGCTGCGGGTGCATTTTTTC
AAGATAAAGGCATCCCCGATTATATTCTATACCGATGTGGATTGCGCATACTTTGTGAACAGAAAGTG
ATAGCGTTGATGATTCTTCATTGGTCAGAAAATTATGAACGGTTTCTTCTATTTTGTCTCTATATACT
ACGTATAGGAAATGTTTACATTTTCGTATTGTTTTCGATTCACTCTATGAATAGTTCTTACTACAATT
TTTTTGTCTAAAGAGTAATACTAGAGATAAACATAAAAAATGTAGAGGTCGAGTTTAGATGCAAGTTC
AAGGAGCGAAAGGTGGATGGGTAGGTTATATAGGGATATAGCACAGAGATATATAGCAAAGAGATACT
TTTGAGCAATGTTTGTGGAAGCGGTATTCGCAATATTTTAGTAGCTCGTTACAGTCCGGTGCGTTTTT
GGTTTTTTGAAAGTGCGTCTTCAGAGCGCTTTTGGTTTTCAAAAGCGCTCTGAAGTTCCTATACTTTC
TAGAGAATAGGAACTTCGGAATAGGAACTTCAAAGCGTTTCCGAAAACGAGCGCTTCCGAAAATGCAA
CGCGAGCTGCGCACATACAGCTCACTGTTCACGTCGCACCTATATCTGCGTGTTGCCTGTATATATAT
ATACATGAGAAGAACGGCATAGTGCGTGTTTATGCTTAAATGCGTACTTATATGCGTCTATTTATGTA
GGATGAAAGGTAGTCTAGTACCTCCTGTGATATTATCCCATTCCATGCGGGGTATCGTATGCTTCCTT
CAGCACTACCCTTTAGCTGTTCTATATGCTGCCACTCCTCAATTGGATTAGTCTCATCCTTCAATGCT
ATCATTTCCTTTGATATTGGATCATATGCATAGTACCGAGAAACTAGTGCGAAGTAGTGATCAGGTAT
TGCTGTTATCTGATGAGTATACGTTGTCCTGGCCACGGCAGAAGCACGCTTATCGCTCCAATTTCCCA
CAACATTAGTCAACTCCGTTAGGCCCTTCATTGAAAGAAATGAGGTCATCAAATGTCTTCCAATGTGA
GATTTTGGGCCATTTTTTATAGCAAAGATTGAATAAGGCGCATTTTTCTTCAAAGCTGCGGCCGCACT
CTCACTAGTACGTCAGGTGGCACTTTTCGGGGAAATGTGCGCGGAACCCCTATTTGTTTATTTTTCTA
AATACATTCAAATATGTATCCGCTCATGAGACAATAACCCTGATAAATGCTTCAATAATATTGAAAAA
GGAAGAGTATGAGTATTCAACATTTCCGTGTCGCCCTTATTCCCTTTTTTGCGGCATTTTGCCTTCCT
GTTTTTGCTCACCCAGAAACGCTGGTGAAAGTAAAAGATGCTGAAGATCAGTTGGGTGCACGAGTGGG
TTACATCGAACTGGATCTCAACAGCGGTAAGATCCTTGAGAGTTTTCGCCCCGAAGAACGTTTTCCAA
TGATGAGCACTTTTAAAGTTCTGCTATGTGGCGCGGTATTATCCCGTATTGACGCCGGGCAAGAGCAA
CTCGGTCGCCGCATACACTATTCTCAGAATGACTTGGTTGAGTACTCACCAGTCACAGAAAAGCATCT
TACGGATGGCATGACAGTAAGAGAATTATGCAGTGCTGCCATAACCATGAGTGATAACACTGCGGCCA
ACTTACTTCTGACAACGATCGGAGGACCGAAGGAGCTAACCGCTTTTTTGCACAACATGGGGGATCAT
GTAACTCGCCTTGATCGTTGGGAACCGGAGCTGAATGAAGCCATACCAAACGACGAGCGTGACACCAC

GATGCCTGTAGCAATGGCAACAACGTTGCGCAAACTATTAACTGGCGAACTACTTACTCTAGCTTCCC
GGCAACAATTAATAGACTGGATGGAGGCGGATAAAGTTGCAGGACCACTTCTGCGCTCGGCCCTTCCG
GCTGGCTGGTTTATTGCTGATAAATCTGGAGCCGGTGAGCGTGGGTCTCGCGGTATCATTGCAGCACT
GGGGCCAGATGGTAAGCCCTCCCGTATCGTAGTTATCTACACGACGGGGAGTCAGGCAACTATGGATG
AACGAAATAGACAGATCGCTGAGATAGGTGCCTCACTGATTAAGCATTGGTAACTGTCAGACCAAGTT
TACTCATATATACTTTAGATTGATTTAAAACTTCATTTTTAATTTAAAAGGATCTAGGTGAAGATCCT
TTTTGATAATCTCATGACCAAAATCCCTTAACGTGAGTTTTCGTTCCACTGAGCGTCAGACCCCGTAG
AAAAGATCAAAGGATCTTCTTGAGATCCTTTTTTTCTGCGCGTAATCTGCTGCTTGCAAACAAAAAAA
CCACCGCTACCAGCGGTGGTTTGTTTGCCGGATCAAGAGCTACCAACTCTTTTTCCGAAGGTAACTGG
CTTCAGCAGAGCGCAGATACCAAATACTGTTCTTCTAGTGTAGCCGTAGTTAGGCCACCACTTCAAGA
ACTCTGTAGCACCGCCTACATACCTCGCTCTGCTAATCCTGTTACCAGTGGCTGCTGCCAGTGGCGAT
AAGTCGTGTCTTACCGGGTTGGACTCAAGACGATAGTTACCGGATAAGGCGCAGCGGTCGGGCTGAAC
GGGGGGTTCGTGCACACAGCCCAGCTTGGAGCGAACGACCTACACCGAACTGAGATACCTACAGCGTG
AGCTATGAGAAAGCGCCACGCTTCCCGAAGGGAGAAAGGCGGACAGGTATCCGGTAAGCGGCAGGGTC
GGAACAGGAGAGCGCACGAGGGAGCTTCCAGGGGGAAACGCCTGGTATCTTTATAGTCCTGTCGGGTT
TCGCCACCTCTGACTTGAGCGTCGATTTTTGTGATGCTCGTCAGGGGGGCGGAGCCTATGGAAAAACG
CCAGCAACGCGGCCTTTTTACGGTTCCTGGCCTTTTGCTGGCCTTTTGCTCACATGTTCTTTCCTGCG
TTATCCCCTGATTCTGTGGATAACCGTATTACCGCCTTTGAGTGAGCTGATACCGCTCGCCGCAGCCG
AACGACCGAGCGCAGCGAGTCAGTGAGCGAGGAAGCGGAAGAGCGCCCAATACGCAAACCGCCTCTCC
CCGCGCGTTGGCCGATTCATTAATGCAGCTGGCACGACAGGTTTCCCGACTGGAAAGCGGGCAGTGAG
CGCAACGCAATTAATGTGAGTTAGCTCACTCATTAGGCACCCCAGGCTTTACACTTTATGCTTCCGGC
TCGTATGTTGTGTGGAATTGTGAGCGGATAACAATTTCACACAGGAAACAGCTATGACCATGATTACG
CCAAGCTCGAAATACGACTCACTATAGGGCGAATTGGGTACCGGGCCGGCCGTCGAGCTTGATGGCAT
CGTGGTGTCACGCTCGTCGTTTGGTATGGCTTCATTCAGCTCCGGTTCCCAACGATCAAGGCGAGTTA
CATGATCCCCCATGTTGTGCAAAAAAGCGGTTAGCTCTTCGGTCCTCCGATCGTTGTCAGAAGTAAGT
TGGCCGCAGTGTTATCACTCATGGTTATGGCAGGAACTGCATAATTCTCTTACTGTCATGCCATCCGT
AAGATGCTTTTCTGTGACTGGTGTACTCAACCAAGTCATTCTGAGAATAGTGTATGCGGCGACCGAGT
TGCTCTTGCCCGGCGTCAACACGGGATAATACCGCGCCACATAGCAGAACTTTAAAAGTGCTCATCAT
TGGAAAACGTTCTTCGGGGCGAAAACTCTCAAGGATCTTACCGCTGTTGAGATCCAGTTCGATGTAAC
CCACTCGTGCACCCAACTGATCTTCAGCATCTTTTACTTTCACCAGCGTTTCTGGGTGAGCAAAAACA
GGAAGGCAAAATGCCGCAAAAAAGGGAATAAGGGCGACACGGAAATGTTGAATACTCATACTCTTCCT
TTTTCAATATTATTGAAGCATTTATCAGGGTTATTGTCTCATGAGCGATACATATTTGAATGTATTTA
GAAAAATAAACAAATAGGGGTTCCGCGCACATTTCCCCGAAAAGTGCCACCTGACGTCTAAGAAACCA
TTATTATCATGACATTAACCTATAAAAATAGGCGTATCACGAGGCCCTTTCGTCTTCAAGAATTGGGG
ATCTACGTATGGTCATTCTTCTTCAGATTCCCTCATGGAGAAGTGCGGCAGATGTATATGACAGAGTC
GCCAGTTTCCAAGAGACTTTATTCAGGCACTTCCATGATAGGCAAGAGAGAAGACCCAGAGATGTTGT
TGTCCTAGTTACACATGGTATTTATTCCAGAGTATTCCTGATGAAATGGTTTAGATGGACATACGAAG
AGTTTGAATCGTTTACCAATGTTCCTAACGGGAGCGTAATGGTGATGGAACTGGACGAATCCATCAAT
AGATACGTCCTGAGGACCGTGCTACCCAAATGGACTGATTGTGAGGGAGACCTAACTACATAGTGTTT
AAAGATTACGGATATTTAACTTACTTAGAATAATGCCATTTTTTTGAGTTATAATAATCCTACGTTAG
TGTGAGCGGGATTTAAACTGTGAGGACCTCAATACATTCAGACACTTCTGACGGTATCACCCTACTTA
TTCCCTTCGAGATTATATCTAGGAACCCATCAGGTTGGTGGAAGATTACCCGTTCTAAGACTTTTCAG
CTTCCTCTATTGATGTTACACTCGGACACCCCTTTTCTGGCATCCAGTTTTTAATCTTCAGTGGCATG
TGAGATTCTCCGAAATTAATTAAAGCAATCACACAATTCTCTCGGATACCACCTCGGTTGAAACTGAC
AGGTGGTTTGTTACGCATGCTAATGCAAAGGAGCCTATATACCTTTGGCTCGGCTGCTGTAACAGGGA
ATATAAAGGGCAGCATAATTTAGGAGTTTAGTGAACTTGCAACATTTACTATTTTCCCTTCTTACGTA
AATATTTTTCTTTTTAATTCTAAATCAATCTTTTTCAATTTTTTGTTTGTATTCTTTTCTTGCTTAAA
TCTATAACTACAAAAAACACATACAG

Figure S3: Vector map and nucleotide sequence of expression plasmid pYX222-5´Δ1-43-*FPS1*-HA$_3$

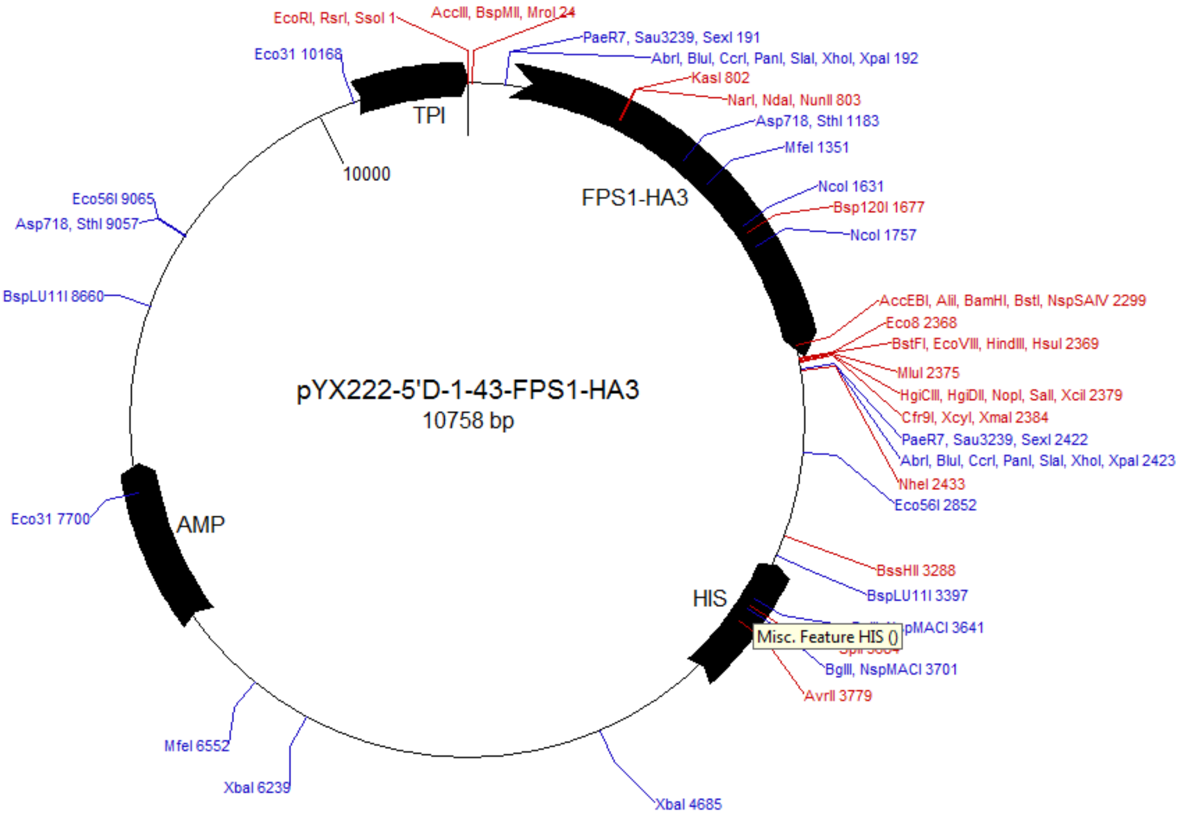

GAATTCGCGGGGAACAGTGTGAATCCGGAGACGGCAAGATTGCCCGGCCCTTTTTGCGGAAAAGATAA
AACAAGATATATTGCACTTTTTCCACCAAGAAAAACAGGAAGTGGATTAAAAAATCAACAAAGTATAA
CGCCTATTGTCCCAATAAGCGTCGGTTGTTCTTCTTTATTATTTTACCAAGTACGCTCGAGGGTACAT
TCTAATGCATTAAAAGACATGAGTAATCCTCAAAAAGCTCTAAACGACTTTCTGTCCAGTGAATCTGT
TCATACACATGATAGTTCTAGGAAACAATCTAATAAGCAGTCATCCGACGAAGGACGCTCTTCATCAC
AACCTTCACATCATCACTCTGGTGGTACTAACAACAATAATAACAATAATAATAATAATAATAACAGT
AACAACAACAACAACGGCAACGATGGGGGAAATGATGACGACTATGATTATGAAATGCAAGATTATAG
ACCTTCTCCGCAAAGTGCGCGGCCTACTCCCACGTATGTTCCACAATATTCTGTAGAAAGTGGGACTG
CTTTCCCGATTCAAGAGGTTATTCCTAGCGCATACATTAACACACAAGATATAAACCATAAAGATAAC
GGTCCGCCGAGTGCAAGCAGTAATAGAGCATTCAGGCCTAGAGGGCAGACCACAGTGTCGGCCAACGT
GCTTAACATTGAAGATTTTTACAAAAATGCAGACGATGCGCATACCATCCCGGAGTCACATTTATCGA
GAAGGAGAAGTAGGTCGAGGGCTACGAGTAATGCTGGGCACAGTGCCAATACAGGCGCCACGAATGGC
AGGACTACTGGTGCCCAAACTAATATGGAAAGCAATGAATCACCACGTAACGTCCCCATTATGGTGAA
GCCAAAGACATTATACCAGAACCCTCAAACACCTACAGTCTTGCCCTCCACATACCATCCAATTAATA
AATGGTCTTCCGTCAAAAACACTTATTTGAAGGAATTTTTAGCCGAGTTTATGGGAACAATGGTTATG
ATTATTTTCGGTAGTGCTGTTGTTTGTCAGGTCAATGTTGCTGGGAAAATACAGCAGGACAATTTCAA
CGTGGCTTTGGATAACCTTAACGTTACCGGGTCTTCTGCAGAAACGATAGACGCTATGAAGAGTTTAA
CATCCTTGGTTTCATCCGTTGCGGGCGGTACCTTTGATGATGTGGCATTGGGCTGGGCTGCTGCCGTG
GTGATGGGCTATTTCTGCGCTGGTGGTAGTGCCATCTCAGGTGCTCATTTGAATCCGTCTATTACATT
AGCCAATTTGGTGTATAGAGGTTTTCCCCTGAAGAAAGTTCCTTATTACTTTGCTGGACAATTGATCG
GTGCCTTCACAGGCGCTTTGATCTTGTTTATTTGGTACAAAAGGGTGTTACAAGAGGCATATAGCGAT

TGGTGGATGAATGAAAGTGTTGCGGGAATGTTTTGCGTTTTTCCAAAGCCTTATCTAAGTTCAGGACG
GCAATTTTTTTCCGAATTTTTATGTGGAGCTATGTTACAAGCAGGAACATTTGCGCTGACCGATCCTT
ATACGTGTTTGTCCTCTGATGTTTTCCCATTGATGATGTTTATTTTGATTTTCATTATCAATGCTTCC
ATGGCTTATCAGACAGGTACAGCAATGAATTTGGCTCGTGATCTGGGCCCACGTCTTGCACTATATGC
AGTTGGATTTGATCATAAAATGCTTTGGGTGCATCATCATCATTTCTTTTGGGTTCCCATGGTAGGCC
CATTTATTGGTGCGTTAATGGGGGGGTTGGTTTACGATGTCTGTATTTATCAGGGTCATGAATCTCCA
GTCAACTGGTCTTTACCAGTTTATAAGGAAATGATTATGAGAGCCTGGTTTAGAAGGCCTGGTTGGAA
GAAGAGAAATAGAGCAAGAAGAACATCGGACCTGAGTGACTTCTCATACAATAACGATGATGATGAGG
AATTTGGAGAAAGAATGGCTCTTCAAAAGACAAAGACCAAGTCATCTATTTCAGACAACGAAAATGAA
GCAGGAGAAAAGAAAGTGCAATTTAAATCTGTTCAGCGCGGCAAAAGAACGTTTGGTGGTATACCAAC
AATTCTTGAAGAAGAAGATTCCATTGAAACTGCTTCGCTAGGTGCGACGACGACTGATTCTATTGGGT
TATCCGACACATCATCAGAAGATTCGCATTATGGTAATGCTAAGAAGGTAAGCGGCCGCATCTTTTAC
CCATACGATGTTCCTGACTATGCGGGCTATCCCTATGACGTCCCGGACTATGCAGGATCCTATCCATA
TGACGTTCCAGATTACGCTGCTCAGTGCGGCCGCTGAGAAAACAGACAAGAAAAAGAAGCTTACGCGT
CGACCCGGGTATCCGTATGATGTGCCTGACTACGCATGATATCTCGAGCTCAGCTAGCTAACTGAATA
AGGAACAATGAACGTTTTTCCTTTCTCTTGTTCCTAGTATTAATGACTGACCGATACATCCCTTTTTT
TTTTTGTCTTTGTCTAGCTCCAATTCGCCCTATAGTGAGTCGTATTACAATTCACTGGCCGTCGTTTT
ACAACGTCGTGACTGGGAAAACCCTGGCGTTACCCAACTTAATCGCCTTGCAGCACATCCCCCTTTCG
CCAGCTGGCGTAATAGCGAAGAGGCCCGCACCGATCGCCCTTCCCAACAGTTGCGCAGCCTGAATGGC
GAATGGACGCGCCCTGTAGCGGCGCATTAAGCGCGGCGGGTGTGGTGGTTACGCGCAGCGTGACCGCT
ACACTTGCCAGCGCCCTAGCGCCCGCTCCTTTCGCTTTCTTCCCTTCCTTTCTCGCCACGTTCGCCGG
CTTTCCCCGTCAAGCTCTAAATCGGGGGCTCCCTTTAGGGTTCCGATTTAGTGCTTTACGGCACCTCG
ACCCCAAAAAACTTGATTAGGGTGATGGTTCACGTAGTGGGCCATCGCCCTGATAGACGGTTTTTCGC
CCTTTGACGTTGGAGTCCACGTTCTTTAATAGTGGACTCTTGTTCCAAACTGGAACAACACTCAACCC
TATCTCGGTCTATTCTTTTGATTTATAAGGGATTTTGCCGATTTCGGCCTATTGGTTAAAAAATGAGC
TGATTTAACAAAAATTTAACGCGAATTTTAACAAAATATTAACGCTTACAATTTCCTGATGCGGTATT
TTCTCCTTACGCATCTGTGCGGTATTTCACACCGCATAGATCCGTCGAGTTCAAGAGAAAAAAAAAGA
AAAAGCAAAAAGAAAAAAGGAAAGCGCGCCTCGTTCAGAATGACACGTATAGAATGATGCATTACCTT
GTCATCTTCAGTATCATACTGTTCGTATACATACTTACTGACATTCATAGGTATACATATATACACAT
GTATATATATCGTATGCTGCAGCTTTAAATAATCGGTGTCACTACATAAGAACACCTTTGGTGGAGGG
AACATCGTTGGTTCCATTGGGCGAGGTGGCTTCTCTTATGGCAACCGCAAGAGCCTTGAACGCACTCT
CACTACGGTGATGATCATTCTTGCCTCGCAGACAATCAACGTGGAGGGTAATTCTGCTTGCCTCTGCA
AAACTTTCAAGAAAATGCGGGATCATCTCGCAAGAGAGATCTCCTACTTTCTCCCTCTGCAAACCAAG
TTCGACAACTGCGTACGGCCTGTTCGAAAGATCTACCACCGCTCTGGAAAGTGCCTCATCCAAAGGCG
CAAATCCTGATCCAAACCTTTTTACTCCACGCACGGCCCCTAGGGCCTCTTTAAATGCTTGACCGAGA
GCAATCCCGCAGTCTTCAGTGGTGTGATGGTCGTCTATGTGTAAGTCACCAATGCACTCAACGATTAG
CGACCAGCCGGAATGCTTGGCCAGAGCATGTATCATATGGTCCAGAAACCCTATACCTGTGTGGACGT
TAATCACTTGCGATTGTGTGGCCTGTTCTGCTACTGCTTCTGCCTCTTTTTCTGGGAAGATCGAGTGC
TCTATCGCTAGGGGACCACCCTTTAAAGAGATCGCAATCTGAATCTTGGTTTCATTTGTAATACGCTT
TACTAGGGCTTTCTGCTCTGTCATCTTTGCCTTCGTTTATCTTGCCTGCTCATTTTTTAGTATATTCT
TCGAAGAAATCACATTACTTTATATAATGTATAATTCATTATGTGATAATGCCAATCGCTAAGAAAAA
AAAAGAGTCATCCGCTAGGGGAAAAAAAAAAAATGAAAATCATTACCGAGGCATAAAAAAATATAGAGT
GTACTAGAGGAGGCCAAGAGTAATAGAAAAAGAAAATTGCGGGAAAGGACTGTGTTATGACTTCCCTG
ACTAATGCCGTGTTCAAACGATACCTGGCAGTGACTCCTAGCGCTCACCAAGCTCTTAAAACGGGAAT
TTATGGTGCACTCTCAGTACAATCTGCTCTGATGCCGCATAGTTAAGCCAGCCCCGACACCCGCCAAC
ACCCGCTGACGCGCCCTGACGGGCTTGTCTGCTCCCGGCATCCGCTTACAGACAAGCTGTGACCGTCT
CCGGGAGCTGCATGTGTCAGAGGTTTTCACCGTCATCACCGAAACGCGCGAGACGAAAGGGCCTCGTG
ATACGCCTATTTTTATAGGTTAATGTCATGATAATAATGGTTTCTTAGACGTGCGGCCGCTCTAGAAC

TAGTGGATCAATTCCACGGACTATAGACTATACTAGTATACTCCGTCTACTGTACGATACACTTCCGC
TCAGGTCCTTGTCCTTTAACGAGGCCTTACCACTCTTTTGTTACTCTATTGATCCAGCTCAGCAAAGG
CAGTGTGATCTAAGATTCTATCTTCGCGATGTAGTAAAACTAGCTAGACCGAGAAAGAGACTAGAAAT
GCAAAAGGCACTTCTACAATGGCTGCCATCATTATTATCCGATGTGACGCTGCAGCTTCTCAATGATA
TTCGAATACGCTTTGAGGAGATACAGCCTAATATCCGACAAACTGTTTTACAGATTTACGATCGTACT
TGTTACCCATCATTGAATTTTGAACATCCGAACCTGGGAGTTTTCCCTGAAACAGATAGTATATTTGA
ACCTGTATAATAATATATAGTCTAGCGCTTTACGGAAGACAATGTATGTATTTCGGTTCCTGGAGAAA
CTATTGCATCTATTGCATAGGTAATCTTGCACGTCGCATCCCCGGTTCATTTTCTGCGTTTCCATCTT
GCACTTCAATAGCATATCTTTGTTAACGAAGCATCTGTGCTTCATTTTGTAGAACAAAAATGCAACGC
GAGAGCGCTAATTTTTCAAACAAAGAATCTGAGCTGCATTTTTACAGAACAGAAATGCAACGCGAAAG
CGCTATTTTACCAACGAAGAATCTGTGCTTCATTTTTGTAAAACAAAAATGCAACGCGAGAGCGCTAA
TTTTTCAAACAAAGAATCTGAGCTGCATTTTTACAGAACAGAAATGCAACGCGAGAGCGCTATTTTAC
CAACAAAGAATCTATACTTCTTTTTTGTTCTACAAAAATGCATCCCGAGAGCGCTATTTTTCTAACAA
AGCATCTTAGATTACTTTTTTTCTCCTTTGTGCGCTCTATAATGCAGTCTCTTGATAACTTTTTGCAC
TGTAGGTCCGTTAAGGTTAGAAGAAGGCTACTTTGGTGTCTATTTTCTCTTCCATAAAAAAAGCCTGA
CTCCACTTCCCGCGTTTACTGATTACTAGCGAAGCTGCGGGTGCATTTTTTCAAGATAAAGGCATCCC
CGATTATATTCTATACCGATGTGGATTGCGCATACTTTGTGAACAGAAAGTGATAGCGTTGATGATTC
TTCATTGGTCAGAAAATTATGAACGGTTTCTTCTATTTTGTCTCTATATACTACGTATAGGAAATGTT
TACATTTTCGTATTGTTTTCGATTCACTCTATGAATAGTTCTTACTACAATTTTTTTGTCTAAAGAGT
AATACTAGAGATAAACATAAAAAATGTAGAGGTCGAGTTTAGATGCAAGTTCAAGGAGCGAAAGGTGG
ATGGGTAGGTTATATAGGGATATAGCACAGAGATATATAGCAAAGAGATACTTTTGAGCAATGTTTGT
GGAAGCGGTATTCGCAATATTTTAGTAGCTCGTTACAGTCCGGTGCGTTTTTGGTTTTTTGAAAGTGC
GTCTTCAGAGCGCTTTTGGTTTTCAAAAGCGCTCTGAAGTTCCTATACTTTCTAGAGAATAGGAACTT
CGGAATAGGAACTTCAAAGCGTTTCCGAAAACGAGCGCTTCCGAAAATGCAACGCGAGCTGCGCACAT
ACAGCTCACTGTTCACGTCGCACCTATATCTGCGTGTTGCCTGTATATATATATACATGAGAAGAACG
GCATAGTGCGTGTTTATGCTTAAATGCGTACTTATATGCGTCTATTTATGTAGGATGAAAGGTAGTCT
AGTACCTCCTGTGATATTATCCCATTCCATGCGGGGTATCGTATGCTTCCTTCAGCACTACCCTTTAG
CTGTTCTATATGCTGCCACTCCTCAATTGGATTAGTCTCATCCTTCAATGCTATCATTTCCTTTGATA
TTGGATCATATGCATAGTACCGAGAAACTAGTGCGAAGTAGTGATCAGGTATTGCTGTTATCTGATGA
GTATACGTTGTCCTGGCCACGGCAGAAGCACGCTTATCGCTCCAATTTCCCACAACATTAGTCAACTC
CGTTAGGCCCTTCATTGAAAGAAATGAGGTCATCAAATGTCTTCCAATGTGAGATTTTGGGCCATTTT
TTATAGCAAAGATTGAATAAGGCGCATTTTTCTTCAAAGCTGCGGCCGCACGTCAGGTGGCACTTTTC
GGGGAAATGTGCGCGGAACCCCTATTTGTTTATTTTTCTAAATACATTCAAATATGTATCCGCTCATG
AGACAATAACCGTGATAAATGCTTCAATAATATTGAAAAAGGAAGAGTATGAGTATTCAACATTTCCG
TGTCGCCCTTATTCCCTTTTTTGCGGCATTTTGCCTTCCTGTTTTTGCTCACCCAGAAACGCTGGTGA
AAGTAAAAGATGCTGAAGATCAGTTGGGTGCACGAGTGGGTTACATCGAACTGGATCTCAACAGCGGT
AAGATCCTTGAGAGTTTTCGCCCCGAAGAACGTTTTCCAATGATGAGCACTTTTAAAGTTCTGCTATG
TGGCGCGGTATTATCCCGTATTGACGCCGGGCAAGAGCAACTCGGTCGCCGCATACACTATTCTCAGA
ATGACTTGGTTGAGTACTCACCAGTCACAGAAAAGCATCTTACGGATGGCATGACAGTAAGAGAATTA
TGCAGTGCTGCCATAACCATGAGTGATAACACTGCGGCCAACTTACTTCTGACAACGATCGGAGGACC
GAAGGAGCTAACCGCTTTTTTGCACAACATGGGGGATCATGTAACTCGCCTTGATCGTTGGGAACCGG
AGCTGAATGAAGCCATACCAAACGACGAGCGTGACACCACGATGCCTGTAGCAATGGCAACAACGTTG
CGCAAACTATTAACTGGCGAACTACTTACTCTAGCTTCCCGGCAACAATTAATAGACTGGATGGAGGC
GGATAAAGTTGCAGGACCACTTCTGCGCTCGGCCCTTCCGGCTGGCTGGTTTATTGCTGATAAATCTG
GAGCCGGTGAGCGTGGGTCTCGCGGTATCATTGCAGCACTGGGGCCAGATGGTAAGCCCTCCCGTATC
GTAGTTATCTACACGACGGGGAGTCAGGCAACTATGGATGAACGAAATAGACAGATCGCTGAGATAGG
TGCCTCACTGATTAAGCATTGGTAACTGTCAGACCAAGTTTACTCATATATACTTTAGATTGATTTAA
AACTTCATTTTTAATTTAAAAGGATCTAGGTGAAGATCCTTTTTGATAATCTCATGACCAAAATCCCT

TAACGTGAGTTTTCGTTCCACTGAGCGTCAGACCCCGTAGAAAAGATCAAAGGATCTTCTTGAGATCC
TTTTTTTCTGCGCGTAATCTGCTGCTTGCAAACAAAAAAACCACCGCTACCAGCGGTGGTTTGTTTGC
CGGATCAAGAGCTACCAACTCTTTTTCCGAAGGTAACTGGCTTCAGCAGAGCGCAGATACCAAATACT
GTTCTTCTAGTGTAGCCGTAGTTAGGCCACCACTTCAAGAACTCTGTAGCACCGCCTACATACCTCGC
TCTGCTAATCCTGTTACCAGTGGCTGCTGCCAGTGGCGATAAGTCGTGTCTTACCGGGTTGGACTCAA
GACGATAGTTACCGGATAAGGCGCAGCGGTCGGGCTGAACGGGGGGTTCGTGCACACAGCCCAGCTTG
GAGCGAACGACCTACACCGAACTGAGATACCTACAGCGTGAGCTATGAGAAAGCGCCACGCTTCCCGA
AGGGAGAAAGGCGGACAGGTATCCGGTAAGCGGCAGGGTCGGAACAGGAGAGCGCACGAGGGAGCTTC
CAGGGGGAAACGCCTGGTATCTTTATAGTCCTGTCGGGTTTCGCCACCTCTGACTTGAGCGTCGATTT
TTGTGATGCTCGTCAGGGGGGCGGAGCCTATGGAAAAACGCCAGCAACGCGGCCTTTTTACGGTTCCT
GGCCTTTTGCTGGCCTTTTGCTCACATGTTCTTTCCTGCGTTATCCCCTGATTCTGTGGATAACCGTA
TTACCGCCTTTGAGTGAGCTGATACCGCTCGCCGCAGCCGAACGACCGAGCGCAGCGAGTCAGTGAGC
GAGGAAGCGGAAGAGCGCCCAATACGCAAACCGCCTCTCCCCGCGCGTTGGCCGATTCATTAATGCAG
CTGGCACGACAGGTTTCCCGACTGGAAAGCGGGCAGTGAGCGCAACGCAATTAATGTGAGTTAGCTCA
CTCATTAGGCACCCCAGGCTTTACACTTTATGCTTCCGGCTCGTATGTTGTGTGGAATTGTGAGCGGA
TAACAATTTCACACAGGAAACAGCTATGACCATGATTACGCCAAGCTCGAAATTAACCCTCACTAAAG
GGAACAAAAGCTGGTACCGGGCCGGCCGTCGGGCCGTCGAGCTTGATGGCATCGTGGTGTCACGCTCG
TCGTTTGGTATGGCTTCATTCAGCTCCGGTTCCCAACGATCAAGGCGAGTTACATGATCCCCCATGTT
GTGCAAAAAAGCGGTTAGCTCCTTCGGTCCTCCGATCGTTGTCAGAAGTAAGTTGGCCGCAGTGTTAT
CACTCATGGTTATGGCAGCACTGCATAATTCTCTTACTGTCATGCCATCCGTAAGATGCTTTTCTGTG
ACTGGTGAGTACTCAACCAAGTCATTCTGAGAATAGTGTATGCGGCGACCGAGTTGCTCTTGCCCGGC
GTCAACACGGGATAATACCGCGCCACATAGCAGAACTTTAAAAGTGCTCATCATTGGAAAACGTTCTT
CGGGGCGAAAACTCTCAAGGATCTTACCGCTGTTGAGATCCAGTTCGATGTAACCCACTCGTGCACCC
AACTGATCTTCAGCATCTTTTACTTTCACCAGCGTTTCTGGGTGAGCAAAAACAGGAAGGCAAAATGC
CGCAAAAAAGGGAATAAGGGCGACACGGAAATGTTGAATACTCATACTCTTCCTTTTTCAATATTATT
GAAGCATTTATCAGGGTTATTGTCTCATGAGCGGATACATATTTGAATGTATTTAGAAAAATAAACAA
ATAGGGGTTCCGCGCACATTTCCCCGAAAAGTGCCACCTGACGTCTAAGAAACCATTATTATCATGAC
ATTAACCTATAAAAATAGGCGTATCACGAGGCCCTTTCGTCTTCAAGAATTGGGGATCTACGTATGGT
CATTTCTTCTTCAGATTCCCTCATGGAGAAAGTGCGGCAGATGTATATGACAGAGTCGCCAGTTTCCA
AGAGACTTTATTCAGGCACTTCCATGATAGGCAAGAGAGAAGACCCAGAGATGTTGTTGTCCTAGTTA
CACATGGTATTTATTCCAGAGTATTCCTGATGAAATGGTTTAGATGGACATACGAAGAGTTTGAATCG
TTTACCAATGTTCCTAACGGGAGCGTAATGGTGATGGAACTGGACGAATCCATCAATAGATACGTCCT
GAGGACCGTGCTACCCAAATGGACTGATTGTGAGGGAGACCTAACTACATAGTGTTTAAAGATTACGG
ATATTTAACTTACTTAGAATAATGCCATTTTTTTGAGTTATAATAATCCTACGTTAGTGTGAGCGGGA
TTTAAACTGTGAGGACCTTAATACATTCAGACACTTCTGCGGTATCACCCTACTTATTCCCTTCGAGA
TTATATCTAGGAACCCATCAGGTTGGTGGAAGATTACCCGTTCTAAGACTTTTCAGCTTCCTCTATTG
ATGTTACACCTGGACACCCCTTTTCTGGCATCCAGTTTTTAATCTTCAGTGGCATGTGAGATTCTCCG
AAATTAATTAAAGCAATCACACAATTCTCTCGGATACCACCTCGGTTGAAACTGACAGGTGGTTTGTT
ACGCATGCTAATGCAAAGGAGCCTATATACCTTTGGCTCGGCTGCTGTAACAGGGAATATAAAGGGCA
GCATAATTTAGGAGTTTAGTGAACTTGCAACATTTACTATTTTCCCTTCTTACGTAAATATTTTTCTT
TTTAATTCTAAATCAATCTTTTTCAATTTTTTGTTTGTATTCTTTTCTTGCTTAAATCTATAACTACA
AAAAACACATACAG

Figure S4: Vector map and nucleotide sequence of expression plasmid pYX222-5´Δ1-43-uORF-stop-removed-*FPS1*-HA$_3$

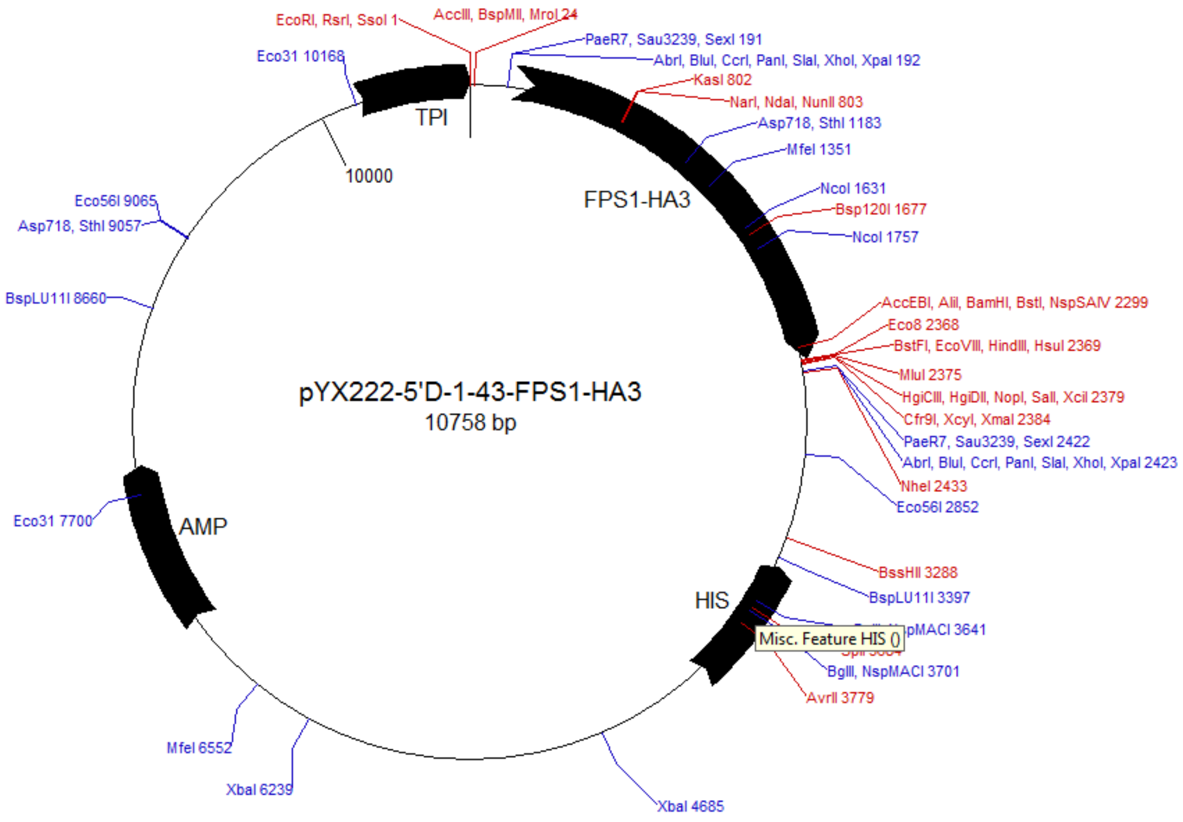

GAATTCGCGGGGAACAGTGTGAATCCGGAGACGGCAAGATTGCCCGGCCCTTTTTGCGGAAAAGATAA
AACAAGATATATTGCACTTTTTCCACCAAGAAAAACAGGAAGTGGATTAAAAAATCAACAAAGTATAA
CGCCTATTGTCCCAATAAGCGTCGGTTGTTCTTCTTTATTATTTTACCAAGTACGCTCGAGGGTACAT
TCTAATGCACCTTAAAAGACATGAGTAATCCTCAAAAAGCTCTAAACGACTTTCTGTCCAGTGAATCT
GTTCATACACATGATAGTTCTAGGAAACAATCTAATAAGCAGTCATCCGACGAAGGACGCTCTTCATC
ACAACCTTCACATCATCACTCTGGTGGTACTAACAACAATAATAACAATAATAATAATAATAATAACA
GTAACAACAACAACAACGGCAACGATGGGGGAAATGATGACGACTATGATTATGAAATGCAAGATTAT
AGACCTTCTCCGCAAAGTGCGCGGCCTACTCCCACGTATGTTCCACAATATTCTGTAGAAAGTGGGAC
TGCTTTCCCGATTCAAGAGGTTATTCCTAGCGCATACATTAACACACAAGATATAAACCATAAAGATA
ACGGTCCGCCGAGTGCAAGCAGTAATAGAGCATTCAGGCCTAGAGGGCAGACCACAGTGTCGGCCAAC
GTGCTTAACATTGAAGATTTTTACAAAAATGCAGACGATGCGCATACCATCCCGGAGTCACATTTATC
GAGAAGGAGAAGTAGGTCGAGGGCTACGAGTAATGCTGGGCACAGTGCCAATACAGGCGCCACGAATG
GCAGGACTACTGGTGCCCAAACTAATATGGAAAGCAATGAATCACCACGTAACGTCCCCATTATGGTG
AAGCCAAAGACATTATACCAGAACCCTCAAACACCTACAGTCTTGCCCTCCACATACCATCCAATTAA
TAAATGGTCTTCCGTCAAAAACACTTATTTGAAGGAATTTTTAGCCGAGTTTATGGGAACAATGGTTA
TGATTATTTTCGGTAGTGCTGTTGTTTGTCAGGTCAATGTTGCTGGGAAAATACAGCAGGACAATTTC
AACGTGGCTTTGGATAACCTTAACGTTACCGGGTCTTCTGCAGAAACGATAGACGCTATGAAGAGTTT
AACATCCTTGGTTTCATCCGTTGCGGGCGGTACCTTTGATGATGTGGCATTGGGCTGGGCTGCTGCCG
TGGTGATGGGCTATTTCTGCGCTGGTGGTAGTGCCATCTCAGGTGCTCATTTGAATCCGTCTATTACA
TTAGCCAATTTGGTGTATAGAGGTTTTCCCCTGAAGAAAGTTCCTTATTACTTTGCTGGACAATTGAT
CGGTGCCTTCACAGGCGCTTTGATCTTGTTTATTTGGTACAAAAGGGTGTTACAAGAGGCATATAGCG

ATTGGTGGATGAATGAAAGTGTTGCGGGAATGTTTTGCGTTTTTCCAAAGCCTTATCTAAGTTCAGGA
CGGCAATTTTTTTCCGAATTTTTATGTGGAGCTATGTTACAAGCAGGAACATTTGCGCTGACCGATCC
TTATACGTGTTTGTCCTCTGATGTTTTCCCATTGATGATGTTTATTTTGATTTTCATTATCAATGCTT
CCATGGCTTATCAGACAGGTACAGCAATGAATTTGGCTCGTGATCTGGGCCCACGTCTTGCACTATAT
GCAGTTGGATTTGATCATAAAATGCTTTGGGTGCATCATCATCATTTCTTTTGGGTTCCCATGGTAGG
CCCATTTATTGGTGCGTTAATGGGGGGGTTGGTTTACGATGTCTGTATTTATCAGGGTCATGAATCTC
CAGTCAACTGGTCTTTACCAGTTTATAAGGAAATGATTATGAGAGCCTGGTTTAGAAGGCCTGGTTGG
AAGAAGAGAAATAGAGCAAGAAGAACATCGGACCTGAGTGACTTCTCATACAATAACGATGATGATGA
GGAATTTGGAGAAAGAATGGCTCTTCAAAAGACAAAGACCAAGTCATCTATTTCAGACAACGAAAATG
AAGCAGGAGAAAAGAAAGTGCAATTTAAATCTGTTCAGCGCGGCAAAAGAACGTTTGGTGGTATACCA
ACAATTCTTGAAGAAGAAGATTCCATTGAAACTGCTTCGCTAGGTGCGACGACGACTGATTCTATTGG
GTTATCCGACACATCATCAGAAGATTCGCATTATGGTAATGCTAAGAAGGTAAGCGGCCGCATCTTTT
ACCCATACGATGTTCCTGACTATGCGGGCTATCCCTATGACGTCCCGGACTATGCAGGATCCTATCCA
TATGACGTTCCAGATTACGCTGCTCAGTGCGGCCGCTGAGAAAACAGACAAGAAAAAGAAGCTTACGC
GTCGACCCGGGTATCCGTATGATGTGCCTGACTACGCATGATATCTCGAGCTCAGCTAGCTAACTGAA
TAAGGAACAATGAACGTTTTTCCTTTCTCTTGTTCCTAGTATTAATGACTGACCGATACATCCCTTTT
TTTTTTTGTCTTTGTCTAGCTCCAATTCGCCCTATAGTGAGTCGTATTACAATTCACTGGCCGTCGTT
TTACAACGTCGTGACTGGGAAAACCCTGGCGTTACCCAACTTAATCGCCTTGCAGCACATCCCCCTTT
CGCCAGCTGGCGTAATAGCGAAGAGGCCCGCACCGATCGCCCTTCCCAACAGTTGCGCAGCCTGAATG
GCGAATGGACGCGCCCTGTAGCGGCGCATTAAGCGCGGCGGGTGTGGTGGTTACGCGCAGCGTGACCG
CTACACTTGCCAGCGCCCTAGCGCCCGCTCCTTTCGCTTTCTTCCCTTCCTTTCTCGCCACGTTCGCC
GGCTTTCCCCGTCAAGCTCTAAATCGGGGGCTCCCTTTAGGGTTCCGATTTAGTGCTTTACGGCACCT
CGACCCCAAAAAACTTGATTAGGGTGATGGTTCACGTAGTGGGCCATCGCCCTGATAGACGGTTTTTC
GCCCTTTGACGTTGGAGTCCACGTTCTTTAATAGTGGACTCTTGTTCCAAACTGGAACAACACTCAAC
CCTATCTCGGTCTATTCTTTTGATTTATAAGGGATTTTGCCGATTTCGGCCTATTGGTTAAAAAATGA
GCTGATTTAACAAAAATTTAACGCGAATTTTAACAAAATATTAACGCTTACAATTTCCTGATGCGGTA
TTTTCTCCTTACGCATCTGTGCGGTATTTCACACCGCATAGATCCGTCGAGTTCAAGAGAAAAAAAAA
GAAAAAGCAAAAAGAAAAAAGGAAAGCGCGCCTCGTTCAGAATGACACGTATAGAATGATGCATTACC
TTGTCATCTTCAGTATCATACTGTTCGTATACATACTTACTGACATTCATAGGTATACATATATACAC
ATGTATATATATCGTATGCTGCAGCTTTAAATAATCGGTGTCACTACATAAGAACACCTTTGGTGGAG
GGAACATCGTTGGTTCCATTGGGCGAGGTGGCTTCTCTTATGGCAACCGCAAGAGCCTTGAACGCACT
CTCACTACGGTGATGATCATTCTTGCCTCGCAGACAATCAACGTGGAGGGTAATTCTGCTTGCCTCTG
CAAAACTTTCAAGAAAATGCGGGATCATCTCGCAAGAGAGATCTCCTACTTTCTCCCTCTGCAAACCA
AGTTCGACAACTGCGTACGGCCTGTTCGAAAGATCTACCACCGCTCTGGAAAGTGCCTCATCCAAAGG
CGCAAATCCTGATCCAAACCTTTTTACTCCACGCACGGCCCCTAGGGCCTCTTTAAATGCTTGACCGA
GAGCAATCCCGCAGTCTTCAGTGGTGTGATGGTCGTCTATGTGTAAGTCACCAATGCACTCAACGATT
AGCGACCAGCCGGAATGCTTGGCCAGAGCATGTATCATATGGTCCAGAAACCCTATACCTGTGTGGAC
GTTAATCACTTGCGATTGTGTGGCCTGTTCTGCTACTGCTTCTGCCTCTTTTTCTGGGAAGATCGAGT
GCTCTATCGCTAGGGGACCACCCTTTAAAGAGATCGCAATCTGAATCTTGGTTTCATTTGTAATACGC
TTTACTAGGGCTTTCTGCTCTGTCATCTTTGCCTTCGTTTATCTTGCCTGCTCATTTTTTAGTATATT
CTTCGAAGAAATCACATTACTTTATATAATGTATAATTCATTATGTGATAATGCCAATCGCTAAGAAA
AAAAAAGAGTCATCCGCTAGGGGAAAAAAAAAAAATGAAAATCATTACCGAGGCATAAAAAAATATAGA
GTGTACTAGAGGAGGCCAAGAGTAATAGAAAAAGAAAATTGCGGGAAAGGACTGTGTTATGACTTCCC
TGACTAATGCCGTGTTCAAACGATACCTGGCAGTGACTCCTAGCGCTCACCAAGCTCTTAAAACGGGA
ATTTATGGTGCACTCTCAGTACAATCTGCTCTGATGCCGCATAGTTAAGCCAGCCCCGACACCCGCCA
ACACCCGCTGACGCGCCCTGACGGGCTTGTCTGCTCCCGGCATCCGCTTACAGACAAGCTGTGACCGT
CTCCGGGAGCTGCATGTGTCAGAGGTTTTCACCGTCATCACCGAAACGCGCGAGACGAAAGGGCCTCG
TGATACGCCTATTTTTATAGGTTAATGTCATGATAATAATGGTTTCTTAGACGTGCGGCCGCTCTAGA

ACTAGTGGATCAATTCCACGGACTATAGACTATACTAGTATACTCCGTCTACTGTACGATACACTTCC
GCTCAGGTCCTTGTCCTTTAACGAGGCCTTACCACTCTTTTGTTACTCTATTGATCCAGCTCAGCAAA
GGCAGTGTGATCTAAGATTCTATCTTCGCGATGTAGTAAAACTAGCTAGACCGAGAAAGAGACTAGAA
ATGCAAAAGGCACTTCTACAATGGCTGCCATCATTATTATCCGATGTGACGCTGCAGCTTCTCAATGA
TATTCGAATACGCTTTGAGGAGATACAGCCTAATATCCGACAAACTGTTTTACAGATTTACGATCGTA
CTTGTTACCCATCATTGAATTTTGAACATCCGAACCTGGGAGTTTTCCCTGAAACAGATAGTATATTT
GAACCTGTATAATAATATATAGTCTAGCGCTTTACGGAAGACAATGTATGTATTTCGGTTCCTGGAGA
AACTATTGCATCTATTGCATAGGTAATCTTGCACGTCGCATCCCCGGTTCATTTTCTGCGTTTCCATC
TTGCACTTCAATAGCATATCTTTGTTAACGAAGCATCTGTGCTTCATTTTGTAGAACAAAAATGCAAC
GCGAGAGCGCTAATTTTTCAAACAAAGAATCTGAGCTGCATTTTTACAGAACAGAAATGCAACGCGAA
AGCGCTATTTTACCAACGAAGAATCTGTGCTTCATTTTTGTAAAACAAAAATGCAACGCGAGAGCGCT
AATTTTTCAAACAAAGAATCTGAGCTGCATTTTTACAGAACAGAAATGCAACGCGAGAGCGCTATTTT
ACCAACAAAGAATCTATACTTCTTTTTTGTTCTACAAAAATGCATCCCGAGAGCGCTATTTTTCTAAC
AAAGCATCTTAGATTACTTTTTTTCTCCTTTGTGCGCTCTATAATGCAGTCTCTTGATAACTTTTTGC
ACTGTAGGTCCGTTAAGGTTAGAAGAAGGCTACTTTGGTGTCTATTTTCTCTTCCATAAAAAAAGCCT
GACTCCACTTCCCGCGTTTACTGATTACTAGCGAAGCTGCGGGTGCATTTTTTCAAGATAAAGGCATC
CCCGATTATATTCTATACCGATGTGGATTGCGCATACTTTGTGAACAGAAAGTGATAGCGTTGATGAT
TCTTCATTGGTCAGAAAATTATGAACGGTTTCTTCTATTTTGTCTCTATATACTACGTATAGGAAATG
TTTACATTTTCGTATTGTTTTCGATTCACTCTATGAATAGTTCTTACTACAATTTTTTTGTCTAAAGA
GTAATACTAGAGATAAACATAAAAAATGTAGAGGTCGAGTTTAGATGCAAGTTCAAGGAGCGAAAGGT
GGATGGGTAGGTTATATAGGGATATAGCACAGAGATATATAGCAAAGAGATACTTTTGAGCAATGTTT
GTGGAAGCGGTATTCGCAATATTTTAGTAGCTCGTTACAGTCCGGTGCGTTTTTGGTTTTTTGAAAGT
GCGTCTTCAGAGCGCTTTTGGTTTTCAAAAGCGCTCTGAAGTTCCTATACTTTCTAGAGAATAGGAAC
TTCGGAATAGGAACTTCAAAGCGTTTCCGAAAACGAGCGCTTCCGAAAATGCAACGCGAGCTGCGCAC
ATACAGCTCACTGTTCACGTCGCACCTATATCTGCGTGTTGCCTGTATATATATATACATGAGAAGAA
CGGCATAGTGCGTGTTTATGCTTAAATGCGTACTTATATGCGTCTATTTATGTAGGATGAAAGGTAGT
CTAGTACCTCCTGTGATATTATCCCATTCCATGCGGGGTATCGTATGCTTCCTTCAGCACTACCCTTT
AGCTGTTCTATATGCTGCCACTCCTCAATTGGATTAGTCTCATCCTTCAATGCTATCATTTCCTTTGA
TATTGGATCATATGCATAGTACCGAGAAACTAGTGCGAAGTAGTGATCAGGTATTGCTGTTATCTGAT
GAGTATACGTTGTCCTGGCCACGGCAGAAGCACGCTTATCGCTCCAATTTCCCACAACATTAGTCAAC
TCCGTTAGGCCCTTCATTGAAAGAAATGAGGTCATCAAATGTCTTCCAATGTGAGATTTTGGGCCATT
TTTTATAGCAAAGATTGAATAAGGCGCATTTTTCTTCAAAGCTGCGGCCGCACGTCAGGTGGCACTTT
TCGGGGAAATGTGCGCGGAACCCCTATTTGTTTATTTTTCTAAATACATTCAAATATGTATCCGCTCA
TGAGACAATAACCGTGATAAATGCTTCAATAATATTGAAAAAGGAAGAGTATGAGTATTCAACATTTC
CGTGTCGCCCTTATTCCCTTTTTTGCGGCATTTTGCCTTCCTGTTTTTGCTCACCCAGAAACGCTGGT
GAAAGTAAAAGATGCTGAAGATCAGTTGGGTGCACGAGTGGGTTACATCGAACTGGATCTCAACAGCG
GTAAGATCCTTGAGAGTTTTCGCCCCGAAGAACGTTTTCCAATGATGAGCACTTTTAAAGTTCTGCTA
TGTGGCGCGGTATTATCCCGTATTGACGCCGGGCAAGAGCAACTCGGTCGCCGCATACACTATTCTCA
GAATGACTTGGTTGAGTACTCACCAGTCACAGAAAAGCATCTTACGGATGGCATGACAGTAAGAGAAT
TATGCAGTGCTGCCATAACCATGAGTGATAACACTGCGGCCAACTTACTTCTGACAACGATCGGAGGA
CCGAAGGAGCTAACCGCTTTTTTGCACAACATGGGGGATCATGTAACTCGCCTTGATCGTTGGGAACC
GGAGCTGAATGAAGCCATACCAAACGACGAGCGTGACACCACGATGCCTGTAGCAATGGCAACAACGT
TGCGCAAACTATTAACTGGCGAACTACTTACTCTAGCTTCCCGGCAACAATTAATAGACTGGATGGAG
GCGGATAAAGTTGCAGGACCACTTCTGCGCTCGGCCCTTCCGGCTGGCTGGTTTATTGCTGATAAATC
TGGAGCCGGTGAGCGTGGGTCTCGCGGTATCATTGCAGCACTGGGGCCAGATGGTAAGCCCTCCCGTA
TCGTAGTTATCTACACGACGGGGAGTCAGGCAACTATGGATGAACGAAATAGACAGATCGCTGAGATA
GGTGCCTCACTGATTAAGCATTGGTAACTGTCAGACCAAGTTTACTCATATATACTTTAGATTGATTT
AAAACTTCATTTTTAATTTAAAAGGATCTAGGTGAAGATCCTTTTTGATAATCTCATGACCAAAATCC

CTTAACGTGAGTTTTCGTTCCACTGAGCGTCAGACCCCGTAGAAAAGATCAAAGGATCTTCTTGAGAT
CCTTTTTTTCTGCGCGTAATCTGCTGCTTGCAAACAAAAAAACCACCGCTACCAGCGGTGGTTTGTTT
GCCGGATCAAGAGCTACCAACTCTTTTTCCGAAGGTAACTGGCTTCAGCAGAGCGCAGATACCAAATA
CTGTTCTTCTAGTGTAGCCGTAGTTAGGCCACCACTTCAAGAACTCTGTAGCACCGCCTACATACCTC
GCTCTGCTAATCCTGTTACCAGTGGCTGCTGCCAGTGGCGATAAGTCGTGTCTTACCGGGTTGGACTC
AAGACGATAGTTACCGGATAAGGCGCAGCGGTCGGGCTGAACGGGGGGTTCGTGCACACAGCCCAGCT
TGGAGCGAACGACCTACACCGAACTGAGATACCTACAGCGTGAGCTATGAGAAAGCGCCACGCTTCCC
GAAGGGAGAAAGGCGGACAGGTATCCGGTAAGCGGCAGGGTCGGAACAGGAGAGCGCACGAGGGAGCT
TCCAGGGGGAAACGCCTGGTATCTTTATAGTCCTGTCGGGTTTCGCCACCTCTGACTTGAGCGTCGAT
TTTTGTGATGCTCGTCAGGGGGGCGGAGCCTATGGAAAAACGCCAGCAACGCGGCCTTTTTACGGTTC
CTGGCCTTTTGCTGGCCTTTTGCTCACATGTTCTTTCCTGCGTTATCCCCTGATTCTGTGGATAACCG
TATTACCGCCTTTGAGTGAGCTGATACCGCTCGCCGCAGCCGAACGACCGAGCGCAGCGAGTCAGTGA
GCGAGGAAGCGGAAGAGCGCCCAATACGCAAACCGCCTCTCCCCGCGCGTTGGCCGATTCATTAATGC
AGCTGGCACGACAGGTTTCCCGACTGGAAAGCGGGCAGTGAGCGCAACGCAATTAATGTGAGTTAGCT
CACTCATTAGGCACCCCAGGCTTTACACTTTATGCTTCCGGCTCGTATGTTGTGTGGAATTGTGAGCG
GATAACAATTTCACACAGGAAACAGCTATGACCATGATTACGCCAAGCTCGAAATTAACCCTCACTAA
AGGGAACAAAAGCTGGTACCGGGCCGGCCGTCGGCCGTCGAGCTTGATGGCATCGTGGTGTCACGCT
CGTCGTTTGGTATGGCTTCATTCAGCTCCGGTTCCCAACGATCAAGGCGAGTTACATGATCCCCCATG
TTGTGCAAAAAAGCGGTTAGCTCCTTCGGTCCTCCGATCGTTGTCAGAAGTAAGTTGGCCGCAGTGTT
ATCACTCATGGTTATGGCAGCACTGCATAATTCTCTTACTGTCATGCCATCCGTAAGATGCTTTTCTG
TGACTGGTGAGTACTCAACCAAGTCATTCTGAGAATAGTGTATGCGGCGACCGAGTTGCTCTTGCCCG
GCGTCAACACGGGATAATACCGCGCCACATAGCAGAACTTTAAAAGTGCTCATCATTGGAAAACGTTC
TTCGGGGCGAAAACTCTCAAGGATCTTACCGCTGTTGAGATCCAGTTCGATGTAACCCACTCGTGCAC
CCAACTGATCTTCAGCATCTTTTACTTTCACCAGCGTTTCTGGGTGAGCAAAAACAGGAAGGCAAAAT
GCCGCAAAAAAGGGAATAAGGGCGACACGGAAATGTTGAATACTCATACTCTTCCTTTTTCAATATTA
TTGAAGCATTTATCAGGGTTATTGTCTCATGAGCGGATACATATTTGAATGTATTTAGAAAAATAAAC
AAATAGGGGTTCCGCGCACATTTCCCCGAAAAGTGCCACCTGACGTCTAAGAAACCATTATTATCATG
ACATTAACCTATAAAAATAGGCGTATCACGAGGCCCTTTCGTCTTCAAGAATTGGGGATCTACGTATG
GTCATTTCTTCTTCAGATTCCCTCATGGAGAAAGTGCGGCAGATGTATATGACAGAGTCGCCAGTTTC
CAAGAGACTTTATTCAGGCACTTCCATGATAGGCAAGAGAGAAGACCCAGAGATGTTGTTGTCCTAGT
TACACATGGTATTTATTCCAGAGTATTCCTGATGAAATGGTTTAGATGGACATACGAAGAGTTTGAAT
CGTTTACCAATGTTCCTAACGGGAGCGTAATGGTGATGGAACTGGACGAATCCATCAATAGATACGTC
CTGAGGACCGTGCTACCCAAATGGACTGATTGTGAGGGAGACCTAACTACATAGTGTTTAAAGATTAC
GGATATTTAACTTACTTAGAATAATGCCATTTTTTTGAGTTATAATAATCCTACGTTAGTGTGAGCGG
GATTTAAACTGTGAGGACCTTAATACATTCAGACACTTCTGCGGTATCACCCTACTTATTCCCTTCGA
GATTATATCTAGGAACCCATCAGGTTGGTGGAAGATTACCCGTTCTAAGACTTTTCAGCTTCCTCTAT
TGATGTTACACCTGGACACCCCTTTTCTGGCATCCAGTTTTTAATCTTCAGTGGCATGTGAGATTCTC
CGAAATTAATTAAAGCAATCACACAATTCTCTCGGATACCACCTCGGTTGAAACTGACAGGTGGTTTG
TTACGCATGCTAATGCAAAGGAGCCTATATACCTTTGGCTCGGCTGCTGTAACAGGGAATATAAAGGG
CAGCATAATTTAGGAGTTTAGTGAACTTGCAACATTTACTATTTTCCCTTCTTACGTAAATATTTTTC
TTTTTAATTCTAAATCAATCTTTTTCAATTTTTTGTTTGTATTCTTTTCTTGCTTAAATCTATAACTA
CAAAAAACACATACAG

Figure S5: Vector map and nucleotide sequence of expression plasmid pYX222-5´Δ1-215-*FPS1*-HA_3

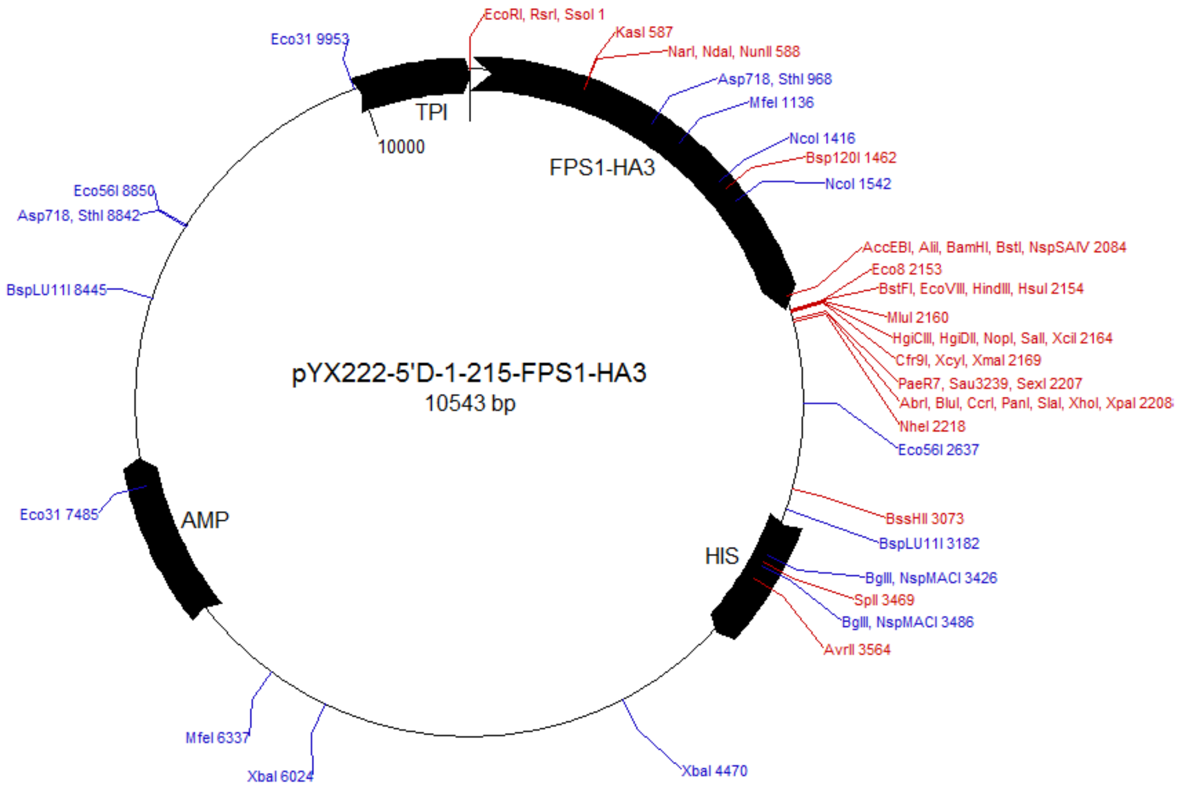

GAATTCCATGAGTAATCCTCAAAAAGCTCTAAACGACTTTCTGTCCAGTGAATCTGTTCATACACATG
ATAGTTCTAGGAAACAATCTAATAAGCAGTCATCCGACGAAGGACGCTCTTCATCACAACCTTCACAT
CATCACTCTGGTGGTACTAACAACAATAATAACAATAATAATAATAATAATAACAGTAACAACAACAA
CAACGGCAACGATGGGGGAAATGATGACGACTATGATTATGAAATGCAAGATTATAGACCTTCTCCGC
AAAGTGCGCGGCCTACTCCCACGTATGTTCCACAATATTCTGTAGAAAGTGGGACTGCTTTCCCGATT
CAAGAGGTTATTCCTAGCGCATACATTAACACACAAGATATAAACCATAAAGATAACGGTCCGCCGAG
TGCAAGCAGTAATAGAGCATTCAGGCCTAGAGGGCAGACCACAGTGTCGGCCAACGTGCTTAACATTG
AAGATTTTTACAAAAATGCAGACGATGCGCATACCATCCCGGAGTCACATTTATCGAGAAGGAGAAGT
AGGTCGAGGGCTACGAGTAATGCTGGGCACAGTGCCAATACAGGCGCCACGAATGGCAGGACTACTGG
TGCCCAAACTAATATGGAAAGCAATGAATCACCACGTAACGTCCCCATTATGGTGAAGCCAAAGACAT
TATACCAGAACCCTCAAACACCTACAGTCTTGCCCTCCACATACCATCCAATTAATAAATGGTCTTCC
GTCAAAAACACTTATTTGAAGGAATTTTTAGCCGAGTTTATGGGAACAATGGTTATGATTATTTTCGG
TAGTGCTGTTGTTTGTCAGGTCAATGTTGCTGGGAAAATACAGCAGGACAATTTCAACGTGGCTTTGG
ATAACCTTAACGTTACCGGGTCTTCTGCAGAAACGATAGACGCTATGAAGAGTTTAACATCCTTGGTT
TCATCCGTTGCGGGCGGTACCTTTGATGATGTGGCATTGGGCTGGGCTGCTGCCGTGGTGATGGGCTA
TTTCTGCGCTGGTGGTAGTGCCATCTCAGGTGCTCATTTGAATCCGTCTATTACATTAGCCAATTTGG
TGTATAGAGGTTTTCCCCTGAAGAAAGTTCCTTATTACTTTGCTGGACAATTGATCGGTGCCTTCACA
GGCGCTTTGATCTTGTTTATTTGGTACAAAAGGGTGTTACAAGAGGCATATAGCGATTGGTGGATGAA
TGAAAGTGTTGCGGGAATGTTTTGCGTTTTTCCAAAGCCTTATCTAAGTTCAGGACGGCAATTTTTTT
CCGAATTTTTATGTGGAGCTATGTTACAAGCAGGAACATTTGCGCTGACCGATCCTTATACGTGTTTG
TCCTCTGATGTTTTCCCATTGATGATGTTTATTTTGATTTTCATTATCAATGCTTCCATGGCTTATCA
GACAGGTACAGCAATGAATTTGGCTCGTGATCTGGGCCCACGTCTTGCACTATATGCAGTTGGATTTG
ATCATAAAATGCTTTGGGTGCATCATCATCATTTCTTTTGGGTTCCCATGGTAGGCCCATTTATTGGT

GCGTTAATGGGGGGGTTGGTTTACGATGTCTGTATTTATCAGGGTCATGAATCTCCAGTCAACTGGTC
TTTACCAGTTTATAAGGAAATGATTATGAGAGCCTGGTTTAGAAGGCCTGGTTGGAAGAAGAGAAATA
GAGCAAGAAGAACATCGGACCTGAGTGACTTCTCATACAATAACGATGATGATGAGGAATTTGGAGAA
AGAATGGCTCTTCAAAAGACAAAGACCAAGTCATCTATTTCAGACAACGAAAATGAAGCAGGAGAAAA
GAAAGTGCAATTTAAATCTGTTCAGCGCGGCAAAAGAACGTTTGGTGGTATACCAACAATTCTTGAAG
AAGAAGATTCCATTGAAACTGCTTCGCTAGGTGCGACGACGACTGATTCTATTGGGTTATCCGACACA
TCATCAGAAGATTCGCATTATGGTAATGCTAAGAAGGTAAGCGGCCGCATCTTTTACCCATACGATGT
TCCTGACTATGCGGGCTATCCCTATGACGTCCCGGACTATGCAGGATCCTATCCATATGACGTTCCAG
ATTACGCTGCTCAGTGCGGCCGCTGAGAAAACAGACAAGAAAAAGAAGCTTACGCGTCGACCCGGGTA
TCCGTATGATGTGCCTGACTACGCATGATATCTCGAGCTCAGCTAGCTAACTGAATAAGGAACAATGA
ACGTTTTTCCTTTCTCTTGTTCCTAGTATTAATGACTGACCGATACATCCCTTTTTTTTTTTGTCTTT
GTCTAGCTCCAATTCGCCCTATAGTGAGTCGTATTACAATTCACTGGCCGTCGTTTTACAACGTCGTG
ACTGGGAAAACCCTGGCGTTACCCAACTTAATCGCCTTGCAGCACATCCCCCTTTCGCCAGCTGGCGT
AATAGCGAAGAGGCCCGCACCGATCGCCCTTCCCAACAGTTGCGCAGCCTGAATGGCGAATGGACGCG
CCCTGTAGCGGCGCATTAAGCGCGGCGGGTGTGGTGGTTACGCGCAGCGTGACCGCTACACTTGCCAG
CGCCCTAGCGCCCGCTCCTTTCGCTTTCTTCCCTTCCTTTCTCGCCACGTTCGCCGGCTTTCCCCGTC
AAGCTCTAAATCGGGGGCTCCCTTTAGGGTTCCGATTTAGTGCTTTACGGCACCTCGACCCCAAAAAA
CTTGATTAGGGTGATGGTTCACGTAGTGGGCCATCGCCCTGATAGACGGTTTTTCGCCCTTTGACGTT
GGAGTCCACGTTCTTTAATAGTGGACTCTTGTTCCAAACTGGAACAACACTCAACCCTATCTCGGTCT
ATTCTTTTGATTTATAAGGGATTTTGCCGATTTCGGCCTATTGGTTAAAAAATGAGCTGATTTAACAA
AAATTTAACGCGAATTTTAACAAAATATTAACGCTTACAATTTCCTGATGCGGTATTTTCTCCTTACG
CATCTGTGCGGTATTTCACACCGCATAGATCCGTCGAGTTCAAGAGAAAAAAAAAGAAAAAGCAAAAA
GAAAAAAGGAAAGCGCGCCTCGTTCAGAATGACACGTATAGAATGATGCATTACCTTGTCATCTTCAG
TATCATACTGTTCGTATACATACTTACTGACATTCATAGGTATACATATATACACATGTATATATATC
GTATGCTGCAGCTTTAAATAATCGGTGTCACTACATAAGAACACCTTTGGTGGAGGGAACATCGTTGG
TTCCATTGGGCGAGGTGGCTTCTCTTATGGCAACCGCAAGAGCCTTGAACGCACTCTCACTACGGTGA
TGATCATTCTTGCCTCGCAGACAATCAACGTGGAGGGTAATTCTGCTTGCCTCTGCAAAACTTTCAAG
AAAATGCGGGATCATCTCGCAAGAGAGATCTCCTACTTTCTCCCTCTGCAAACCAAGTTCGACAACTG
CGTACGGCCTGTTCGAAAGATCTACCACCGCTCTGGAAAGTGCCTCATCCAAAGGCGCAAATCCTGAT
CCAAACCTTTTTACTCCACGCACGGCCCCTAGGGCCTCTTTAAATGCTTGACCGAGAGCAATCCCGCA
GTCTTCAGTGGTGTGATGGTCGTCTATGTGTAAGTCACCAATGCACTCAACGATTAGCGACCAGCCGG
AATGCTTGGCCAGAGCATGTATCATATGGTCCAGAAACCCTATACCTGTGTGGACGTTAATCACTTGC
GATTGTGTGGCCTGTTCTGCTACTGCTTCTGCCTCTTTTTCTGGGAAGATCGAGTGCTCTATCGCTAG
GGGACCACCCTTTAAAGAGATCGCAATCTGAATCTTGGTTTCATTTGTAATACGCTTTACTAGGGCTT
TCTGCTCTGTCATCTTTGCCTTCGTTTATCTTGCCTGCTCATTTTTTAGTATATTCTTCGAAGAAATC
ACATTACTTTATATAATGTATAATTCATTATGTGATAATGCCAATCGCTAAGAAAAAAAAAGAGTCAT
CCGCTAGGGGAAAAAAAAAAATGAAAATCATTACCGAGGCATAAAAAAATATAGAGTGTACTAGAGGA
GGCCAAGAGTAATAGAAAAAGAAAATTGCGGGAAAGGACTGTGTTATGACTTCCCTGACTAATGCCGT
GTTCAAACGATACCTGGCAGTGACTCCTAGCGCTCACCAAGCTCTTAAAACGGGAATTTATGGTGCAC
TCTCAGTACAATCTGCTCTGATGCCGCATAGTTAAGCCAGCCCCGACACCCGCCAACACCCGCTGACG
CGCCCTGACGGGCTTGTCTGCTCCCGGCATCCGCTTACAGACAAGCTGTGACCGTCTCCGGGAGCTGC
ATGTGTCAGAGGTTTTCACCGTCATCACCGAAACGCGCGAGACGAAAGGGCCTCGTGATACGCCTATT
TTTATAGGTTAATGTCATGATAATAATGGTTTCTTAGACGTGCGGCCGCTCTAGAACTAGTGGATCAA
TTCCACGGACTATAGACTATACTAGTATACTCCGTCTACTGTACGATACACTTCCGCTCAGGTCCTTG
TCCTTTAACGAGGCCTTACCACTCTTTTGTTACTCTATTGATCCAGCTCAGCAAAGGCAGTGTGATCT
AAGATTCTATCTTCGCGATGTAGTAAAACTAGCTAGACCGAGAAAGAGACTAGAAATGCAAAAGGCAC
TTCTACAATGGCTGCCATCATTATTATCCGATGTGACGCTGCAGCTTCTCAATGATATTCGAATACGC
TTTGAGGAGATACAGCCTAATATCCGACAAACTGTTTTACAGATTTACGATCGTACTTGTTACCCATC

ATTGAATTTTGAACATCCGAACCTGGGAGTTTTCCCTGAAACAGATAGTATATTTGAACCTGTATAAT
AATATATAGTCTAGCGCTTTACGGAAGACAATGTATGTATTTCGGTTCCTGGAGAAACTATTGCATCT
ATTGCATAGGTAATCTTGCACGTCGCATCCCCGGTTCATTTTCTGCGTTTCCATCTTGCACTTCAATA
GCATATCTTTGTTAACGAAGCATCTGTGCTTCATTTTGTAGAACAAAAATGCAACGCGAGAGCGCTAA
TTTTTCAAACAAAGAATCTGAGCTGCATTTTTACAGAACAGAAATGCAACGCGAAAGCGCTATTTTAC
CAACGAAGAATCTGTGCTTCATTTTTGTAAAACAAAAATGCAACGCGAGAGCGCTAATTTTTCAAACA
AAGAATCTGAGCTGCATTTTTACAGAACAGAAATGCAACGCGAGAGCGCTATTTTACCAACAAAGAAT
CTATACTTCTTTTTTGTTCTACAAAAATGCATCCCGAGAGCGCTATTTTTCTAACAAAGCATCTTAGA
TTACTTTTTTTCTCCTTTGTGCGCTCTATAATGCAGTCTCTTGATAACTTTTTGCACTGTAGGTCCGT
TAAGGTTAGAAGAAGGCTACTTTGGTGTCTATTTTCTCTTCCATAAAAAAAGCCTGACTCCACTTCCC
GCGTTTACTGATTACTAGCGAAGCTGCGGGTGCATTTTTTCAAGATAAAGGCATCCCCGATTATATTC
TATACCGATGTGGATTGCGCATACTTTGTGAACAGAAAGTGATAGCGTTGATGATTCTTCATTGGTCA
GAAAATTATGAACGGTTTCTTCTATTTTGTCTCTATATACTACGTATAGGAAATGTTTACATTTTCGT
ATTGTTTTCGATTCACTCTATGAATAGTTCTTACTACAATTTTTTTGTCTAAAGAGTAATACTAGAGA
TAAACATAAAAAATGTAGAGGTCGAGTTTAGATGCAAGTTCAAGGAGCGAAAGGTGGATGGGTAGGTT
ATATAGGGATATAGCACAGAGATATATAGCAAAGAGATACTTTTGAGCAATGTTTGTGGAAGCGGTAT
TCGCAATATTTTAGTAGCTCGTTACAGTCCGGTGCGTTTTTGGTTTTTTGAAAGTGCGTCTTCAGAGC
GCTTTTGGTTTTCAAAAGCGCTCTGAAGTTCCTATACTTTCTAGAGAATAGGAACTTCGGAATAGGAA
CTTCAAAGCGTTTCCGAAAACGAGCGCTTCCGAAAATGCAACGCGAGCTGCGCACATACAGCTCACTG
TTCACGTCGCACCTATATCTGCGTGTTGCCTGTATATATATATACATGAGAAGAACGGCATAGTGCGT
GTTTATGCTTAAATGCGTACTTATATGCGTCTATTTATGTAGGATGAAAGGTAGTCTAGTACCTCCTG
TGATATTATCCCATTCCATGCGGGGTATCGTATGCTTCCTTCAGCACTACCCTTTAGCTGTTCTATAT
GCTGCCACTCCTCAATTGGATTAGTCTCATCCTTCAATGCTATCATTTCCTTTGATATTGGATCATAT
GCATAGTACCGAGAAACTAGTGCGAAGTAGTGATCAGGTATTGCTGTTATCTGATGAGTATACGTTGT
CCTGGCCACGGCAGAAGCACGCTTATCGCTCCAATTTCCCACAACATTAGTCAACTCCGTTAGGCCCT
TCATTGAAAGAAATGAGGTCATCAAATGTCTTCCAATGTGAGATTTTGGGCCATTTTTTATAGCAAAG
ATTGAATAAGGCGCATTTTTCTTCAAAGCTGCGGCCGCACGTCAGGTGGCACTTTTCGGGGAAATGTG
CGCGGAACCCCTATTTGTTTATTTTTCTAAATACATTCAAATATGTATCCGCTCATGAGACAATAACC
GTGATAAATGCTTCAATAATATTGAAAAAGGAAGAGTATGAGTATTCAACATTTCCGTGTCGCCCTTA
TTCCCTTTTTTGCGGCATTTTGCCTTCCTGTTTTTGCTCACCCAGAAACGCTGGTGAAAGTAAAAGAT
GCTGAAGATCAGTTGGGTGCACGAGTGGGTTACATCGAACTGGATCTCAACAGCGGTAAGATCCTTGA
GAGTTTTCGCCCCGAAGAACGTTTTCCAATGATGAGCACTTTTAAAGTTCTGCTATGTGGCGCGGTAT
TATCCCGTATTGACGCCGGGCAAGAGCAACTCGGTCGCCGCATACACTATTCTCAGAATGACTTGGTT
GAGTACTCACCAGTCACAGAAAAGCATCTTACGGATGGCATGACAGTAAGAGAATTATGCAGTGCTGC
CATAACCATGAGTGATAACACTGCGGCCAACTTACTTCTGACAACGATCGGAGGACCGAAGGAGCTAA
CCGCTTTTTTGCACAACATGGGGGATCATGTAACTCGCCTTGATCGTTGGGAACCGGAGCTGAATGAA
GCCATACCAAACGACGAGCGTGACACCACGATGCCTGTAGCAATGGCAACAACGTTGCGCAAACTATT
AACTGGCGAACTACTTACTCTAGCTTCCCGGCAACAATTAATAGACTGGATGGAGGCGGATAAAGTTG
CAGGACCACTTCTGCGCTCGGCCCTTCCGGCTGGCTGGTTTATTGCTGATAAATCTGGAGCCGGTGAG
CGTGGGTCTCGCGGTATCATTGCAGCACTGGGGCCAGATGGTAAGCCCTCCCGTATCGTAGTTATCTA
CACGACGGGGAGTCAGGCAACTATGGATGAACGAAATAGACAGATCGCTGAGATAGGTGCCTCACTGA
TTAAGCATTGGTAACTGTCAGACCAAGTTTACTCATATATACTTTAGATTGATTTAAAACTTCATTTT
TAATTTAAAAGGATCTAGGTGAAGATCCTTTTTGATAATCTCATGACCAAAATCCCTTAACGTGAGTT
TTCGTTCCACTGAGCGTCAGACCCCGTAGAAAAGATCAAAGGATCTTCTTGAGATCCTTTTTTTCTGC
GCGTAATCTGCTGCTTGCAAACAAAAAAACCACCGCTACCAGCGGTGGTTTGTTTGCCGGATCAAGAG
CTACCAACTCTTTTTCCGAAGGTAACTGGCTTCAGCAGAGCGCAGATACCAAATACTGTTCTTCTAGT
GTAGCCGTAGTTAGGCCACCACTTCAAGAACTCTGTAGCACCGCCTACATACCTCGCTCTGCTAATCC
TGTTACCAGTGGCTGCTGCCAGTGGCGATAAGTCGTGTCTTACCGGGTTGGACTCAAGACGATAGTTA

CCGGATAAGGCGCAGCGGTCGGGCTGAACGGGGGGTTCGTGCACACAGCCCAGCTTGGAGCGAACGAC
CTACACCGAACTGAGATACCTACAGCGTGAGCTATGAGAAAGCGCCACGCTTCCCGAAGGGAGAAAGG
CGGACAGGTATCCGGTAAGCGGCAGGGTCGGAACAGGAGAGCGCACGAGGGAGCTTCCAGGGGGAAAC
GCCTGGTATCTTTATAGTCCTGTCGGGTTTCGCCACCTCTGACTTGAGCGTCGATTTTTGTGATGCTC
GTCAGGGGGGCGGAGCCTATGGAAAAACGCCAGCAACGCGGCCTTTTTACGGTTCCTGGCCTTTTGCT
GGCCTTTTGCTCACATGTTCTTTCCTGCGTTATCCCCTGATTCTGTGGATAACCGTATTACCGCCTTT
GAGTGAGCTGATACCGCTCGCCGCAGCCGAACGACCGAGCGCAGCGAGTCAGTGAGCGAGGAAGCGGA
AGAGCGCCCAATACGCAAACCGCCTCTCCCCGCGCGTTGGCCGATTCATTAATGCAGCTGGCACGACA
GGTTTCCCGACTGGAAAGCGGGCAGTGAGCGCAACGCAATTAATGTGAGTTAGCTCACTCATTAGGCA
CCCCAGGCTTTACACTTTATGCTTCCGGCTCGTATGTTGTGTGGAATTGTGAGCGGATAACAATTTCA
CACAGGAAACAGCTATGACCATGATTACGCCAAGCTCGAAATTAACCCTCACTAAAGGGAACAAAAGC
TGGTACCGGGCCGGCCGTCGGGCCGTCGAGCTTGATGGCATCGTGGTGTCACGCTCGTCGTTTGGTAT
GGCTTCATTCAGCTCCGGTTCCCAACGATCAAGGCGAGTTACATGATCCCCCATGTTGTGCAAAAAAG
CGGTTAGCTCCTTCGGTCCTCCGATCGTTGTCAGAAGTAAGTTGGCCGCAGTGTTATCACTCATGGTT
ATGGCAGCACTGCATAATTCTCTTACTGTCATGCCATCCGTAAGATGCTTTTCTGTGACTGGTGAGTA
CTCAACCAAGTCATTCTGAGAATAGTGTATGCGGCGACCGAGTTGCTCTTGCCCGGCGTCAACACGGG
ATAATACCGCGCCACATAGCAGAACTTTAAAAGTGCTCATCATTGGAAAACGTTCTTCGGGGCGAAAA
CTCTCAAGGATCTTACCGCTGTTGAGATCCAGTTCGATGTAACCCACTCGTGCACCCAACTGATCTTC
AGCATCTTTTACTTTCACCAGCGTTTCTGGGTGAGCAAAAACAGGAAGGCAAAATGCCGCAAAAAAGG
GAATAAGGGCGACACGGAAATGTTGAATACTCATACTCTTCCTTTTTCAATATTATTGAAGCATTTAT
CAGGGTTATTGTCTCATGAGCGGATACATATTTGAATGTATTTAGAAAAATAAACAAATAGGGGTTCC
GCGCACATTTCCCCGAAAAGTGCCACCTGACGTCTAAGAAACCATTATTATCATGACATTAACCTATA
AAAATAGGCGTATCACGAGGCCCTTTCGTCTTCAAGAATTGGGGATCTACGTATGGTCATTTCTTCTT
CAGATTCCCTCATGGAGAAAGTGCGGCAGATGTATATGACAGAGTCGCCAGTTTCCAAGAGACTTTAT
TCAGGCACTTCCATGATAGGCAAGAGAGAAGACCCAGAGATGTTGTTGTCCTAGTTACACATGGTATT
TATTCCAGAGTATTCCTGATGAAATGGTTTAGATGGACATACGAAGAGTTTGAATCGTTTACCAATGT
TCCTAACGGGAGCGTAATGGTGATGGAACTGGACGAATCCATCAATAGATACGTCCTGAGGACCGTGC
TACCCAAATGGACTGATTGTGAGGGAGACCTAACTACATAGTGTTTAAAGATTACGGATATTTAACTT
ACTTAGAATAATGCCATTTTTTTGAGTTATAATAATCCTACGTTAGTGTGAGCGGGATTTAAACTGTG
AGGACCTTAATACATTCAGACACTTCTGCGGTATCACCCTACTTATTCCCTTCGAGATTATATCTAGG
AACCCATCAGGTTGGTGGAAGATTACCCGTTCTAAGACTTTTCAGCTTCCTCTATTGATGTTACACCT
GGACACCCCTTTTCTGGCATCCAGTTTTTAATCTTCAGTGGCATGTGAGATTCTCCGAAATTAATTAA
AGCAATCACACAATTCTCTCGGATACCACCTCGGTTGAAACTGACAGGTGGTTTGTTACGCATGCTAA
TGCAAAGGAGCCTATATACCTTTGGCTCGGCTGCTGTAACAGGGAATATAAAGGGCAGCATAATTTAG
GAGTTTAGTGAACTTGCAACATTTACTATTTTCCCTTCTTACGTAAATATTTTTCTTTTTAATTCTAA
ATCAATCTTTTTCAATTTTTTGTTTGTATTCTTTTCTTGCTTAAATCTATAACTACAAAAAACACATA
CAG

Figure S6: Vector map and nucleotide sequence of *GCN4* expression analysis plasmid, B1805-HIS

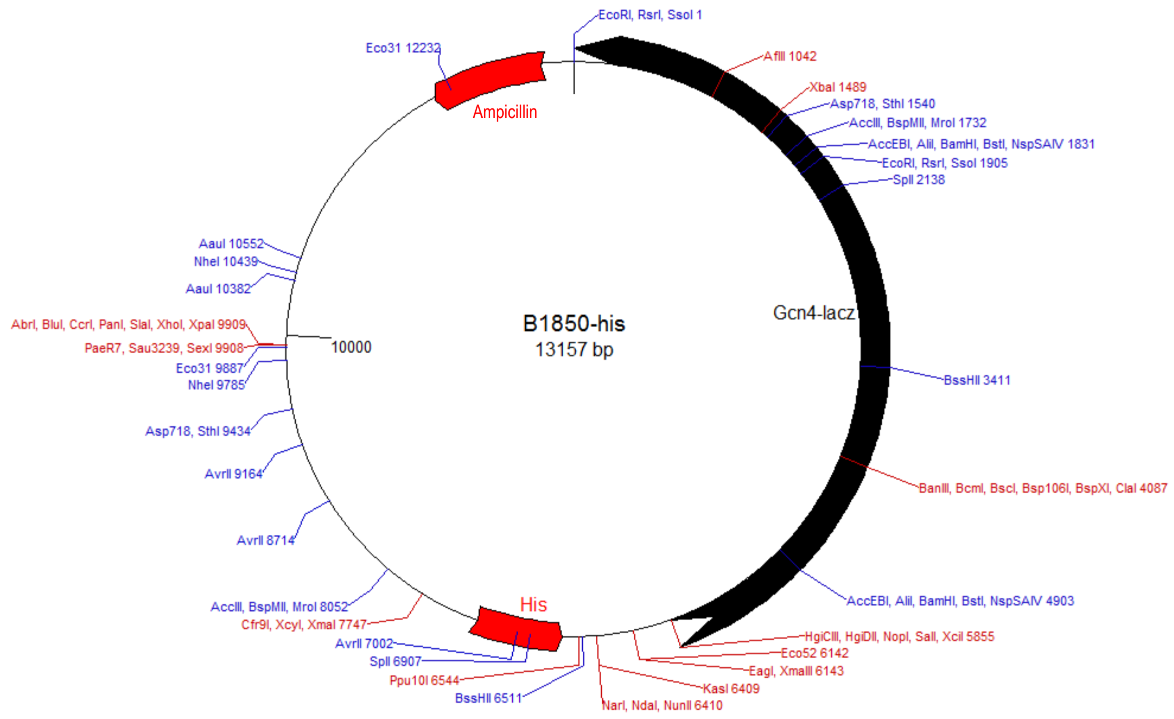

GAATTCTCTAGCTTAAAATGAATAGTTGATTCTTTTGCCAAAGACGAAAATAGTTGCCAGTAAGCATC
TCTTATGAAATAAGTGTTGTTGTGAATAGCTCAATAGAGATGTATTATTAATAAGCCTCGTATTTTCA
GGGATCATGCGTAGCATAATGGCAGTTGGTAAGGGGAATAAGAATGATGATTCCATTATTGCGAATAA
AGGTATTTCTGAAAGTACATCTAATGGGCTATTTGAAAATTTGTTTAGGATTATGGTAATAAAAGAGG
CATTAACGCCAGAACCTTTATTTTATCACTTTTTCGATATTTGTTAATAATCTAAATCTAACTTATAT
CTAAACCTTAGCGTTTGCATTCTTGATTTCTATATTTTTATTCACAATATGGAAAGGAAAAACAATCT
CTTAGGTTGTCCAAGAAAACTTCTCTCCCTGTCATACTCAAAGTGTTGGAACAAAAATCAACTATCGT
CTATCAACTAGTAGCTATACTACTAATATATTATCATATACGGTGTTAGATGATGACATAAGTTATGA
GAAGCTGTCATCGAAGTTAGAAGAAGCTGAAATGCAAGGATTGATAATGTAATAGAATTTAATGAAAC
ATATAAAACGGAATGAGGAATAATCGTAATATTAGTATGTAAAAATATGGATTCCATTTTGAGGATTC
CTATATCCATGAGGAGAACTTCTAGTATATGCTGTATACATAATACTATAGCCTTGATCAACAATGGA
ACCCCAACAATTATCTCACAATTCACCCATTTCTCAACTAGTAACATGAGTACTCCTAAATAGGGCGA
TATTTTAAAGTTTCATTCCAGCATTAGCTATAACACGTTAATATGGTGGAGTCAGCTGAGAAGTTTTT
TCAATAAATAATGCTCGCGTGGCGTAATGGCAACGCGTCTGACTTCTAATCAGAAGATTATGGGTTCG
ACCCCCATCGTGAGTGTTTTTTTTATCTATTCCTAACCAGTAAATACCAGAACATACGGCAGATTATA
AATGCGTGGTGTAAAATTCTACTTAAGAAAATTGGCATAAAAAAGATTAAATTCTTATCTAAGTGAAT
GTATCTATTTCGTTATACACGAGAATGAAATAAAAAATATAAAATAAAAGGTAAATGAAATCAGCGTT
CGCCAACTAATTTCTTTAATCTGGCAACCTCATTTTCCAAGTGATAATTTTTCGAAAGCAATTCTTCA
ACCTTGTCTTCAAGTTGTTTCATTCTTTGCAACTTTCTCGCACGAGAACGCCTGGCGGCTTCAGTGTT
TCTAGCACGTTTTAGAGCAGCAGGATCACTGGATTCGGGCACAATTGGAGAAAGTGGAATCGAACGCT
GTTTGCGGTTGTAAGCAACAACACCTAGATGATCCAGTCTCGATTCGTCATCCTTTCCAACATGATGT
GACTTCTTAACGACTGAATTTGGTTTCTTAACCTTTCTTGTTTGAGTCAGTTTAGCATCTTCTAGAAC
AGGAGTGGGTAAGAATGAAGTTGTCGAGACTTCCAGATTGGATGGTACCAGAGAAACTTCTTCAGTGG
ATTCAATTGCCTTATCAGCCAATGAAACATCGTCAGTGGTAACTGGAATGTCATTGTCAAACAAGGAT

GTCCATTCTTTAGAGTTGTCTTCTAGGTTTTCATACTCAAACATTGGAGTTGAATCAGTGCTTGACGA
AAAGAAAGATTCCACTACAGCGTCATCTAGCTCCGGAATTGGCAAAACGGTCTTGGCATCAGGTGCAG
TTGCCGTTTGTGGAAGAGCAAAATCAAAATCAAGGTTCGAAGGGGTATCCTGTTTGATAATTGGATCC
CCCCTGCCCGGTTATTATTATTTTTGACACCAGACCAACTGGTAATGGTAGCGACCGGCGCTCAGCTG
GAATTCCGCCGATACTGACGGGCTCCAGGAGTCGTCGCCACCAATCCCCATATGGAAACCGTCGATAT
TCAGCCATGTGCCTTCTTCCGCGTGCAGCAGATGGCGATGGCTGGTTTCCATCAGTTGCTGTTGACTG
TAGCGGCTGATGTTGAACTGGAAGTCGCCGCGCCACTGGTGTGGGCCATAATTCAATTCGCGCGTCCC
GCAGCGCAGACCGTTTTCGCTCGGGAAGACGTACGGGGTATACATGTCTGACAATGGCAGATCCCAGC
GGTCAAAACAGGCGGCAGTAAGGCGGTCGGGATAGTTTTCTTGCGGCCCTAATCCGAGCCAGTTTACC
CGCTCTGCTACCTGCGCCAGCTGGCAGTTCAGGCCAATCCGCGCCGGATGCGGTGTATCGCTCGCCAC
TTCAACATCAACGGTAATCGCCATTTGACCACTACCATCAATCCGGTAGGTTTTCCGGCTGATAAATA
AGGTTTTCCCCTGATGCTGCCACGCGTGAGCGGTCGTAATCAGCACCGCATCAGCAAGTGTATCTGCC
GTGCACTGCAACAACGCTGCTTCGGCCTGGTAATGGCCCGCCGCCTTCCAGCGTTCGACCCAGGCGTT
AGGGTCAATGCGGGTCGCTTCACTTACGCCAATGTCGTTATCCAGCGGTGCACGGGTGAACTGATCGC
GCAGCGGCGTCAGCAGTTGTTTTTTATCGCCAATCCACATCTGTGAAAGAAAGCCTGACTGGCGGTTA
AATTGCCAACGCTTATTACCCAGCTCGATGCAAAAATCCATTTCGCTGGTGGTCAGATGCGGGATGGC
GTGGGACGCGGCGGGGAGCGTCACACTGAGGTTTTCCGCCAGACGCCACTGCTGCCAGGCGCTGATGT
GCCCGGCTTCTGACCATGCGGTCGCGTTCGGTTGCACTACGCGTACTGTGAGCCAGAGTTGCCCGGCG
CTCTCCGGCTGCGGTAGTTCAGGCAGTTCAATCAACTGTTTACCTTGTGGAGCGACATCCAGAGGCAC
TTCACCGCTTGCCAGCGGCTTACCATCCAGCGCCACCATCCAGTGCAGGAGCTCGTTATCGCTATGAC
GGAACAGGTATTCGCTGGTCACTTCGATGGTTTGCCCGGATAAACGGAACTGGAAAAACTGCTGCTGG
TGTTTTGCTTCCGTCAGCGCTGGATGCGGCGTGCGGTCGGCAAAGACCAGACCGTTCATACAGAACTG
GCGATCGTTCGGCGTATCGCCAAAATCACCGCCGTAAGCCGACCACGGGTTGCCGTTTTCATCATATT
TAATCAGCGACTGATCCACCCAGTCCCAGACGAAGCCGCCCTGTAAACGGGGATACTGACGAAACGCC
TGCCAGTATTTAGCGAAACCGCCAAGACTGTTACCCATCGCGTGGGCGTATTCGCAAAGGATCAGCGG
GCGCGTCTCTCCAGGTAGCGAAAGCCATTTTTTGATGGACCATTTCGGCACAGCCGGGAAGGGCTGGT
CTTCATCCACGCGCGCGTACATCGGGCAAATAATATCGGTGGCCGTGGTGTCGGCTCCGCCGCCTTCA
TACTGCACCGGGCGGGAAGGATCGACAGATTTGATCCAGCGATACAGCGCGTCGTGATTAGCGCCGTG
GCCTGATTCATTCCCCAGCGACCAGATGATCACACTCGGGTGATTACGATCGCGCTGCACCATTCGCG
TTACGCGTTCGCTCATCGCCGGTAGCCAGCGCGGATCATCGGTCAGACGATTCATTGGCACCATGCCG
TGGGTTTCAATATTGGCTTCATCCACCACATACAGGCCGTAGCGGTCGCACAGCGTGTACCACAGCGG
ATGGTTCGGATAATGCGAACAGCGCACGGCGTTAAAGTTGTTCTGCTTCATCAGCAGGATATCCTGCA
CCATCGTCTGCTCATCCATGACCTGACCATGCAGAGGATGATGCTCGTGACGGTTAACGCCTCGAATC
AGCAACGGCTTGCCGTTCAGCAGCAGCAGACCATTTTCAATCCGCACCTCGCGGAAACCGACATCGCA
GGCTTCTGCTTCAATCAGCGTGCCGTCGGCGGTGTGCAGTTCAACCACCGCACGATAGAGATTCGGGA
TTTCGGCGCTCCACAGTTTCGGGTTTTCGACGTTCAGACGTAGTGTGACGCGATCGGCATAACCACCA
CGCTCATCGATAATTTCACCGCCGAAAGGCGCGGTGCCGCTGGCGACCTGCGTTTCACCCTGCCATAA
AGAAACTGTTACCCGTAGGTAGTCACGCAACTCGCCGCACATCTGAACTTCAGCCTCCAGTACAGCGC
GGCTGAAATCATCATTAAAGCGAGTGGCAACATGGAAATCGCTGATTTGTGTAGTCGGTTTATGCAGC
AACGAGACGTCACGGAAAATGCCGCTCATCCGCCACATATCCTGATCTTCCAGATAACTGCCGTCACT
CCAACGCAGCACCATCACCGCGAGGCGGTTTTCTCCGGCGCGTAAAAATGCGCTCAGGTCAAATTCAG
ACGGCAAACGACTGTCCTGGCCGTAACCGACCCAGCGCCCGTTGCACCACAGATGAAACGCCGAGTTA
ACGCCATCAAAAATAATTCGCGTCTGGCCTTCCTGTAGCCAGCTTTCATCAACATTAAATGTGAGCGA
GTAACAACCCGTCGGATTCTCCGTGGGAACAAACGGCGGATTGACCGTAATGGGATAGGTTACGTTGG
TGTAGATGGGCGCATCGTAACCGTGCATCTGCCAGTTTGAGGGGACGACGACAGTATCGGCCTCAGGA
AGATCGCACTCCAGCCAGCTTTCCGGCACCGCTTCTGGTGCCGGAAACCAGGCAAAGCGCCATTCGCC
ATTCAGGCTGCGCAACTGTTGGGAAGGGCGATCGGTGCGGGCCTCTTCGCTATTACGCCAGCTGGCGA
AAGGGGGATGTGCTGCAAGGCGATTAAGTTGGGTAACGCCAGGGTTTTCCCAGTCACGACGTTGTAAA

ACGACGGGATCCTCTTCAGTCTTGATGAATTTATCAAAAATCAATTGGCCAACCATTGGTTTGGCAGT
AGAAGTGGAAGCAGATACATTTTCGTTGGTTGATTTAGAACCATCCAATGGTGAGAAACCCATTGGAT
TTAAAGCAAATAAACTTGGCTGATATTCGGACATTTTATTTGTATTTAATTTATTTTCTTGAGCAGAC
AAATTGGTAAACAAAACTTTAGTAATAATAATGATTTAATTAATGATAGTATAGGGAAATTTTTATTG
GCGAGTAAACCTGGATAATTTGACAGAAAGGTAACCGTTACGGAAACATCTTGAATAAAATTCTACGG
GTACATGATAGCAATTGGTAACAAAACAAATAACTCTTCAAAAAACTGACAGTTTTCAAAAAAAGTAA
AGGACTTTAATTAATAAGGGAAAATAAATTTTCTCTTTCAATAAATTTAACACATAATTCTCTTAATA
ATTTTCTAATAATAATCTACTTTAAAAACAAAATATAATCGGTTTAGCAAGCCATTTTTCAATGATCT
TTAATTTTTTAATACGATACTGATAATAACTTAATAAACTGAACTAAAATAAAATATTTTGTTTTGAT
TGCGAAGTAGATGAGTGAGCTGTGTGGCTGGTGAGTTGTATAATTCGCTAGTGAAACTGATGGGCAAA
AAAAATTTGAATTTAGGGGGGAGAGTAACCTGTGTTGTGAGTTTTTGTTTTGTTTTGTTTTGTATATC
TATTATATAAGAAGATAAGTACTGTCAAGAAGTAGAAGATTTTAAAAGGTAAGACAGCGAGCCGAAAC
TTCTTTAAAGAAGACTAAAATTTCGGAATTGGAAAAAAAGGTTGGAGTATTTATCTTCCGTATTTAAA
GTCGGAAATTTTTCATCTTTTTTTTTCAAGATGTATGCTCATGCACTTTATTCCCCGAAGATCCACAG
GACGGGGTCGACCGATGCCCTTGAGAGCCTTCAACCCAGTCAGCTCCTTCCGGTGGGCGCGGGGCATG
ACTATCGTCGCCGCACTTATGACTGTCTTCTTTATCATGCAACTCGTAGGACAGGTGCCGGCAGCGCT
CTGGGTCATTTTCGGCGAGGACCGCTTTCGCTGGAGCGCGACGATGATCGGCCTGTCGCTTGCGGTAT
TCGGAATCTTGCACGCCCTCGCTCAAGCCTTCGTCACTGGTCCCGCCACCAAACGTTTCGGCGAGAAG
CAGGCCATTATCGCCGGCATGGCGGCCGACGCGCTGGGCTACGTCTTGCTGGCGTTCGCGACGCGAGG
CTGGATGGCCTTCCCCATTATGATTCTTCTCGCTTCCGGCGGCATCGGGATGCCCGCGTTGCAGGCCA
TGCTGTCCAGGCAGGTAGATGACGACCATCAGGGACAGCTTCAAGGATCGCTCGCGGCTCTTACCAGC
CTAACTTCGATCACTGGACCGCTGATCGTCACGGCGATTTATGCCGCCTCGGCGAGCACATGGAACGG
GTTGGCATGGATTGTAGGCGCCGTATTTTCTCCTTACGCATCTGTGCGGTATTTCACACCGCATAGAT
CCGTCGAGTTCAAGAGAAAAAAAAAGAAAAAGCAAAAAGAAAAAAGGAAAGCGCGCCTCGTTCAGAAT
GACACGTATAGAATGATGCATTACCTTGTCATCTTCAGTATCATACTGTTCGTATACATACTTACTGA
CATTCATAGGTATACATATATACACATGTATATATATCGTATGCTGCAGCTTTAAATAATCGGTGTCA
CTACATAAGAACACCTTTGGTGGAGGGAACATCGTTGGTTCCATTGGGCGAGGTGGCTTCTCTTATGG
CAACCGCAAGAGCCTTGAACGCACTCTCACTACGGTGATGATCATTCTTGCCTCGCAGACAATCAACG
TGGAGGGTAATTCTGCTTGCCTCTGCAAAACTTTCAAGAAAATGCGGGATCATCTCGCAAGAGAGATC
TCCTACTTTCTCCCTCTGCAAACCAAGTTCGACAACTGCGTACGGCCTGTTCGAAAGATCTACCACCG
CTCTGGAAAGTGCCTCATCCAAAGGCGCAAATCCTGATCCAAACCTTTTTACTCCACGCACGGCCCCT
AGGGCCTCTTTAAATGCTTGACCGAGAGCAATCCCGCAGTCTTCAGTGGTGTGATGGTCGTCTATGTG
TAAGTCACCAATGCACTCAACGATTAGCGACCAGCCGGAATGCTTGGCCAGAGCATGTATCATATGGT
CCAGAAACCCTATACCTGTGTGGACGTTAATCACTTGCGATTGTGTGGCCTGTTCTGCTACTGCTTCT
GCCTCTTTTTCTGGGAAGATCGAGTGCTCTATCGCTAGGGGACCACCCTTTAAAGAGATCGCAATCTG
AATCTTGGTTTCATTTGTAATACGCTTTACTAGGGCTTTCTGCTCTGTCATCTTTGCCTTCGTTTATC
TTGCCTGCTCATTTTTTAGTATATTCTTCGAAGAAATCACATTACTTTATATAATGTATAATTCATTA
TGTGATAATGCCAATCGCTAAGAAAAAAAAAGAGTCATCCGCTAGGGGAAAAAAAAAAAATGAAAATCA
TTACCGAGGCATAAAAAAATATAGAGTGTACTAGAGGAGGCCAAGAGTAATAGAAAAAGAAAATTGCG
GGAAAGGACTGTGTTATGACTTCCCTGACTAATGCCGTGTTCAAACGATACCTGGCAGTGACTCCTAG
CGCTCACCAAGCTCTTAAAACGGGAATTTATGGTGCACTCTCAGTACAATCTGCTCTGATGCCGCATA
GTTAAGCCAGCCCCGACACCCGCCAACACCCGCTGACGCGCCCTGACGGGCTTGTCTGCTCCCCCGGG
AATCTCGGTCGTAATGATTTTTATAATGACGAAAAAAAAAAAAATTGGAAAGAAAACCCCCCCCCCGCA
GCGTTGGGTCCTGGCCACGGGTGCGCATGATCGTGCTCCTGTCGTTGAGGACCCGGCTAGGCTGGCGG
GGTTGCCTTACTGGTTAGCAGAATGAATCACCGATACGCGAGCGAACGTGAAGCGACTGCTGCTGCAA
AACGTCTGCGACCTGAGCAACAACATGAATGGTCTTCGGTTTCCGTGTTTCGTAAAGTCTGGAAACGC
GGAAGTCAGCGCCCTGCACCATTATGTTCCGGATCTGCATCGCAGGATGCTGCTGGCTACCCTGTGGA
ACACCTACATCTGTATTAACGAAGCGCTGGCATTGACCCTGAGTGATTTTTCTCTGGTCCCGCCGCAT

CCATACCGCCAGTTGTTTACCCTCACAACGTTCCAGTAACCGGGCATGTTCATCATCAGTAACCCGTA
TCGTGAGCATCCTCTCTCGTTTCATCGGTATCATTACCCCCATGAACAGAAATTCCCCCTTACACGGA
GGCATCAAGTGACCAAACAGGAAAAAACCGCCCTTAACATGGCCCGCTTTATCAGAAGCCAGACATTA
ACGCTTCTGGAGAAACTCAACGAGCTGGACGCGGATGAACAGGCAGACATCTGTGAATCGCTTCACGA
CCACGCTGATGAGCTTTACCGCAGGTGGGCCATTCTCATGAAGAATATCTTGAATTTATTGTCATATT
ACTAGTTGGTGTGGAAGTCCATATATCGGTGATCAATATAGTGGTTGACATGCTGGCTAGTCAACATT
GAGCCTTTTGATCATGCAAATATATTACGGTATTTTACAATCAAATATCAAACTTAACTATTGACTTT
ATAACTTATTTAGGTGGTAACATTCTTATAAAAAAGAAAAAAATTACTGCAAAACAGTACTAGCTTTT
AACTTGTATCCTAGGTTATCTATGCTGTCTCACCATAGAGAATATTACCTATTTCAGAATGTATGTCC
ATGATTCGCCGGGTAAATACATATAATACACAAATCTGGCTTAATAAAGTCTATAATATATCTCATAA
AGAAGTGCTAAATTGGCTAGTGCTATATATTTTTAAGAAAATTTCTTTTGACTAAGTCCATATCGACT
TTGTAAAAGTTCACTTTAGCATACATATATTACACGAGCCAGAAATTGTAACTTTTGCCTAAAATCAC
AAATTGCAAAATTTAATTGCTTGCAAAAGGTCACATGCTTATAATCAACTTTTTTAAAAATTTAAAAT
ACTTTTTTATTTTTTATTTTTAAACATAAATGAAATAATTTATTTATTGTTTATGATTACCGAAACAT
AAAACCTGCTCAAGAAAAAGAAACTGTTTTGTCCTTGGAAAAAAAGCACTACCTAGGAGCGGCCAAAA
TGCCGAGGCTTTCATAGCTTAAACTCTTTACAGAAAATAGGCATTATAGATCAGTTCGAGTTTTCTTA
TTCTTCCTTCCGGTTTTATCGTCACAGTTTTACAGTAAATAAGTATCACCTCTTAGAGTTAACTATGA
GATAAGCAAGTATCATCTCATTTCATTTACCTGAAGTCGAGTAAACAGAAAATCCAATTGTTGATGAA
CCTCAATGACTTAGAACTATCTATCGGCAGATCATATAAAGAGGATTTAGGTACCTAGAGGACTGTAC
CTGGAGTATATATATATATATATATATATTATCTCAACTATAGTCCATAGAGGTTTCTTTCTTGAGGC
CTTAAACTGCTAAAGAATGATATTGGTGGAATGCAAGCACCAATCTCTCTTCTTTCGTAACTGTTCAT
ATACTTCAAACCAAGAATGTAACGGGCATTGACCCATCCAAAACCTTCAGTAGCTGCCCCTTTAAAGT
CAGCACCTTGATTACCGTATTCTGCTTCAACACGATGAGGATCTGTTCCTCTTGTGACATCATATTTT
TCAACCACAATACCATTATAATCGACAAAAGCCTTTGTCATCATGAAAAGCCATCTATAAGCTAGCCT
ATTCGTTACAGTTAAATAACCATAAGAACGGAGGCCTTCCCAAGCAAGAATTTGATGGGGTGCCCAAC
CAAATGGATAGTCCCATTGTCTAATTGGTCTCGAAATAGAAATTGGGCCTCGAGAACGCTCCGTACAT
GCAGCTAAACCTCCAAGCATCTCTAACTTGGGTAGTGCTTTCTCCACCATTTTCTGTGCTTGCTCCTT
CGTGGCAAGTCCAGCCCATAATGCCCAGAATGTAGTTGCGGATTCGTATGACGTTCTGTGCTTGATTT
TTGTGTTGTAGTCAAAGAAAAACCCCGACTCGTCATCCCACATATATTTGGTAATTGATGAGGCAACG
CTAATTATCAACATATAGATTGTTATCTATCTGCATGAACACGAAATCTTTACTTGACGACTTGAGGC
TGATGGTGTTTATGCAAAGAAACCACTGTGTTTAATATGTGTCACTGTTTGATATTACTGTCAGCGTA
GAAGATAATAGTAAAAGCGGTTAATAAGTGTATTTGAGATAAGTGTGATAAAGTTTTTACAGCGAAAA
GACGATAAATACAAGAAAATGATTACGAGGATACGGAGAGAGGTATGTACATGTGTATTTATATACTA
AGCTGCCGGCGGTTGTTTGCAAGACCGAGAAAAGGCTAGCAAGAATCGGGTCATTGTAGCGTATGCGC
CTGTGAACATTCTCTTCAACAAGTTTGATTCCATTGCGGTGAAATGGTAAAAGTCAACCCCCTGCGAT
GTATATTTTCCTGTACAATCAATCAAAAAGCCAAATGATTTAGCATTATCTTTACATCTTGTTATTTT
ACAGATTTTATGTTTAGATCTTTTATGCTTGCTTTTCAAAAGGCCTGCAGGCAAGTGCACAAACAATA
CTTAAATAAATACTACTCAGTAATAACCTATTTCTTAGCATTTTTGACGAAATTTGCTATTTTGTTAG
AGTCTTTTACACCATTTGTCTCCACACCTCCGCTTACATCAACACCAATAACGCCATTTAATCTAAGC
GCATCACCAACATTTTCTGGCGTCAGTCCACCAGCTAACATAAAATGTAAGCTCTGCCTCGCGCGTTT
CGGTGATGACGGTGAAAACCTCTGACACATGCAGCTCCCGGAGACGGTCACAGCTTGTCTGTAAGCGG
ATGCCGGGAGCAGACAAGCCCGTCAGGGCGCGTCAGCGGGTGTTGGCGGGTGTCGGGGCGCAGCCATG
ACCCAGTCACGTAGCGATAGCGGAGTGTATACTGGCTTAACTATGCGGCATCAGAGCAGATTGTACTG
AGAGTGCACCATATGCGGTGTGAAATACCGCACAGATGCGTAAGGAGAAAATACCGCATCAGGCGCTC
TTCCGCTTCCTCGCTCACTGACTCGCTGCGCTCGGTCGTTCGGCTGCGGCGAGCGGTATCAGCTCACT
CAAAGGCGGTAATACGGTTATCCACAGAATCAGGGGATAACGCAGGAAAGAACATGTGAGCAAAAGGC
CAGCAAAAGGCCAGGAACCGTAAAAAGGCCGCGTTGCTGGCGTTTTTCCATAGGCTCCGCCCCCCTGA
CGAGCATCACAAAAATCGACGCTCAAGTCAGAGGTGGCGAAACCCGACAGGACTATAAAGATACCAGG

CGTTTCCCCCTGGAAGCTCCCTCGTGCGCTCTCCTGTTCCGACCCTGCCGCTTACCGGATACCTGTCC
GCCTTTCTCCCTTCGGGAAGCGTGGCGCTTTCTCATAGCTCACGCTGTAGGTATCTCAGTTCGGTGTA
GGTCGTTCGCTCCAAGCTGGGCTGTGTGCACGAACCCCCCGTTCAGCCCGACCGCTGCGCCTTATCCG
GTAACTATCGTCTTGAGTCCAACCCGGTAAGACACGACTTATCGCCACTGGCAGCAGCCACTGGTAAC
AGGATTAGCAGAGCGAGGTATGTAGGCGGTGCTACAGAGTTCTTGAAGTGGTGGCCTAACTACGGCTA
CACTAGAAGGACAGTATTTGGTATCTGCGCTCTGCTGAAGCCAGTTACCTTCGGAAAAAGAGTTGGTA
GCTCTTGATCCGGCAAACAAACCACCGCTGGTAGCGGTGGTTTTTTTGTTTGCAAGCAGCAGATTACG
CGCAGAAAAAAAGGATCTCAAGAAGATCCTTTGATCTTTTCTACGGGGTCTGACGCTCAGTGGAACGA
AAACTCACGTTAAGGGATTTTGGTCATGAGATTATCAAAAAGGATCTTCACCTAGATCCTTTTAAATT
AAAAATGAAGTTTTAAATCAATCTAAAGTATATATGAGTAAACTTGGTCTGACAGTTACCAATGCTTA
ATCAGTGAGGCACCTATCTCAGCGATCTGTCTATTTCGTTCATCCATAGTTGCCTGACTCCCCGTCGT
GTAGATAACTACGATACGGGAGGGCTTACCATCTGGCCCCAGTGCTGCAATGATACCGCGAGACCCAC
GCTCACCGGCTCCAGATTTATCAGCAATAAACCAGCCAGCCGGAAGGGCCGAGCGCAGAAGTGGTCCT
GCAACTTTATCCGCCTCCATCCAGTCTATTAATTGTTGCCGGGAAGCTAGAGTAAGTAGTTCGCCAGT
TAATAGTTTGCGCAACGTTGTTGCCATTGCTGCAGGCATCGTGGTGTCACGCTCGTCGTTTGGTATGG
CTTCATTCAGCTCCGGTTCCCAACGATCAAGGCGAGTTACATGATCCCCCATGTTGTGCAAAAAAGCG
GTTAGCTCCTTCGGTCCTCCGATCGTTGTCAGAAGTAAGTTGGCCGCAGTGTTATCACTCATGGTTAT
GGCAGCACTGCATAATTCTCTTACTGTCATGCCATCCGTAAGATGCTTTTCTGTGACTGGTGAGTACT
CAACCAAGTCATTCTGAGAATAGTGTATGCGGCGACCGAGTTGCTCTTGCCCGGCGTCAACACGGGAT
AATACCGCGCCACATAGCAGAACTTTAAAAGTGCTCATCATTGGAAAACGTTCTTCGGGGCGAAAACT
CTCAAGGATCTTACCGCTGTTGAGATCCAGTTCGATGTAACCCACTCGTGCACCCAACTGATCTTCAG
CATCTTTTACTTTCACCAGCGTTTCTGGGTGAGCAAAAACAGGAAGGCAAAATGCCGCAAAAAAGGGA
ATAAGGGCGACACGGAAATGTTGAATACTCATACTCTTCCTTTTTCAATATTATTGAAGCATTTATCA
GGGTTATTGTCTCATGAGCGGATACATATTTGAATGTATTTAGAAAAATAAACAAATAGGGGTTCCGC
GCACATTTCCCCGAAAAGTGCCACCTGACGTCTAAGAAACCATTATTATCATGACATTAACCTATAAA
AATAGGCGTATCACGAGGCCCTTTCGTCTTCAA

Figure S7: Vector map and nucleotide sequence of expression plasmid pYX222-alphaSS-GFP, where alpha SS is the *S. cerevisiae* mating factor α secretion signal

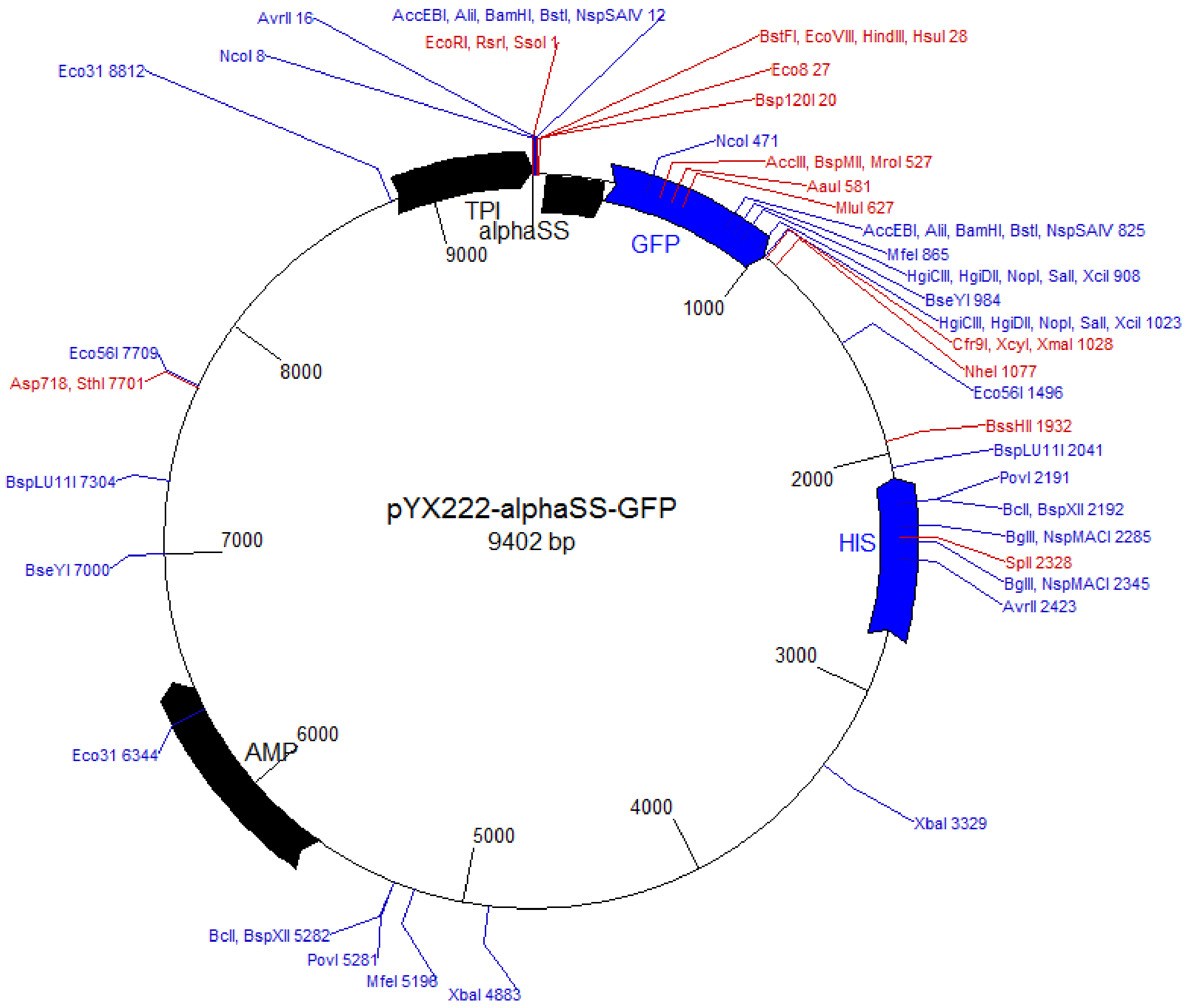

GAATTCACCATGGATCCTAGGGCCCACAAGCTTAACAAAATGAGATTTCCTTCAATTTTTACTGCAGT
TTTATTCGCAGCATCCTCCGCATTAGCTGCTCCAGTCAACACTACAACAGAAGATGAAACGGCACAAA
TTCCGGCTGAAGCTGTCATCGGTTACTCAGATTTAGAAGGGGATTTCGATGTTGCTGTTTTGCCATTT
TCCAACAGCACAAATAACGGGTTATTGTTTATAAATACTACTATTGCCAGCATTGCTGCTAAAGAAGA
AGGGGTATCTCTCGAGAAAAGAGAGGCTGAAGTATGAGTAAAGGAGAAGAACTTTTCACTGGAGTTGT
CCCAATTCTTGTTGAATTAGATGGTGATGTTAATGGGCACAAATTTTCTGTCAGTGGAGAGGGTGAAG
GTGATGCAACATACGGAAAACTTACCCTTAAATTTATTTGCACTACTGGAAAACTACCTGTTCCATGG
CCAACACTTGTCACTACTTTCTCTTATGGTGTTCAATGCTTTTCCCGTTATCCGGATCATATGAAACG
GCATGACTTTTTCAAGAGTGCCATGCCCGAAGGTTATGTACAGGAACGCACTATATCTTTCAAAGATG
ACGGGAACTACAAGACGCGTGCTGAAGTCAAGTTTGAAGGTGATACCCTTGTTAATCGTATCGAGTTA
AAAGGTATTGATTTTAAAGAAGATGGAAACATTCTCGGACACAAACTCGAGTACAACTATAACTCACA
CAATGTATACATCACGGCAGACAAACAAAAGAATGGAATCAAAGCTAACTTCAAAATTCGCCACAACA
TTGAAGATGGATCCGTTCAACTAGCAGACCATTATCAACAAAATACTCCAATTGGCGATGGCCCTGTC
CTTTTACCAGACAACCATTACCTGTCGACACAATCTGCCCTTTCGAAAGATCCCAACGAAAAGCGTGA
CCACATGGTCCTTCTTGAGTTTGTAACTGCTGCTGGGATTACACATGGCATGGATGAGCTCTACAAAT
AAGTCGACCCGGGTATCCGTATGATGTGCCTGACTACGCATGATATCTCGAGCTCAGCTAGCTAACTG
AATAAGGAACAATGAACGTTTTTCCTTTCTCTTGTTCCTAGTATTAATGACTGACCGATACATCCCTT

TTTTTTTTTGTCTTTGTCTAGCTCCAATTCGCCCTATAGTGAGTCGTATTACAATTCACTGGCCGTCG
TTTTACAACGTCGTGACTGGGAAAACCCTGGCGTTACCCAACTTAATCGCCTTGCAGCACATCCCCCT
TTCGCCAGCTGGCGTAATAGCGAAGAGGCCCGCACCGATCGCCCTTCCCAACAGTTGCGCAGCCTGAA
TGGCGAATGGACGCGCCCTGTAGCGGCGCATTAAGCGCGGCGGGTGTGGTGGTTACGCGCAGCGTGAC
CGCTACACTTGCCAGCGCCCTAGCGCCCGCTCCTTTCGCTTTCTTCCCTTCCTTTCTCGCCACGTTCG
CCGGCTTTCCCCGTCAAGCTCTAAATCGGGGGCTCCCTTTAGGGTTCCGATTTAGTGCTTTACGGCAC
CTCGACCCCAAAAAACTTGATTAGGGTGATGGTTCACGTAGTGGGCCATCGCCCTGATAGACGGTTTT
TCGCCCTTTGACGTTGGAGTCCACGTTCTTTAATAGTGGACTCTTGTTCCAAACTGGAACAACACTCA
ACCCTATCTCGGTCTATTCTTTTGATTTATAAGGGATTTTGCCGATTTCGGCCTATTGGTTAAAAAAT
GAGCTGATTTAACAAAAATTTAACGCGAATTTTAACAAAATATTAACGCTTACAATTTCCTGATGCGG
TATTTTCTCCTTACGCATCTGTGCGGTATTTCACACCGCATAGATCCGTCGAGTTCAAGAGAAAAAAA
AAGAAAAAGCAAAAAGAAAAAAGGAAAGCGCGCCTCGTTCAGAATGACACGTATAGAATGATGCATTA
CCTTGTCATCTTCAGTATCATACTGTTCGTATACATACTTACTGACATTCATAGGTATACATATATAC
ACATGTATATATATCGTATGCTGCAGCTTTAAATAATCGGTGTCACTACATAAGAACACCTTTGGTGG
AGGGAACATCGTTGGTTCCATTGGGCGAGGTGGCTTCTCTTATGGCAACCGCAAGAGCCTTGAACGCA
CTCTCACTACGGTGATGATCATTCTTGCCTCGCAGACAATCAACGTGGAGGGTAATTCTGCTTGCCTC
TGCAAAACTTTCAAGAAAATGCGGGATCATCTCGCAAGAGAGATCTCCTACTTTCTCCCTCTGCAAAC
CAAGTTCGACAACTGCGTACGGCCTGTTCGAAAGATCTACCACCGCTCTGGAAAGTGCCTCATCCAAA
GGCGCAAATCCTGATCCAAACCTTTTTACTCCACGCACGGCCCCTAGGGCCTCTTTAAATGCTTGACC
GAGAGCAATCCCGCAGTCTTCAGTGGTGTGATGGTCGTCTATGTGTAAGTCACCAATGCACTCAACGA
TTAGCGACCAGCCGGAATGCTTGGCCAGAGCATGTATCATATGGTCCAGAAACCCTATACCTGTGTGG
ACGTTAATCACTTGCGATTGTGTGGCCTGTTCTGCTACTGCTTCTGCCTCTTTTTCTGGGAAGATCGA
GTGCTCTATCGCTAGGGGACCACCCTTTAAAGAGATCGCAATCTGAATCTTGGTTTCATTTGTAATAC
GCTTTACTAGGGCTTTCTGCTCTGTCATCTTTGCCTTCGTTTATCTTGCCTGCTCATTTTTTAGTATA
TTCTTCGAAGAAATCACATTACTTTATATAATGTATAATTCATTATGTGATAATGCCAATCGCTAAGA
AAAAAAAAGAGTCATCCGCTAGGGGAAAAAAAAAAAATGAAAATCATTACCGAGGCATAAAAAAATATA
GAGTGTACTAGAGGAGGCCAAGAGTAATAGAAAAAGAAAATTGCGGGAAAGGACTGTGTTATGACTTC
CCTGACTAATGCCGTGTTCAAACGATACCTGGCAGTGACTCCTAGCGCTCACCAAGCTCTTAAAACGG
GAATTTATGGTGCACTCTCAGTACAATCTGCTCTGATGCCGCATAGTTAAGCCAGCCCCGACACCCGC
CAACACCCGCTGACGCGCCCTGACGGGCTTGTCTGCTCCCGGCATCCGCTTACAGACAAGCTGTGACC
GTCTCCGGGAGCTGCATGTGTCAGAGGTTTTCACCGTCATCACCGAAACGCGCGAGACGAAAGGGCCT
CGTGATACGCCTATTTTTATAGGTTAATGTCATGATAATAATGGTTTCTTAGACGTGCGGCCGCTCTA
GAACTAGTGGATCAATTCCACGGACTATAGACTATACTAGTATACTCCGTCTACTGTACGATACACTT
CCGCTCAGGTCCTTGTCCTTTAACGAGGCCTTACCACTCTTTTGTTACTCTATTGATCCAGCTCAGCA
AAGGCAGTGTGATCTAAGATTCTATCTTCGCGATGTAGTAAAACTAGCTAGACCGAGAAAGAGACTAG
AAATGCAAAAGGCACTTCTACAATGGCTGCCATCATTATTATCCGATGTGACGCTGCAGCTTCTCAAT
GATATTCGAATACGCTTTGAGGAGATACAGCCTAATATCCGACAAACTGTTTTACAGATTTACGATCG
TACTTGTTACCCATCATTGAATTTTGAACATCCGAACCTGGGAGTTTTCCCTGAAACAGATAGTATAT
TTGAACCTGTATAATAATATATAGTCTAGCGCTTTACGGAAGACAATGTATGTATTTCGGTTCCTGGA
GAAACTATTGCATCTATTGCATAGGTAATCTTGCACGTCGCATCCCCGGTTCATTTTCTGCGTTTCCA
TCTTGCACTTCAATAGCATATCTTTGTTAACGAAGCATCTGTGCTTCATTTTGTAGAACAAAAATGCA
ACGCGAGAGCGCTAATTTTTCAAACAAAGAATCTGAGCTGCATTTTTACAGAACAGAAATGCAACGCG
AAAGCGCTATTTTACCAACGAAGAATCTGTGCTTCATTTTTGTAAAACAAAAATGCAACGCGAGAGCG
CTAATTTTTCAAACAAAGAATCTGAGCTGCATTTTTACAGAACAGAAATGCAACGCGAGAGCGCTATT
TTACCAACAAAGAATCTATACTTCTTTTTTGTTCTACAAAAATGCATCCCGAGAGCGCTATTTTTCTA
ACAAAGCATCTTAGATTACTTTTTTTCTCCTTTGTGCGCTCTATAATGCAGTCTCTTGATAACTTTTT
GCACTGTAGGTCCGTTAAGGTTAGAAGAAGGCTACTTTGGTGTCTATTTTCTCTTCCATAAAAAAAGC
CTGACTCCACTTCCCGCGTTTACTGATTACTAGCGAAGCTGCGGGTGCATTTTTTCAAGATAAAGGCA

TCCCCGATTATATTCTATACCGATGTGGATTGCGCATACTTTGTGAACAGAAAGTGATAGCGTTGATG
ATTCTTCATTGGTCAGAAAATTATGAACGGTTTCTTCTATTTTGTCTCTATATACTACGTATAGGAAA
TGTTTACATTTTCGTATTGTTTTCGATTCACTCTATGAATAGTTCTTACTACAATTTTTTTGTCTAAA
GAGTAATACTAGAGATAAACATAAAAAATGTAGAGGTCGAGTTTAGATGCAAGTTCAAGGAGCGAAAG
GTGGATGGGTAGGTTATATAGGGATATAGCACAGAGATATATAGCAAAGAGATACTTTTGAGCAATGT
TTGTGGAAGCGGTATTCGCAATATTTTAGTAGCTCGTTACAGTCCGGTGCGTTTTTGGTTTTTTGAAA
GTGCGTCTTCAGAGCGCTTTTGGTTTTCAAAAGCGCTCTGAAGTTCCTATACTTTCTAGAGAATAGGA
ACTTCGGAATAGGAACTTCAAAGCGTTTCCGAAAACGAGCGCTTCCGAAAATGCAACGCGAGCTGCGC
ACATACAGCTCACTGTTCACGTCGCACCTATATCTGCGTGTTGCCTGTATATATATATACATGAGAAG
AACGGCATAGTGCGTGTTTATGCTTAAATGCGTACTTATATGCGTCTATTTATGTAGGATGAAAGGTA
GTCTAGTACCTCCTGTGATATTATCCCATTCCATGCGGGGTATCGTATGCTTCCTTCAGCACTACCCT
TTAGCTGTTCTATATGCTGCCACTCCTCAATTGGATTAGTCTCATCCTTCAATGCTATCATTTCCTTT
GATATTGGATCATATGCATAGTACCGAGAAACTAGTGCGAAGTAGTGATCAGGTATTGCTGTTATCTG
ATGAGTATACGTTGTCCTGGCCACGGCAGAAGCACGCTTATCGCTCCAATTTCCCACAACATTAGTCA
ACTCCGTTAGGCCCTTCATTGAAAGAAATGAGGTCATCAAATGTCTTCCAATGTGAGATTTTGGGCCA
TTTTTTATAGCAAAGATTGAATAAGGCGCATTTTTCTTCAAAGCTGCGGCCGCACGTCAGGTGGCACT
TTTCGGGGAAATGTGCGCGGAACCCCTATTTGTTTATTTTTCTAAATACATTCAAATATGTATCCGCT
CATGAGACAATAACCGTGATAAATGCTTCAATAATATTGAAAAAGGAAGAGTATGAGTATTCAACATT
TCCGTGTCGCCCTTATTCCCTTTTTTGCGGCATTTTGCCTTCCTGTTTTTGCTCACCCAGAAACGCTG
GTGAAAGTAAAAGATGCTGAAGATCAGTTGGGTGCACGAGTGGGTTACATCGAACTGGATCTCAACAG
CGGTAAGATCCTTGAGAGTTTTCGCCCCGAAGAACGTTTTCCAATGATGAGCACTTTTAAAGTTCTGC
TATGTGGCGCGGTATTATCCCGTATTGACGCCGGGCAAGAGCAACTCGGTCGCCGCATACACTATTCT
CAGAATGACTTGGTTGAGTACTCACCAGTCACAGAAAAGCATCTTACGGATGGCATGACAGTAAGAGA
ATTATGCAGTGCTGCCATAACCATGAGTGATAACACTGCGGCCAACTTACTTCTGACAACGATCGGAG
GACCGAAGGAGCTAACCGCTTTTTTGCACAACATGGGGGATCATGTAACTCGCCTTGATCGTTGGGAA
CCGGAGCTGAATGAAGCCATACCAAACGACGAGCGTGACACCACGATGCCTGTAGCAATGGCAACAAC
GTTGCGCAAACTATTAACTGGCGAACTACTTACTCTAGCTTCCCGGCAACAATTAATAGACTGGATGG
AGGCGGATAAAGTTGCAGGACCACTTCTGCGCTCGGCCCTTCCGGCTGGCTGGTTTATTGCTGATAAA
TCTGGAGCCGGTGAGCGTGGGTCTCGCGGTATCATTGCAGCACTGGGGCCAGATGGTAAGCCCTCCCG
TATCGTAGTTATCTACACGACGGGGAGTCAGGCAACTATGGATGAACGAAATAGACAGATCGCTGAGA
TAGGTGCCTCACTGATTAAGCATTGGTAACTGTCAGACCAAGTTTACTCATATATACTTTAGATTGAT
TTAAAACTTCATTTTTAATTTAAAAGGATCTAGGTGAAGATCCTTTTTGATAATCTCATGACCAAAAT
CCCTTAACGTGAGTTTTCGTTCCACTGAGCGTCAGACCCCGTAGAAAAGATCAAAGGATCTTCTTGAG
ATCCTTTTTTTCTGCGCGTAATCTGCTGCTTGCAAACAAAAAAACCACCGCTACCAGCGGTGGTTTGT
TTGCCGGATCAAGAGCTACCAACTCTTTTTCCGAAGGTAACTGGCTTCAGCAGAGCGCAGATACCAAA
TACTGTTCTTCTAGTGTAGCCGTAGTTAGGCCACCACTTCAAGAACTCTGTAGCACCGCCTACATACC
TCGCTCTGCTAATCCTGTTACCAGTGGCTGCTGCCAGTGGCGATAAGTCGTGTCTTACCGGGTTGGAC
TCAAGACGATAGTTACCGGATAAGGCGCAGCGGTCGGGCTGAACGGGGGGTTCGTGCACACAGCCCAG
CTTGGAGCGAACGACCTACACCGAACTGAGATACCTACAGCGTGAGCTATGAGAAAGCGCCACGCTTC
CCGAAGGGAGAAAGGCGGACAGGTATCCGGTAAGCGGCAGGGTCGGAACAGGAGAGCGCACGAGGGAG
CTTCCAGGGGGAAACGCCTGGTATCTTTATAGTCCTGTCGGGTTTCGCCACCTCTGACTTGAGCGTCG
ATTTTTGTGATGCTCGTCAGGGGGGCGGAGCCTATGGAAAAACGCCAGCAACGCGGCCTTTTTACGGT
TCCTGGCCTTTTGCTGGCCTTTTGCTCACATGTTCTTTCCTGCGTTATCCCCTGATTCTGTGGATAAC
CGTATTACCGCCTTTGAGTGAGCTGATACCGCTCGCCGCAGCCGAACGACCGAGCGCAGCGAGTCAGT
GAGCGAGGAAGCGGAAGAGCGCCCAATACGCAAACCGCCTCTCCCCGCGCGTTGGCCGATTCATTAAT
GCAGCTGGCACGACAGGTTTCCCGACTGGAAAGCGGGCAGTGAGCGCAACGCAATTAATGTGAGTTAG
CTCACTCATTAGGCACCCCAGGCTTTACACTTTATGCTTCCGGCTCGTATGTTGTGTGGAATTGTGAG
CGGATAACAATTTCACACAGGAAACAGCTATGACCATGATTACGCCAAGCTCGAAATTAACCCTCACT

AAAGGGAACAAAAGCTGGTACCGGGCCGGCCGTCGGGCCGTCGAGCTTGATGGCATCGTGGTGTCACG
CTCGTCGTTTGGTATGGCTTCATTCAGCTCCGGTTCCCAACGATCAAGGCGAGTTACATGATCCCCCA
TGTTGTGCAAAAAAGCGGTTAGCTCCTTCGGTCCTCCGATCGTTGTCAGAAGTAAGTTGGCCGCAGTG
TTATCACTCATGGTTATGGCAGCACTGCATAATTCTCTTACTGTCATGCCATCCGTAAGATGCTTTTC
TGTGACTGGTGAGTACTCAACCAAGTCATTCTGAGAATAGTGTATGCGGCGACCGAGTTGCTCTTGCC
CGGCGTCAACACGGGATAATACCGCGCCACATAGCAGAACTTTAAAAGTGCTCATCATTGGAAAACGT
TCTTCGGGGCGAAAACTCTCAAGGATCTTACCGCTGTTGAGATCCAGTTCGATGTAACCCACTCGTGC
ACCCAACTGATCTTCAGCATCTTTTACTTTCACCAGCGTTTCTGGGTGAGCAAAAACAGGAAGGCAAA
ATGCCGCAAAAAAGGGAATAAGGGCGACACGGAAATGTTGAATACTCATACTCTTCCTTTTTCAATAT
TATTGAAGCATTTATCAGGGTTATTGTCTCATGAGCGGATACATATTTGAATGTATTTAGAAAAATAA
ACAAATAGGGGTTCCGCGCACATTTCCCCGAAAAGTGCCACCTGACGTCTAAGAAACCATTATTATCA
TGACATTAACCTATAAAAATAGGCGTATCACGAGGCCCTTTCGTCTTCAAGAATTGGGGATCTACGTA
TGGTCATTTCTTCTTCAGATTCCCTCATGGAGAAAGTGCGGCAGATGTATATGACAGAGTCGCCAGTT
TCCAAGAGACTTTATTCAGGCACTTCCATGATAGGCAAGAGAGAAGACCCAGAGATGTTGTTGTCCTA
GTTACACATGGTATTTATTCCAGAGTATTCCTGATGAAATGGTTTAGATGGACATACGAAGAGTTTGA
ATCGTTTACCAATGTTCCTAACGGGAGCGTAATGGTGATGGAACTGGACGAATCCATCAATAGATACG
TCCTGAGGACCGTGCTACCCAAATGGACTGATTGTGAGGGAGACCTAACTACATAGTGTTTAAAGATT
ACGGATATTTAACTTACTTAGAATAATGCCATTTTTTTGAGTTATAATAATCCTACGTTAGTGTGAGC
GGGATTTAAACTGTGAGGACCTTAATACATTCAGACACTTCTGCGGTATCACCCTACTTATTCCCTTC
GAGATTATATCTAGGAACCCATCAGGTTGGTGGAAGATTACCCGTTCTAAGACTTTTCAGCTTCCTCT
ATTGATGTTACACCTGGACACCCCTTTTCTGGCATCCAGTTTTTAATCTTCAGTGGCATGTGAGATTC
TCCGAAATTAATTAAAGCAATCACACAATTCTCTCGGATACCACCTCGGTTGAAACTGACAGGTGGTT
TGTTACGCATGCTAATGCAAAGGAGCCTATATACCTTTGGCTCGGCTGCTGTAACAGGGAATATAAAG
GGCAGCATAATTTAGGAGTTTAGTGAACTTGCAACATTTACTATTTTCCCTTCTTACGTAAATATTTT
TCTTTTTAATTCTAAATCAATCTTTTTCAATTTTTTGTTTGTATTCTTTTCTTGCTTAAATCTATAAC
TACAAAAAACACATACAG

Figure S8: Vector map and nucleotide sequence of expression plasmid, pYX222-HRP

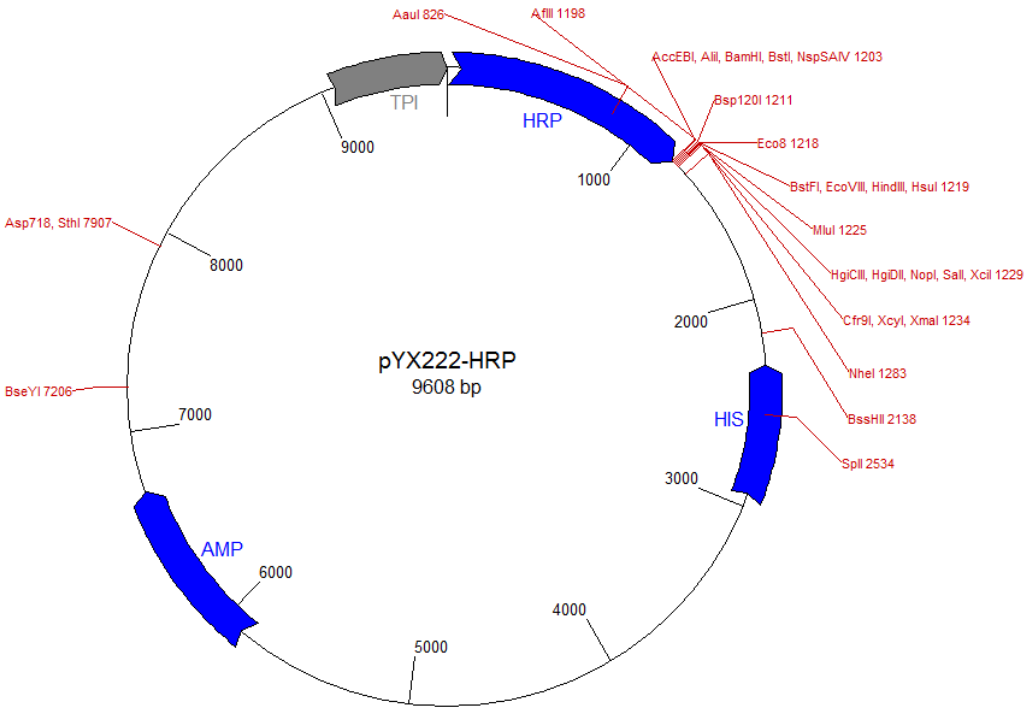

CCGAAACGATGAGATTCCCATCTATTTTCACCGCTGTCTTGTTCGCTGCCTCCTCTGCATTGGCTGCC
CCTGTTAACACTACCACTGAAGACGAGACTGCTCAAATTCCAGCTGAAGCAGTTATCGGTTACTCTGA
CCTTGAGGGTGATTTCGACGTCGCTGTTTTGCCTTTCTCTAACTCCACTAACAACGGTTTGTTGTTCA
TTAACACCACTATCGCTTCCATTGCTGCTAAGGAAGAGGGTGTCTCTCTCGAGAAGAGAGAGGCCGAA
GCTCAATTGACTCCTACTTTCTACGACAACTCTTGTCCAAACGTTTCTAACATCGTTAGAGATACCAT
TGTCAACGAGTTGAGATCCGACCCAAGAATTGCTGCATCTATCCTGAGATTGCACTTCCACGACTGTT
TCGTTAACGGTTGTGACGCTTCTATCCTGTTGGATAACACCACTTCTTTCAGAACTGAGAAGGATGCT
TTCGGTAACGCTAACTCCGCTAGAGGTTTTCCTGTCATTGACAGAATGAAGGCAGCTGTTGAATCTGC
TTGTCCTAGAACCGTCTCTTGTGCTGACTTGTTGACCATTGCTGCACAACAGTCCGTTACCTTGGCTG
GTGGTCCATCTTGGAGAGTTCCTCTTGGTAGAAGAGACTCTTTGCAGGCTTTCTTGGACCTTGCTAAC
GCTAACTTGCCAGCACCTTTCTTCACTTTGCCACAATTGAAGGACTCTTTCCGTAACGTTGGTTTGAA
CAGATCCTCTGACCTTGTTGCTTTGTCTGGAGGTCACACCTTCGGTAAGAACCAATGTAGATTCATCA
TGGATAGATTGTACAACTTCTCCAACACTGGTCTTCCAGATCCAACCCTTAACACCACTTACCTTCAA
ACTTTGAGAGGTTTGTGCCCTTTGAACGGTAACTTGTCTGCTCTTGTTGACTTCGACTTGAGAACTCC
TACCATCTTCGACAACAAGTACTACGTCAACCTTGAGGAACAGAAGGGATTGATCCAATCTGACCAAG
AGTTGTTCTCCTCTCCTAACGCTACTGACACTATTCCTCTTGTCAGATCCTTCGCTAACTCTACCCAA
ACCTTCTTCAACGCCTTCGTTGAAGCTATGGACAGAATGGGTAACATTACTCCACTTACTGGTACTCA
AGGTCAGATCAGATTGAACTGTAGAGTCGTTAACTCCAACTCTTAAGGATCCTAGGGCCCACAAGCTT
ACGCGTCGACCCGGGTATCCGTATGATGTGCCTGACTACGCATGATATCTCGAGCTCAGCTAGCTAAC
TGAATAAGGAACAATGAACGTTTTTCCTTTCTCTTGTTCCTAGTATTAATGACTGACCGATACATCCC
TTTTTTTTTTTGTCTTTGTCTAGCTCCAATTCGCCCTATAGTGAGTCGTATTACAATTCACTGGCCGT

CGTTTTACAACGTCGTGACTGGGAAAACCCTGGCGTTACCCAACTTAATCGCCTTGCAGCACATCCCC
CTTTCGCCAGCTGGCGTAATAGCGAAGAGGCCCGCACCGATCGCCCTTCCCAACAGTTGCGCAGCCTG
AATGGCGAATGGACGCGCCCTGTAGCGGCGCATTAAGCGCGGCGGGTGTGGTGGTTACGCGCAGCGTG
ACCGCTACACTTGCCAGCGCCCTAGCGCCCGCTCCTTTCGCTTTCTTCCCTTCCTTTCTCGCCACGTT
CGCCGGCTTTCCCCGTCAAGCTCTAAATCGGGGGCTCCCTTTAGGGTTCCGATTTAGTGCTTTACGGC
ACCTCGACCCCAAAAAACTTGATTAGGGTGATGGTTCACGTAGTGGGCCATCGCCCTGATAGACGGTT
TTTCGCCCTTTGACGTTGGAGTCCACGTTCTTTAATAGTGGACTCTTGTTCCAAACTGGAACAACACT
CAACCCTATCTCGGTCTATTCTTTTGATTTATAAGGGATTTTGCCGATTTCGGCCTATTGGTTAAAAA
ATGAGCTGATTTAACAAAAATTTAACGCGAATTTTAACAAAATATTAACGCTTACAATTTCCTGATGC
GGTATTTTCTCCTTACGCATCTGTGCGGTATTTCACACCGCATAGATCCGTCGAGTTCAAGAGAAAAA
AAAAGAAAAAGCAAAAAGAAAAAAGGAAAGCGCGCCTCGTTCAGAATGACACGTATAGAATGATGCAT
TACCTTGTCATCTTCAGTATCATACTGTTCGTATACATACTTACTGACATTCATAGGTATACATATAT
ACACATGTATATATATCGTATGCTGCAGCTTTAAATAATCGGTGTCACTACATAAGAACACCTTTGGT
GGAGGGAACATCGTTGGTTCCATTGGGCGAGGTGGCTTCTCTTATGGCAACCGCAAGAGCCTTGAACG
CACTCTCACTACGGTGATGATCATTCTTGCCTCGCAGACAATCAACGTGGAGGGTAATTCTGCTTGCC
TCTGCAAAACTTTCAAGAAAATGCGGGATCATCTCGCAAGAGAGATCTCCTACTTTCTCCCTCTGCAA
ACCAAGTTCGACAACTGCGTACGGCCTGTTCGAAAGATCTACCACCGCTCTGGAAAGTGCCTCATCCA
AAGGCGCAAATCCTGATCCAAACCTTTTTACTCCACGCACGGCCCCTAGGGCCTCTTTAAATGCTTGA
CCGAGAGCAATCCCGCAGTCTTCAGTGGTGTGATGGTCGTCTATGTGTAAGTCACCAATGCACTCAAC
GATTAGCGACCAGCCGGAATGCTTGGCCAGAGCATGTATCATATGGTCCAGAAACCCTATACCTGTGT
GGACGTTAATCACTTGCGATTGTGTGGCCTGTTCTGCTACTGCTTCTGCCTCTTTTTCTGGGAAGATC
GAGTGCTCTATCGCTAGGGGACCACCCTTTAAAGAGATCGCAATCTGAATCTTGGTTTCATTTGTAAT
ACGCTTTACTAGGGCTTTCTGCTCTGTCATCTTTGCCTTCGTTTATCTTGCCTGCTCATTTTTTAGTA
TATTCTTCGAAGAAATCACATTACTTTATATAATGTATAATTCATTATGTGATAATGCCAATCGCTAA
GAAAAAAAAAGAGTCATCCGCTAGGGGAAAAAAAAAAAATGAAAATCATTACCGAGGCATAAAAAAATA
TAGAGTGTACTAGAGGAGGCCAAGAGTAATAGAAAAAGAAAATTGCGGGAAAGGACTGTGTTATGACT
TCCCTGACTAATGCCGTGTTCAAACGATACCTGGCAGTGACTCCTAGCGCTCACCAAGCTCTTAAAAC
GGGAATTTATGGTGCACTCTCAGTACAATCTGCTCTGATGCCGCATAGTTAAGCCAGCCCCGACACCC
GCCAACACCCGCTGACGCGCCCTGACGGGCTTGTCTGCTCCCGGCATCCGCTTACAGACAAGCTGTGA
CCGTCTCCGGGAGCTGCATGTGTCAGAGGTTTTCACCGTCATCACCGAAACGCGCGAGACGAAAGGGC
CTCGTGATACGCCTATTTTTATAGGTTAATGTCATGATAATAATGGTTTCTTAGACGTGCGGCCGCTC
TAGAACTAGTGGATCAATTCCACGGACTATAGACTATACTAGTATACTCCGTCTACTGTACGATACAC
TTCCGCTCAGGTCCTTGTCCTTTAACGAGGCCTTACCACTCTTTTGTTACTCTATTGATCCAGCTCAG
CAAAGGCAGTGTGATCTAAGATTCTATCTTCGCGATGTAGTAAAACTAGCTAGACCGAGAAAGAGACT
AGAAATGCAAAAGGCACTTCTACAATGGCTGCCATCATTATTATCCGATGTGACGCTGCAGCTTCTCA
ATGATATTCGAATACGCTTTGAGGAGATACAGCCTAATATCCGACAAACTGTTTTACAGATTTACGAT
CGTACTTGTTACCCATCATTGAATTTTGAACATCCGAACCTGGGAGTTTTCCCTGAAACAGATAGTAT
ATTTGAACCTGTATAATAATATATAGTCTAGCGCTTTACGGAAGACAATGTATGTATTTCGGTTCCTG
GAGAAACTATTGCATCTATTGCATAGGTAATCTTGCACGTCGCATCCCCGGTTCATTTTCTGCGTTTC
CATCTTGCACTTCAATAGCATATCTTTGTTAACGAAGCATCTGTGCTTCATTTTGTAGAACAAAAATG
CAACGCGAGAGCGCTAATTTTTCAAACAAAGAATCTGAGCTGCATTTTTACAGAACAGAAATGCAACG
CGAAAGCGCTATTTTACCAACGAAGAATCTGTGCTTCATTTTTGTAAAACAAAAATGCAACGCGAGAG
CGCTAATTTTTCAAACAAAGAATCTGAGCTGCATTTTTACAGAACAGAAATGCAACGCGAGAGCGCTA
TTTTACCAACAAAGAATCTATACTTCTTTTTTGTTCTACAAAAATGCATCCCGAGAGCGCTATTTTTC
TAACAAAGCATCTTAGATTACTTTTTTTCTCCTTTGTGCGCTCTATAATGCAGTCTCTTGATAACTTT
TTGCACTGTAGGTCCGTTAAGGTTAGAAGAAGGCTACTTTGGTGTCTATTTTCTCTTCCATAAAAAAA
GCCTGACTCCACTTCCCGCGTTTACTGATTACTAGCGAAGCTGCGGGTGCATTTTTTCAAGATAAAGG
CATCCCCGATTATATTCTATACCGATGTGGATTGCGCATACTTTGTGAACAGAAAGTGATAGCGTTGA

TGATTCTTCATTGGTCAGAAAATTATGAACGGTTTCTTCTATTTTGTCTCTATATACTACGTATAGGA
AATGTTTACATTTTCGTATTGTTTTCGATTCACTCTATGAATAGTTCTTACTACAATTTTTTTGTCTA
AAGAGTAATACTAGAGATAAACATAAAAAATGTAGAGGTCGAGTTTAGATGCAAGTTCAAGGAGCGAA
AGGTGGATGGGTAGGTTATATAGGGATATAGCACAGAGATATATAGCAAAGAGATACTTTTGAGCAAT
GTTTGTGGAAGCGGTATTCGCAATATTTTAGTAGCTCGTTACAGTCCGGTGCGTTTTTGGTTTTTTGA
AAGTGCGTCTTCAGAGCGCTTTTGGTTTTCAAAAGCGCTCTGAAGTTCCTATACTTTCTAGAGAATAG
GAACTTCGGAATAGGAACTTCAAAGCGTTTCCGAAAACGAGCGCTTCCGAAAATGCAACGCGAGCTGC
GCACATACAGCTCACTGTTCACGTCGCACCTATATCTGCGTGTTGCCTGTATATATATATACATGAGA
AGAACGGCATAGTGCGTGTTTATGCTTAAATGCGTACTTATATGCGTCTATTTATGTAGGATGAAAGG
TAGTCTAGTACCTCCTGTGATATTATCCCATTCCATGCGGGGTATCGTATGCTTCCTTCAGCACTACC
CTTTAGCTGTTCTATATGCTGCCACTCCTCAATTGGATTAGTCTCATCCTTCAATGCTATCATTTCCT
TTGATATTGGATCATATGCATAGTACCGAGAAACTAGTGCGAAGTAGTGATCAGGTATTGCTGTTATC
TGATGAGTATACGTTGTCCTGGCCACGGCAGAAGCACGCTTATCGCTCCAATTTCCCACAACATTAGT
CAACTCCGTTAGGCCCTTCATTGAAAGAAATGAGGTCATCAAATGTCTTCCAATGTGAGATTTTGGGC
CATTTTTTATAGCAAAGATTGAATAAGGCGCATTTTTCTTCAAAGCTGCGGCCGCACGTCAGGTGGCA
CTTTTCGGGGAAATGTGCGCGGAACCCCTATTTGTTTATTTTTCTAAATACATTCAAATATGTATCCG
CTCATGAGACAATAACCGTGATAAATGCTTCAATAATATTGAAAAAGGAAGAGTATGAGTATTCAACA
TTTCCGTGTCGCCCTTATTCCCTTTTTTGCGGCATTTTGCCTTCCTGTTTTTGCTCACCCAGAAACGC
TGGTGAAAGTAAAAGATGCTGAAGATCAGTTGGGTGCACGAGTGGGTTACATCGAACTGGATCTCAAC
AGCGGTAAGATCCTTGAGAGTTTTCGCCCCGAAGAACGTTTTCCAATGATGAGCACTTTTAAAGTTCT
GCTATGTGGCGCGGTATTATCCCGTATTGACGCCGGGCAAGAGCAACTCGGTCGCCGCATACACTATT
CTCAGAATGACTTGGTTGAGTACTCACCAGTCACAGAAAAGCATCTTACGGATGGCATGACAGTAAGA
GAATTATGCAGTGCTGCCATAACCATGAGTGATAACACTGCGGCCAACTTACTTCTGACAACGATCGG
AGGACCGAAGGAGCTAACCGCTTTTTTGCACAACATGGGGGATCATGTAACTCGCCTTGATCGTTGGG
AACCGGAGCTGAATGAAGCCATACCAAACGACGAGCGTGACACCACGATGCCTGTAGCAATGGCAACA
ACGTTGCGCAAACTATTAACTGGCGAACTACTTACTCTAGCTTCCCGGCAACAATTAATAGACTGGAT
GGAGGCGGATAAAGTTGCAGGACCACTTCTGCGCTCGGCCCTTCCGGCTGGCTGGTTTATTGCTGATA
AATCTGGAGCCGGTGAGCGTGGGTCTCGCGGTATCATTGCAGCACTGGGGCCAGATGGTAAGCCCTCC
CGTATCGTAGTTATCTACACGACGGGGAGTCAGGCAACTATGGATGAACGAAATAGACAGATCGCTGA
GATAGGTGCCTCACTGATTAAGCATTGGTAACTGTCAGACCAAGTTTACTCATATATACTTTAGATTG
ATTTAAAACTTCATTTTTAATTTAAAAGGATCTAGGTGAAGATCCTTTTTGATAATCTCATGACCAAA
ATCCCTTAACGTGAGTTTTCGTTCCACTGAGCGTCAGACCCCGTAGAAAAGATCAAAGGATCTTCTTG
AGATCCTTTTTTTCTGCGCGTAATCTGCTGCTTGCAAACAAAAAAACCACCGCTACCAGCGGTGGTTT
GTTTGCCGGATCAAGAGCTACCAACTCTTTTTCCGAAGGTAACTGGCTTCAGCAGAGCGCAGATACCA
AATACTGTTCTTCTAGTGTAGCCGTAGTTAGGCCACCACTTCAAGAACTCTGTAGCACCGCCTACATA
CCTCGCTCTGCTAATCCTGTTACCAGTGGCTGCTGCCAGTGGCGATAAGTCGTGTCTTACCGGGTTGG
ACTCAAGACGATAGTTACCGGATAAGGCGCAGCGGTCGGGCTGAACGGGGGGTTCGTGCACACAGCCC
AGCTTGGAGCGAACGACCTACACCGAACTGAGATACCTACAGCGTGAGCTATGAGAAAGCGCCACGCT
TCCCGAAGGGAGAAAGGCGGACAGGTATCCGGTAAGCGGCAGGGTCGGAACAGGAGAGCGCACGAGGG
AGCTTCCAGGGGGAAACGCCTGGTATCTTTATAGTCCTGTCGGGTTTCGCCACCTCTGACTTGAGCGT
CGATTTTTGTGATGCTCGTCAGGGGGGCGGAGCCTATGGAAAAACGCCAGCAACGCGGCCTTTTTACG
GTTCCTGGCCTTTTGCTGGCCTTTTGCTCACATGTTCTTTCCTGCGTTATCCCCTGATTCTGTGGATA
ACCGTATTACCGCCTTTGAGTGAGCTGATACCGCTCGCCGCAGCCGAACGACCGAGCGCAGCGAGTCA
GTGAGCGAGGAAGCGGAAGAGCGCCCAATACGCAAACCGCCTCTCCCCGCGCGTTGGCCGATTCATTA
ATGCAGCTGGCACGACAGGTTTCCCGACTGGAAAGCGGGCAGTGAGCGCAACGCAATTAATGTGAGTT
AGCTCACTCATTAGGCACCCCAGGCTTTACACTTTATGCTTCCGGCTCGTATGTTGTGTGGAATTGTG
AGCGGATAACAATTTCACACAGGAAACAGCTATGACCATGATTACGCCAAGCTCGAAATTAACCCTCA
CTAAAGGGAACAAAAGCTGGTACCGGGCCGGCCGTCGGGCCGTCGAGCTTGATGGCATCGTGGTGTCA

CGCTCGTCGTTTGGTATGGCTTCATTCAGCTCCGGTTCCCAACGATCAAGGCGAGTTACATGATCCCC
CATGTTGTGCAAAAAAGCGGTTAGCTCCTTCGGTCCTCCGATCGTTGTCAGAAGTAAGTTGGCCGCAG
TGTTATCACTCATGGTTATGGCAGCACTGCATAATTCTCTTACTGTCATGCCATCCGTAAGATGCTTT
TCTGTGACTGGTGAGTACTCAACCAAGTCATTCTGAGAATAGTGTATGCGGCGACCGAGTTGCTCTTG
CCCGGCGTCAACACGGGATAATACCGCGCCACATAGCAGAACTTTAAAAGTGCTCATCATTGGAAAAC
GTTCTTCGGGGCGAAAACTCTCAAGGATCTTACCGCTGTTGAGATCCAGTTCGATGTAACCCACTCGT
GCACCCAACTGATCTTCAGCATCTTTTACTTTCACCAGCGTTTCTGGGTGAGCAAAAACAGGAAGGCA
AAATGCCGCAAAAAAGGGAATAAGGGCGACACGGAAATGTTGAATACTCATACTCTTCCTTTTTCAAT
ATTATTGAAGCATTTATCAGGGTTATTGTCTCATGAGCGGATACATATTTGAATGTATTTAGAAAAAT
AAACAAATAGGGGTTCCGCGCACATTTCCCCGAAAAGTGCCACCTGACGTCTAAGAAACCATTATTAT
CATGACATTAACCTATAAAAATAGGCGTATCACGAGGCCCTTTCGTCTTCAAGAATTGGGGATCTACG
TATGGTCATTTCTTCTTCAGATTCCCTCATGGAGAAAGTGCGGCAGATGTATATGACAGAGTCGCCAG
TTTCCAAGAGACTTTATTCAGGCACTTCCATGATAGGCAAGAGAGAAGACCCAGAGATGTTGTTGTCC
TAGTTACACATGGTATTTATTCCAGAGTATTCCTGATGAAATGGTTTAGATGGACATACGAAGAGTTT
GAATCGTTTACCAATGTTCCTAACGGGAGCGTAATGGTGATGGAACTGGACGAATCCATCAATAGATA
CGTCCTGAGGACCGTGCTACCCAAATGGACTGATTGTGAGGGAGACCTAACTACATAGTGTTTAAAGA
TTACGGATATTTAACTTACTTAGAATAATGCCATTTTTTTGAGTTATAATAATCCTACGTTAGTGTGA
GCGGGATTTAAACTGTGAGGACCTTAATACATTCAGACACTTCTGCGGTATCACCCTACTTATTCCCT
TCGAGATTATATCTAGGAACCCATCAGGTTGGTGGAAGATTACCCGTTCTAAGACTTTTCAGCTTCCT
CTATTGATGTTACACCTGGACACCCCTTTTCTGGCATCCAGTTTTTAATCTTCAGTGGCATGTGAGAT
TCTCCGAAATTAATTAAAGCAATCACACAATTCTCTCGGATACCACCTCGGTTGAAACTGACAGGTGG
TTTGTTACGCATGCTAATGCAAAGGAGCCTATATACCTTTGGCTCGGCTGCTGTAACAGGGAATATAA
AGGGCAGCATAATTTAGGAGTTTAGTGAACTTGCAACATTTACTATTTTCCCTTCTTACGTAAATATT
TTTCTTTTTAATTCTAAATCAATCTTTTTCAATTTTTTGTTTGTATTCTTTTCTTGCTTAAATCTATA
ACTACAAAAAACACATACAG

Figure S9: Vector map and nucleotide sequence of expression plasmid, pYX222-alphaSS-A$_{2a}$, where alpha SS is the *S. cerevisiae* mating factor α secretion signal

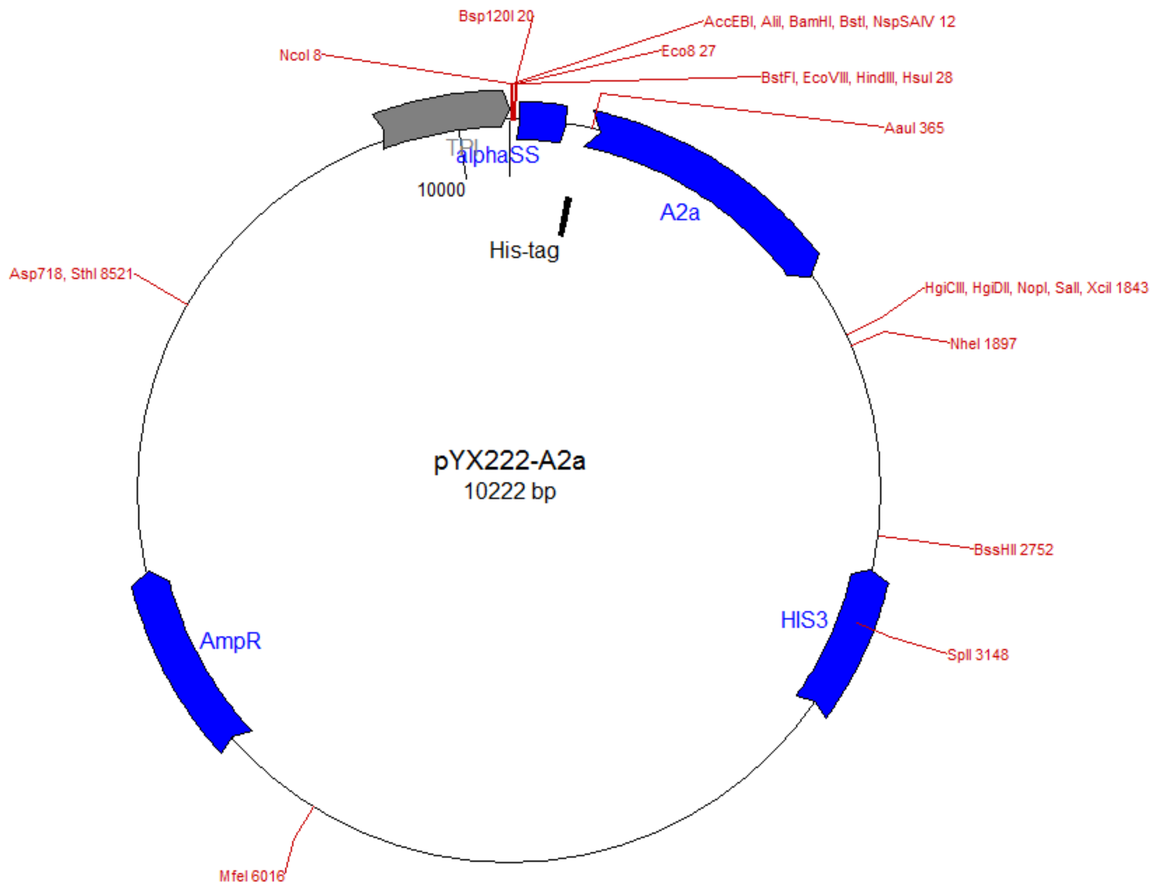

GAATTCACCATGGATCCTAGGGCCCACAAGCTTAACAAAATGAGATTTCCTTCAATTTTTACTGCAGT
TTTATTCGCAGCATCCTCCGCATTAGCTGCTCCAGTCAACACTACAACAGAAGATGAAACGGCACAAA
TTCCGGCTGAAGCTGTCATCGGTTACTCAGATTTAGAAGGGGATTTCGATGTTGCTGTTTTGCCATTT
TCCAACAGCACAAATAACGGGTTATTGTTTATAAATACTACTATTGCCAGCATTGCTGCTAAAGAAGA
AGGGGTATCTCTCGAGAAAAGAGAGGCTGAAGCTCATCATCATCATCATCATCATCATCATCATGAAT
TCATGCCCATCATGGGCTCCTCGGTGTACATCACGGTGGAGCTGGCCATTGCTGTGCTGGCCATCCTG
GGCAATGTGCTGGTGTGCTGGGCCGTGTGGCTCAACAGCAACCTGCAGAACGTCACCAACTACTTTGT
GGTGTCACTGGCGGCGGCCGACATCGCAGTGGGTGTGCTCGCCATCCCCTTTGCCATCACCATCAGCA
CCGGGTTCTGCGCTGCCTGCCACGGCTGCCTCTTCATTGCCTGCTTCGTCCTGGTCCTCACGCAGAGC
TCCATCTTCAGTCTCCTGGCCATCGCCATTGACCGCTACATTGCCATCCGCATCCCGCTCCGGTACAA
TGGCTTGGTGACCGGCACGAGGGCTAAGGGCATCATTGCCATCTGCTGGGTGCTGTCGTTTGCCATCG
GCCTGACTCCCATGCTAGGTTGGAACAACTGCGGTCAGCCAAAGGAGGGCAAGCAGCACTCCCAGGGC
TGCGGGGAGGGCCAAGTGGCCTGTCTCTTTGAGGATGTGGTCCCCATGAACTACATGGTGTACTTCAA
CTTCTTTGCCTGTGTGCTGGTGCCCCTGCTGCTCATGCTGGGTGTCTATTTGCGGATCTTCCTGGCGG
CGCGACGACAGCTGAAGCAGATGGAGAGCCAGCCTCTGCCGGGGGAGCGGGCACGGTCCACACTGCAG
AAGGAGGTCCATGCTGCCAAGTCACTGGCCATCATTGTGGGGCTCTTTGCCCTCTGCTGGCTGCCCCT
ACACATCATCAACTGCTTCACTTTCTTCTGCCCCGACTGCAGCCACGCCCCTCTCTGGCTCATGTACC
TGGCCATCGTCCTCTCCCACACCAATTCGGTTGTGAATCCCTTCATCTACGCCTACCGTATCCGCGAG
TTCCGCCAGACCTTCCGCAAGATCATTCGCAGCCACGTCCTGAGGCAGCAAGAACCTTTCAAGGCAGC

TGGCACCAGTGCCCGGGTCTTGGCAGCTCATGGCAGTGACGGAGAGCAGGTCAGCCTCCGTCTCAACG
GCCACCCGCCAGGAGTGTGGGCCAACGGCAGTGCTCCCCACCCTGAGCGGAGGCCCAATGGCTACGCC
CTGGGGCTGGTGAGTGGAGGGAGTGCCCAAGAGTCCCAGGGGAACACGGGCCTCCCAGACGTGGAGCT
CCTTAGCCATGAGCTCAAGGGAGTGTGCCCAGAGCCCCCTGGCCTAGATGACCCCCTGGCCCAGGATG
GAGCAGGAGTGTCCGCGGCCGCTGAAAATCTGTATTTCCAGAGTGCCGGTAAGGCCGGAGAGGGCGAG
ATTCCCGCTCCGCTGGCCGGCACCGTCTCCAAGATCCTCGTGAAGGAGGGTGACACGGTCAAGGCTGG
TCAGACCGTGCTCGTTCTCGAGGCCATGAAGATGGAGACCGAGATCAACGCTCCCACCGACGGCAAGG
TCGAGAAGGTCCTGGTCAAGGAGCGTGACGCGGTGCAGGGCGGTCAGGGTCTCATCAAGATCGGGTGA
TCTAGAGTCGACCCGGGTATCCGTATGATGTGCCTGACTACGCATGATATCTCGAGCTCAGCTAGCTA
ACTGAATAAGGAACAATGAACGTTTTTCCTTTCTCTTGTTCCTAGTATTAATGACTGACCGATACATC
CCTTTTTTTTTTTGTCTTTGTCTAGCTCCAATTCGCCCTATAGTGAGTCGTATTACAATTCACTGGCC
GTCGTTTTACAACGTCGTGACTGGGAAAACCCTGGCGTTACCCAACTTAATCGCCTTGCAGCACATCC
CCCTTTCGCCAGCTGGCGTAATAGCGAAGAGGCCCGCACCGATCGCCCTTCCCAACAGTTGCGCAGCC
TGAATGGCGAATGGACGCGCCCTGTAGCGGCGCATTAAGCGCGGCGGGTGTGGTGGTTACGCGCAGCG
TGACCGCTACACTTGCCAGCGCCCTAGCGCCCGCTCCTTTCGCTTTCTTCCCTTCCTTTCTCGCCACG
TTCGCCGGCTTTCCCCGTCAAGCTCTAAATCGGGGGCTCCCTTTAGGGTTCCGATTTAGTGCTTTACG
GCACCTCGACCCCAAAAAACTTGATTAGGGTGATGGTTCACGTAGTGGGCCATCGCCCTGATAGACGG
TTTTTCGCCCTTTGACGTTGGAGTCCACGTTCTTTAATAGTGGACTCTTGTTCCAAACTGGAACAACA
CTCAACCCTATCTCGGTCTATTCTTTTGATTTATAAGGGATTTTGCCGATTTCGGCCTATTGGTTAAA
AAATGAGCTGATTTAACAAAAATTTAACGCGAATTTTAACAAAATATTAACGCTTACAATTTCCTGAT
GCGGTATTTTCTCCTTACGCATCTGTGCGGTATTTCACACCGCATAGATCCGTCGAGTTCAAGAGAAA
AAAAAAGAAAAAGCAAAAAGAAAAAAGGAAAGCGCGCCTCGTTCAGAATGACACGTATAGAATGATGC
ATTACCTTGTCATCTTCAGTATCATACTGTTCGTATACATACTTACTGACATTCATAGGTATACATAT
ATACACATGTATATATATCGTATGCTGCAGCTTTAAATAATCGGTGTCACTACATAAGAACACCTTTG
GTGGAGGGAACATCGTTGGTTCCATTGGGCGAGGTGGCTTCTCTTATGGCAACCGCAAGAGCCTTGAA
CGCACTCTCACTACGGTGATGATCATTCTTGCCTCGCAGACAATCAACGTGGAGGGTAATTCTGCTTG
CCTCTGCAAAACTTTCAAGAAAATGCGGGATCATCTCGCAAGAGAGATCTCCTACTTTCTCCCTCTGC
AAACCAAGTTCGACAACTGCGTACGGCCTGTTCGAAAGATCTACCACCGCTCTGGAAAGTGCCTCATC
CAAAGGCGCAAATCCTGATCCAAACCTTTTTACTCCACGCACGGCCCCTAGGGCCTCTTTAAATGCTT
GACCGAGAGCAATCCCGCAGTCTTCAGTGGTGTGATGGTCGTCTATGTGTAAGTCACCAATGCACTCA
ACGATTAGCGACCAGCCGGAATGCTTGGCCAGAGCATGTATCATATGGTCCAGAAACCCTATACCTGT
GTGGACGTTAATCACTTGCGATTGTGTGGCCTGTTCTGCTACTGCTTCTGCCTCTTTTTCTGGGAAGA
TCGAGTGCTCTATCGCTAGGGGACCACCCTTTAAAGAGATCGCAATCTGAATCTTGGTTTCATTTGTA
ATACGCTTTACTAGGGCTTTCTGCTCTGTCATCTTTGCCTTCGTTTATCTTGCCTGCTCATTTTTTAG
TATATTCTTCGAAGAAATCACATTACTTTATATAATGTATAATTCATTATGTGATAATGCCAATCGCT
AAGAAAAAAAAAGAGTCATCCGCTAGGGGAAAAAAAAAAAATGAAAATCATTACCGAGGCATAAAAAAA
TATAGAGTGTACTAGAGGAGGCCAAGAGTAATAGAAAAAGAAAATTGCGGGAAAGGACTGTGTTATGA
CTTCCCTGACTAATGCCGTGTTCAAACGATACCTGGCAGTGACTCCTAGCGCTCACCAAGCTCTTAAA
ACGGGAATTTATGGTGCACTCTCAGTACAATCTGCTCTGATGCCGCATAGTTAAGCCAGCCCCGACAC
CCGCCAACACCCGCTGACGCGCCCTGACGGGCTTGTCTGCTCCCGGCATCCGCTTACAGACAAGCTGT
GACCGTCTCCGGGAGCTGCATGTGTCAGAGGTTTTCACCGTCATCACCGAAACGCGCGAGACGAAAGG
GCCTCGTGATACGCCTATTTTTATAGGTTAATGTCATGATAATAATGGTTTCTTAGACGTGCGGCCGC
TCTAGAACTAGTGGATCAATTCCACGGACTATAGACTATACTAGTATACTCCGTCTACTGTACGATAC
ACTTCCGCTCAGGTCCTTGTCCTTTAACGAGGCCTTACCACTCTTTTGTTACTCTATTGATCCAGCTC
AGCAAAGGCAGTGTGATCTAAGATTCTATCTTCGCGATGTAGTAAAACTAGCTAGACCGAGAAAGAGA
CTAGAAATGCAAAAGGCACTTCTACAATGGCTGCCATCATTATTATCCGATGTGACGCTGCAGCTTCT
CAATGATATTCGAATACGCTTTGAGGAGATACAGCCTAATATCCGACAAACTGTTTTACAGATTTACG
ATCGTACTTGTTACCCATCATTGAATTTTGAACATCCGAACCTGGGAGTTTTCCCTGAAACAGATAGT

ATATTTGAACCTGTATAATAATATATAGTCTAGCGCTTTACGGAAGACAATGTATGTATTTCGGTTCC
TGGAGAAACTATTGCATCTATTGCATAGGTAATCTTGCACGTCGCATCCCCGGTTCATTTTCTGCGTT
TCCATCTTGCACTTCAATAGCATATCTTTGTTAACGAAGCATCTGTGCTTCATTTTGTAGAACAAAAA
TGCAACGCGAGAGCGCTAATTTTTCAAACAAAGAATCTGAGCTGCATTTTTACAGAACAGAAATGCAA
CGCGAAAGCGCTATTTTACCAACGAAGAATCTGTGCTTCATTTTTGTAAAACAAAAATGCAACGCGAG
AGCGCTAATTTTTCAAACAAAGAATCTGAGCTGCATTTTTACAGAACAGAAATGCAACGCGAGAGCGC
TATTTTACCAACAAAGAATCTATACTTCTTTTTTGTTCTACAAAAATGCATCCCGAGAGCGCTATTTT
TCTAACAAAGCATCTTAGATTACTTTTTTTCTCCTTTGTGCGCTCTATAATGCAGTCTCTTGATAACT
TTTTGCACTGTAGGTCCGTTAAGGTTAGAAGAAGGCTACTTTGGTGTCTATTTTCTCTTCCATAAAAA
AAGCCTGACTCCACTTCCCGCGTTTACTGATTACTAGCGAAGCTGCGGGTGCATTTTTTCAAGATAAA
GGCATCCCCGATTATATTCTATACCGATGTGGATTGCGCATACTTTGTGAACAGAAAGTGATAGCGTT
GATGATTCTTCATTGGTCAGAAAATTATGAACGGTTTCTTCTATTTTGTCTCTATATACTACGTATAG
GAAATGTTTACATTTTCGTATTGTTTTCGATTCACTCTATGAATAGTTCTTACTACAATTTTTTTGTC
TAAAGAGTAATACTAGAGATAAACATAAAAAATGTAGAGGTCGAGTTTAGATGCAAGTTCAAGGAGCG
AAAGGTGGATGGGTAGGTTATATAGGGATATAGCACAGAGATATATAGCAAAGAGATACTTTTGAGCA
ATGTTTGTGGAAGCGGTATTCGCAATATTTTAGTAGCTCGTTACAGTCCGGTGCGTTTTTGGTTTTTT
GAAAGTGCGTCTTCAGAGCGCTTTTGGTTTTCAAAAGCGCTCTGAAGTTCCTATACTTTCTAGAGAAT
AGGAACTTCGGAATAGGAACTTCAAAGCGTTTCCGAAAACGAGCGCTTCCGAAAATGCAACGCGAGCT
GCGCACATACAGCTCACTGTTCACGTCGCACCTATATCTGCGTGTTGCCTGTATATATATATACATGA
GAAGAACGGCATAGTGCGTGTTTATGCTTAAATGCGTACTTATATGCGTCTATTTATGTAGGATGAAA
GGTAGTCTAGTACCTCCTGTGATATTATCCCATTCCATGCGGGGTATCGTATGCTTCCTTCAGCACTA
CCCTTTAGCTGTTCTATATGCTGCCACTCCTCAATTGGATTAGTCTCATCCTTCAATGCTATCATTTC
CTTTGATATTGGATCATATGCATAGTACCGAGAAACTAGTGCGAAGTAGTGATCAGGTATTGCTGTTA
TCTGATGAGTATACGTTGTCCTGGCCACGGCAGAAGCACGCTTATCGCTCCAATTTCCCACAACATTA
GTCAACTCCGTTAGGCCCTTCATTGAAAGAAATGAGGTCATCAAATGTCTTCCAATGTGAGATTTTGG
GCCATTTTTTATAGCAAAGATTGAATAAGGCGCATTTTTCTTCAAAGCTGCGGCCGCACGTCAGGTGG
CACTTTTCGGGGAAATGTGCGCGGAACCCCTATTTGTTTATTTTTCTAAATACATTCAAATATGTATC
CGCTCATGAGACAATAACCGTGATAAATGCTTCAATAATATTGAAAAAGGAAGAGTATGAGTATTCAA
CATTTCCGTGTCGCCCTTATTCCCTTTTTTGCGGCATTTTGCCTTCCTGTTTTTGCTCACCCAGAAAC
GCTGGTGAAAGTAAAAGATGCTGAAGATCAGTTGGGTGCACGAGTGGGTTACATCGAACTGGATCTCA
ACAGCGGTAAGATCCTTGAGAGTTTTCGCCCCGAAGAACGTTTTCCAATGATGAGCACTTTTAAAGTT
CTGCTATGTGGCGCGGTATTATCCCGTATTGACGCCGGGCAAGAGCAACTCGGTCGCCGCATACACTA
TTCTCAGAATGACTTGGTTGAGTACTCACCAGTCACAGAAAAGCATCTTACGGATGGCATGACAGTAA
GAGAATTATGCAGTGCTGCCATAACCATGAGTGATAACACTGCGGCCAACTTACTTCTGACAACGATC
GGAGGACCGAAGGAGCTAACCGCTTTTTTGCACAACATGGGGGATCATGTAACTCGCCTTGATCGTTG
GGAACCGGAGCTGAATGAAGCCATACCAAACGACGAGCGTGACACCACGATGCCTGTAGCAATGGCAA
CAACGTTGCGCAAACTATTAACTGGCGAACTACTTACTCTAGCTTCCCGGCAACAATTAATAGACTGG
ATGGAGGCGGATAAAGTTGCAGGACCACTTCTGCGCTCGGCCCTTCCGGCTGGCTGGTTTATTGCTGA
TAAATCTGGAGCCGGTGAGCGTGGGTCTCGCGGTATCATTGCAGCACTGGGGCCAGATGGTAAGCCCT
CCCGTATCGTAGTTATCTACACGACGGGGAGTCAGGCAACTATGGATGAACGAAATAGACAGATCGCT
GAGATAGGTGCCTCACTGATTAAGCATTGGTAACTGTCAGACCAAGTTTACTCATATATACTTTAGAT
TGATTTAAAACTTCATTTTTAATTTAAAAGGATCTAGGTGAAGATCCTTTTTGATAATCTCATGACCA
AAATCCCTTAACGTGAGTTTTCGTTCCACTGAGCGTCAGACCCCGTAGAAAAGATCAAAGGATCTTCT
TGAGATCCTTTTTTTCTGCGCGTAATCTGCTGCTTGCAAACAAAAAAACCACCGCTACCAGCGGTGGT
TTGTTTGCCGGATCAAGAGCTACCAACTCTTTTTCCGAAGGTAACTGGCTTCAGCAGAGCGCAGATAC
CAAATACTGTTCTTCTAGTGTAGCCGTAGTTAGGCCACCACTTCAAGAACTCTGTAGCACCGCCTACA
TACCTCGCTCTGCTAATCCTGTTACCAGTGGCTGCTGCCAGTGGCGATAAGTCGTGTCTTACCGGGTT
GGACTCAAGACGATAGTTACCGGATAAGGCGCAGCGGTCGGGCTGAACGGGGGGTTCGTGCACACAGC

CCAGCTTGGAGCGAACGACCTACACCGAACTGAGATACCTACAGCGTGAGCTATGAGAAAGCGCCACG
CTTCCCGAAGGGAGAAAGGCGGACAGGTATCCGGTAAGCGGCAGGGTCGGAACAGGAGAGCGCACGAG
GGAGCTTCCAGGGGGAAACGCCTGGTATCTTTATAGTCCTGTCGGGTTTCGCCACCTCTGACTTGAGC
GTCGATTTTTGTGATGCTCGTCAGGGGGGCGGAGCCTATGGAAAAACGCCAGCAACGCGGCCTTTTTA
CGGTTCCTGGCCTTTTGCTGGCCTTTTGCTCACATGTTCTTTCCTGCGTTATCCCCTGATTCTGTGGA
TAACCGTATTACCGCCTTTGAGTGAGCTGATACCGCTCGCCGCAGCCGAACGACCGAGCGCAGCGAGT
CAGTGAGCGAGGAAGCGGAAGAGCGCCCAATACGCAAACCGCCTCTCCCCGCGCGTTGGCCGATTCAT
TAATGCAGCTGGCACGACAGGTTTCCCGACTGGAAAGCGGGCAGTGAGCGCAACGCAATTAATGTGAG
TTAGCTCACTCATTAGGCACCCCAGGCTTTACACTTTATGCTTCCGGCTCGTATGTTGTGTGGAATTG
TGAGCGGATAACAATTTCACACAGGAAACAGCTATGACCATGATTACGCCAAGCTCGAAATTAACCCT
CACTAAAGGGAACAAAAGCTGGTACCGGGCCGGCCGTCGGGCCGTCGAGCTTGATGGCATCGTGGTGT
CACGCTCGTCGTTTGGTATGGCTTCATTCAGCTCCGGTTCCCAACGATCAAGGCGAGTTACATGATCC
CCCATGTTGTGCAAAAAAGCGGTTAGCTCCTTCGGTCCTCCGATCGTTGTCAGAAGTAAGTTGGCCGC
AGTGTTATCACTCATGGTTATGGCAGCACTGCATAATTCTCTTACTGTCATGCCATCCGTAAGATGCT
TTTCTGTGACTGGTGAGTACTCAACCAAGTCATTCTGAGAATAGTGTATGCGGCGACCGAGTTGCTCT
TGCCCGGCGTCAACACGGGATAATACCGCGCCACATAGCAGAACTTTAAAAGTGCTCATCATTGGAAA
ACGTTCTTCGGGGCGAAAACTCTCAAGGATCTTACCGCTGTTGAGATCCAGTTCGATGTAACCCACTC
GTGCACCCAACTGATCTTCAGCATCTTTTACTTTCACCAGCGTTTCTGGGTGAGCAAAAACAGGAAGG
CAAAATGCCGCAAAAAAGGGAATAAGGGCGACACGGAAATGTTGAATACTCATACTCTTCCTTTTTCA
ATATTATTGAAGCATTTATCAGGGTTATTGTCTCATGAGCGGATACATATTTGAATGTATTTAGAAAA
ATAAACAAATAGGGGTTCCGCGCACATTTCCCCGAAAAGTGCCACCTGACGTCTAAGAAACCATTATT
ATCATGACATTAACCTATAAAAATAGGCGTATCACGAGGCCCTTTCGTCTTCAAGAATTGGGGATCTA
CGTATGGTCATTTCTTCTTCAGATTCCCTCATGGAGAAAGTGCGGCAGATGTATATGACAGAGTCGCC
AGTTTCCAAGAGACTTTATTCAGGCACTTCCATGATAGGCAAGAGAGAAGACCCAGAGATGTTGTTGT
CCTAGTTACACATGGTATTTATTCCAGAGTATTCCTGATGAAATGGTTTAGATGGACATACGAAGAGT
TTGAATCGTTTACCAATGTTCCTAACGGGAGCGTAATGGTGATGGAACTGGACGAATCCATCAATAGA
TACGTCCTGAGGACCGTGCTACCCAAATGGACTGATTGTGAGGGAGACCTAACTACATAGTGTTTAAA
GATTACGGATATTTAACTTACTTAGAATAATGCCATTTTTTTGAGTTATAATAATCCTACGTTAGTGT
GAGCGGGATTTAAACTGTGAGGACCTTAATACATTCAGACACTTCTGCGGTATCACCCTACTTATTCC
CTTCGAGATTATATCTAGGAACCCATCAGGTTGGTGGAAGATTACCCGTTCTAAGACTTTTCAGCTTC
CTCTATTGATGTTACACCTGGACACCCCTTTTCTGGCATCCAGTTTTTAATCTTCAGTGGCATGTGAG
ATTCTCCGAAATTAATTAAAGCAATCACACAATTCTCTCGGATACCACCTCGGTTGAAACTGACAGGT
GGTTTGTTACGCATGCTAATGCAAAGGAGCCTATATACCTTTGGCTCGGCTGCTGTAACAGGGAATAT
AAAGGGCAGCATAATTTAGGAGTTTAGTGAACTTGCAACATTTACTATTTTCCCTTCTTACGTAAATA
TTTTTCTTTTTAATTCTAAATCAATCTTTTTCAATTTTTTGTTTGTATTCTTTTCTTGCTTAAATCTA
TAACTACAAAAAACACATACAG
